# Nonparametric estimation via partial derivatives

**DOI:** 10.1093/jrsssb/qkae093

**Published:** 2024-09-11

**Authors:** Xiaowu Dai

**Affiliations:** Department of Statistics and Data Science, and Biostatistics, University of California, Los Angeles, CA 90095, USA

**Keywords:** derivatives, interactions, rates of convergence, reproducing kernel Hilbert space, smoothing spline ANOVA

## Abstract

Traditional nonparametric estimation methods often lead to a slow convergence rate in large dimensions and require unrealistically large dataset sizes for reliable conclusions. We develop an approach based on partial derivatives, either observed or estimated, to effectively estimate the function at near-parametric convergence rates. This novel approach and computational algorithm could lead to methods useful to practitioners in many areas of science and engineering. Our theoretical results reveal behaviour universal to this class of nonparametric estimation problems. We explore a general setting involving tensor product spaces and build upon the smoothing spline analysis of variance framework. For *d*-dimensional models under full interaction, the optimal rates with gradient information on *p* covariates are identical to those for the (d−p)-interaction models without gradients and, therefore, the models are immune to the *curse of interaction*. For additive models, the optimal rates using gradient information are $\sqrt{n}$, thus achieving the *parametric rate*. We demonstrate aspects of the theoretical results through synthetic and real data applications.

## 1 Introduction

Gradient information for complex systems arises in many areas of science and engineering. Economists estimate cost functions, where data on factor demands and costs are collected together. By Shephard’s Lemma, the demand functions are the first-order partial derivatives of the cost function (Hall & Yatchew, 2007). In actuarial science, demography provides data on the force of mortality, which, along with samples from the survival distribution, yields gradients for the survival distribution function (Frees & Valdez, 1998). In stochastic simulation, gradient estimation has been studied for a large class of problems (Glasserman, 2013). In discrete event simulation, the gradient can be estimated with a negligible computational burden compared to the effort required for obtaining a new response (Chen et al., 2013). In meteorology, wind speed and direction are gradient functions of barometric pressure and are measured over broad geographic regions (Breckling, 2012). In dynamical and time series applications, gradient information can be observed or estimated, as in biological and infectious disease modelling (Dai & Li, 2022, 2024; Ramsay et al., 2007). In traffic engineering, real-time motion sensors can record velocity in addition to positions (Solak et al., 2002).

This paper focuses on nonparametric function estimation under smoothness constraints. Rates of convergence often limit the applications of traditional nonparametric estimation methods in high-dimensional settings, where the number of covariates is large (Stone, 1980, 1982). A considerable amount of research effort has been devoted to countering this curse of dimensionality. The additive model is a popular choice (Hastie & Tibshirani, 1990; Stone, 1985). An additive model assumes that the high-dimensional function is a sum of one-dimensional functions and drops interactions among covariates in order to reduce the variability of an estimator. Stone (1985) showed that the optimal convergence rate for additive models is the same as that for univariate nonparametric estimation problems. Thus, additive models effectively mitigate the curse of dimensionality. However, additive models could be too restrictive and lead to incorrect conclusions in applications where interactions among the covariates may be present. As a more flexible alternative, smoothing spline analysis of variance (SS-ANOVA) models, the analogues of parametric ANOVA models, have attracted significant attention (Huang, 1998; Lin & Zhang, 2006; Wahba et al., 1995; Zhu et al., 2014). In particular, SS-ANOVA models include additive models as special cases. Lin (2000) proved that when interactions among covariates are in tensor product spaces, the optimal rates of convergence for SS-ANOVA models *exponentially* depend on the order of interaction. Thus, when SS-ANOVA models are used in problems that involve high-order interactions, they lead to the requirement of unrealistically large dataset sizes for reliable conclusions. We call this phenomenon the *curse of interaction*.

We develop a new approach based on partial derivatives to effectively compromise the curse of interaction. Let $\left\{(t_{i}^{\left(0\right)},y_{i}^{\left(0\right)})\;:\;i=1,\ldots ,n\right\}$ be the function data that follow a regression model,


(1) $$Y^{\left(0\right)}=f_{0}(t^{\left(0\right)})+\epsilon^{\left(0\right)}.$$

Here $\epsilon^{\left(0\right)}\in R$ is a random error, $f_{0}\;:\;X^{d}\mapsto R$ is a function of *d* covariates $t=(t_{1},\ldots ,t_{d})$, and $t^{\left(0\right)}\in X^{d}\equiv [0,1{]}^{d}$ is the design point. Write $\partial f_{0}(t)/\partial t_{j}$ as the *j*th partial derivative of $f_{0}(t)$. Let $\left\{(t_{i}^{\left(j\right)},y_{i}^{\left(j\right)})\;:\;i=1,\ldots ,n;j=1,\ldots ,p\right\}$ be the partial derivatives that follow a regression model,


(2) $$Y^{\left(j\right)}=\frac{\partial f_{0}({\;t}^{\left(j\right)})}{\partial t_{j}}+\epsilon^{\left(j\right)},\;j=1,\ldots ,p.$$

Here $\epsilon^{\left(j\right)}$s are random errors, and $t^{\left(j\right)}$s are the design points in $X^{d}$. We allow $Y^{\left(j\right)}$ to be directly observable or estimated from function data. The p∈{1,…,d} denotes the number of gradient types. Without loss of generality, we focus on the first *p* covariates in model (2). In particular, when p=d, model (2) gives the *full* gradient data. We allow for a relaxed error structure for both function and gradient data. Specifically, we assume the random errors $\epsilon^{\left(0\right)}$ and $\epsilon^{\left(j\right)}$s in models (1) and (2) to satisfy,


(3) $$\begin{array}{cc} & E[\epsilon_{i}^{\left(j\right)}]=o(n^{-1/2}),\;\text{Var}[\epsilon_{i}^{\left(j\right)}]=\sigma_{j}^{2}<\infty ,\\ & \text{Cov}[\epsilon_{i}^{\left(j\right)},\epsilon_{i^{\prime }}^{\left(j^{\prime }\right)}]=O\left(|i-i^{\prime }{|}^{-\Upsilon }\right)\;\text{ for some}\;\Upsilon >1, \end{array}$$

where $i\neq i^{\prime }$ and $j,j^{\prime }=0,1,\ldots ,p$. We assume the short-range correlation in (3) with some Υ>1. This assumption is generally valid in practice, as gradient data are often estimated by using local function data through methods such as finite-difference techniques. We provide three concrete examples in online supplementary material Appendix to elaborate on the assumption (3). Moreover, random errors in (3) can be uncentred and correlated, which are typical for estimated gradients, and include the i.i.d. errors in Hall and Yatchew (2007) as a special case.

The SS-ANOVA model (Wahba et al., 1995) amounts to the assumption that


(4) $$f_{0}(\;t)=\text{constant}+\sum_{\;j=1}^{d}f_{0j}(t_{j})+\cdots +\sum_{1\leq j_{1}<j_{2}<\cdots <j_{r}\leq d}f_{0j_{1}j_{2}\cdots j_{r}}(t_{\;j_{1}},t_{\;j_{2}},\ldots ,t_{\;j_{r}}),$$

where the component functions include main effects $f_{0j}$s, two-way interactions $f_{0j_{1}j_{2}}$s, and so on. Component functions are modelled nonparametrically, and we assume that they reside in certain reproducing kernel Hilbert spaces (RKHS, Wahba, 1990). The series on the right-hand side of (4) is truncated to some order *r* of interactions to enhance interpretability. We call $f_{0}(t)$ as *full* or *truncated* interaction SS-ANOVA model if r=d or 1≤r<d, respectively. The SS-ANOVA model (4) can be identified with space,


(5) $$H=\{1\}⊕\sum_{\;j=1}^{d}H^{\;j}⊕\cdots ⊕\sum_{1\leq j_{1}<j_{2}<\cdots <j_{r}\leq d}\left[H^{\;j_{1}}⊗H^{\;j_{2}}⊗\cdots ⊗H^{\;j_{r}}\right].$$

The components of the SS-ANOVA model in (4) are in the mutually orthogonal subspaces of H in (5). The additive model can be viewed as a special case of the SS-ANOVA model (4) by taking r=1. We assume that all component functions come from a common RKHS $\left(H_{1},\|\cdot {\|}_{H_{1}}\right)$ given by $H^{\;j}\equiv H_{1}$ for j=1,…,d. Obviously the linear model is a special example of (4) by taking r=1 and letting $H_{1}$ be the collection of all univariate linear functions defined over X. Another canonical example of $\{1\}⊕H_{1}$ is the *m*th order Sobolev space $W_{2}^{m}(X)$; see, e.g. Wahba (1990) for further examples.

We study the possibility of near-parametric rates in the general setting of SS-ANOVA models. Suppose the eigenvalues of the kernel function decay polynomially, i.e. its *ν*th largest eigenvalue is of the order $\nu^{-2m}$. Our results show that the minimax optimal rates for estimating $f_{0}$ under the *full* interaction (i.e. r=d) are


(6) $$R(n,d,r,p)=\left\{ \begin{array}{cc} {\left[n(\log\;n{)}^{1+p-d}\right]}^{-\frac{2m}{2m+1}},\; & \text{if}\;0\leq p<d,\\ n^{-\frac{2md}{(2m+1)d-2}}1_{d\geq 3}+n^{-1}(\log\;n{)}^{d-1}1_{d<3},\; & \text{if}\;p=d. \end{array}\right.$$

The rates in (6) present an interesting two-regime dichotomy between the scenarios of 0≤p<d and p=d. When 0≤p<d, the rate given by (6) matches with the minimax optimal rate for estimating a (d−p)-interaction model without gradient information (Lin, 2000). For example, when p=0 with no partial derivative data, the rate from (6) is $[n(\log\;n{)}^{1-d}{]}^{-2m/(2m+1)}$. This rate aligns with the known rate for estimating a *d*-interaction SS-ANOVA model (Lin, 2000). However, with a large *d*, this rate is heavily affected by the exponential term $(\log\;n{)}^{d-1}$, which makes the estimation challenging and leads to the curse of interaction. The inclusion of gradient data provides a substantial advantage in overcoming these challenges. For instance, when p=d−1, the rate in (6) becomes $n^{-2m/(2m+1)}$, which is the same as the optimal rate for estimating additive models without gradient information and independent of *d* (Stone, 1985). This indicates that SS-ANOVA models can be immune to the curse of interaction through the use of partial derivative data.

On the other hand, when p=d≥3, the rate in (6) becomes


$$R(n,d,r,p)=n^{-\frac{2md}{(2m+1)d-2}}.$$

This rate converges *faster* than the optimal rate for additive models $n^{-2m/(2m+1)}$. When p=d=2, the rate in (6) is $R(n,d,r,p)=n^{-1}\log\;n$. If p=d=1, the rate in (6) is the same as the *parametric* convergence rate, $R(n,d,r,p)=n^{-1}$. It is also worth noting that when $f_{0}$ has truncated interaction (i.e. r<d), the rates also improve by incorporating partial derivatives, which will be discussed in Section 3. In particular, the rate for additive models (i.e. r=1) under p=d matches with the *parametric* rate, $R(n,d,r,p)=n^{-1}$.

In the literature, various studies have outlined the construction of linear estimators for the linear functionals of $f_{0}$, with the difficulty of estimation characterized by a modulus of continuity (Cai & Low, 2005; Donoho, 1994; Donoho & Liu, 1991; Klemelä & Tsybakov, 2001). These studies are relevant to our work in two ways: first, they demonstrate the feasibility of achieving a parametric rate in estimating a univariate function $f_{0}$ from noisy derivative data, which aligns with the rate in our paper as a special case in the univariate context. Second, they provide the optimal rate for estimating partial derivatives of $f_{0}$ from observations of $f_{0}$, which differs from our target of estimating $f_{0}$ itself. Our methodology and new convergence rates bridge a gap in these studies by focusing on incorporating noisy gradient data for multivariate function estimation. A similar observation of accelerated rates has been noted earlier with *higher-order* derivatives (Hall & Yatchew, 2007, 2010). Our results suggest that such a phenomenon holds with *first-order* derivatives and applies to general SS-ANOVA models involving tensor product spaces. While our theoretical comparison primarily involves Hall and Yatchew (2007) due to its seminal importance and relevance to integrating noisy gradients in nonparametric regression, we recognize the continuous advancements in the field over the last decade. These developments include applications of joint models (1) and (2) in areas such as stochastic simulations and Gaussian process methodologies, where gradient data enhances estimation and prediction (see, e.g. Chen et al., 2013; Fu & Qu, 2014; Lim, 2024; Riihimäki & Vehtari, 2010; Wang & Berger, 2016; Zhang et al., 2023). Nonetheless, a comprehensive statistical theory explaining the benefit of incorporating noisy gradient data has been lacking. This paper develops a theoretical framework that shows how gradient data can mitigate the curse of interaction and significantly enhance the scalability of nonparametric modelling, especially for high-dimensional SS-ANOVA models.

### 1.1 Our contributions

We develop an approach and computational algorithm that incorporate partial derivatives, leading to methods useful for practitioners in many areas of science and engineering. We have derived a new theory that reveals behaviour universal to this class of nonparametric estimation problems. Our approach and theoretical results differ considerably from existing works in multiple ways, summarized as follows.

First, our results broaden the i.i.d. error structure by allowing the random errors in function data and gradient data to be biased and correlated. This relaxed assumption aligns with applications where the gradient data are estimated (Chen et al., 2013).

Second, we develop a new approach and computational algorithm in RKHS that easily incorporates gradient information. The proposed estimator also offers interpretability by providing a direct description of interactions. We find that partial derivatives can reduce interactions in terms of the minimax convergence rates.

Finally, we obtain a sharper theory on estimation with partial derivatives. We show that when p=d−1, the optimal rate for estimating *d*-dimensional SS-ANOVA models under full interaction is $n^{-2m/(2m+1)}$, which is independent of the interaction order *r*. Thus, the SS-ANOVA models are immune to the *curse of interaction* when using gradients. In contrast, Hall and Yatchew (2007) showed that when p=d−1, the convergence rate for estimating *d*-dimensional functions is $n^{-2m/(2m+d-1)}$, which suffers from the curse of dimensionality in *d*. Therefore, our results demonstrate that partial derivatives are beneficial for the scalability of nonparametric estimation in high dimensions, particularly when using SS-ANOVA models.

The rest of the sections are organized as follows: We first provide background in Section 2, and present main results in Section 3. Section 4 presents both synthetic and real data examples. Section 5 discusses related works. We conclude in Section 6. The results under other types of designs and their proofs, along with additional numerical examples, are relegated to the online supplementary material Appendix.

## 2 Background

We begin with a motivating example with partial derivatives. Then we briefly review basic facts about RKHS for the setting of our interest.

### 2.1 Motivating example

We study a stochastic simulation application to motivate models (1) and (2). Let h(t,ω) be the response of a stochastic simulation, which has a design point $t\in X^{d}$ and a random variable *ω*. It is of interest to build fast and accurate estimation for $f_{0}(t)=E_{\omega }[h(t,\omega )]$ (Chen et al., 2013; Glasserman, 2013). At each replication k=1,…,q, the stochastic simulation has a different random variable $\omega_{k}$. A user can select design $t^{\left(0\right)}$ and run the stochastic simulation to obtain a response $Y_{k}(t^{\left(0\right)})=h(t^{\left(0\right)},\omega_{k})=f_{0}(t^{\left(0\right)})+\epsilon_{k}^{\left(0\right)}$, where $\epsilon_{k}^{\left(0\right)}$ is i.i.d. centred simulation noise. In practice, it is common to average responses to reduce the variance of simulation noises, i.e. let $Y^{\left(0\right)}=[Y_{1}(t^{\left(0\right)})+Y_{2}(t^{\left(0\right)})+\cdots +Y_{q}(t^{0})]/q$, where *q* is the number of simulation replications and is at the order of hundreds or thousands. Then the response $Y^{\left(0\right)}$ follows model (1), where $\epsilon^{\left(0\right)}$ is the averaged simulation noise. Under regularity conditions ensuring the interchange of expectation and differentiation (L’Ecuyer, 1990), the infinitesimal perturbation analysis (IPA) gives the gradient estimator of $f_{0}(t)$ that follows model (2),


$$Y^{\left(j\right)}=\frac{\partial }{\partial t_{j}}h({\;t}^{\left(j\right)},\omega ),\;{\;t}^{\left(j\right)}\in X^{d},\;j=1,\ldots ,p,\;1\leq p\leq d.$$

**Figure 1.**
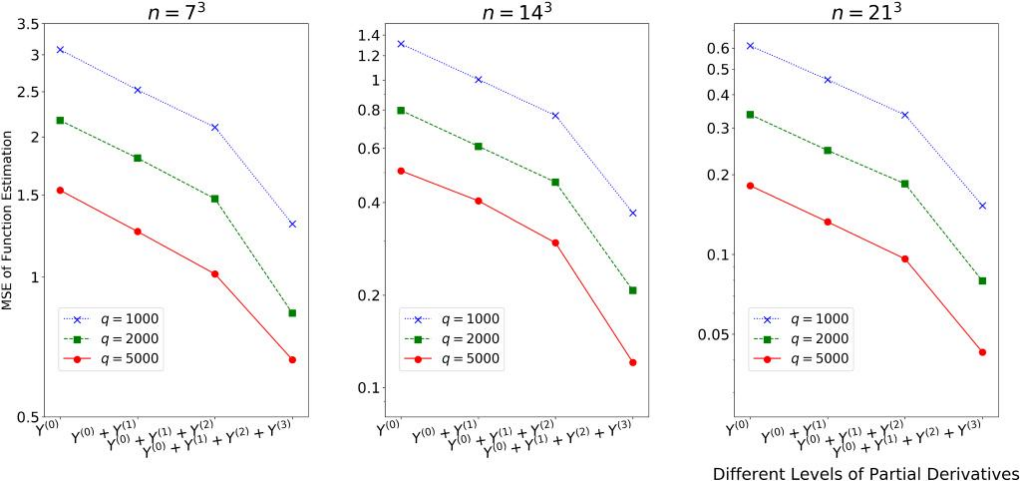
Estimation error of our estimator incorporating different levels of gradient information, for the stochastic simulation example. The *y*-axis is in the log scale.

Moreover, the IPA estimators are unbiased, $E_{\omega }[Y^{\left(j\right)}]=\partial f_{0}/\partial t_{j}$ (Glasserman, 2013). We provide details of our stochastic simulation in Section 4.1. The results are reported in Figure 1, which shows mean-squared errors (MSEs) for varying sample size *n*, replication number *q*, and different methods. Those include stochastic kriging with function data (i.e. p=0), our estimator with function and one type of gradient data (i.e. p=1), two types of gradient data (i.e. p=2), the full gradient data (i.e. p=3). A significant decrease in MSEs is observed when incorporating partial derivatives. Moreover, the computational cost for obtaining the gradient estimator is relatively low, as calculating the IPA estimator $Y^{\left(j\right)}$ does not need additional replication of the simulation. In contrast, getting a new function response $Y^{\left(0\right)}$ requires *q* new replications of the simulation, and each replication could incur a high cost.

### 2.2 Reproducing kernel for partial derivatives

We briefly review some basic facts about RKHS. Interested readers are referred to Aronszajn (1950) and Wahba (1990) for further details. Let *K* be a Mercer kernel that is a symmetric positive semidefinite and square-integrable function on X×X. It can be uniquely identified with the Hilbert space $H_{1}$ that is the completion of $\left\{\sum_{i=1}^{N}c_{i}K(t_{i},\cdot \;)\;:\;t_{i}\in X,c_{i}\in R,i=1,\ldots ,N\right\}$ under the inner product $\left\langle \sum_{i}c_{i}K(t_{i},\cdot \;),\sum_{j}c_{j}K(t_{j},\cdot \;)\rangle_{H_{1}}=\sum_{i,j}c_{i}c_{j}K(t_{i},t_{j}\right)$. Most commonly used kernels are differentiable, which we shall assume in what follows. In particular, we assume that


(7) $$\frac{\partial^{2}}{\partial t\partial t^{\prime }}K(t,t^{\prime })\in C(X\times X).$$

where C(⋅) is the space of continuous functions. Let the kernel $K_{d}((t_{1},\ldots ,t_{d}{)}^{⊤},(t_{1}^{\prime },\ldots ,t_{d}^{\prime }{)}^{⊤})\;=$  $K(t_{1},t_{1}^{\prime })\cdots K(t_{d},t_{d}^{\prime })$. Then $K_{d}(\cdot ,\cdot \;)$ is the kernel corresponding to the RKHS $\left(H,\|\cdot {\|}_{H}\right)$ in (5); see, e.g. Aronszajn (1950). The condition (7) together with the continuity of $\langle \cdot ,\cdot \rangle_{H}$ yield that for any g∈H, $\partial g(t)/\partial t_{j}=\partial \langle g,K_{d}(t,\cdot \;)\rangle_{H}/\partial t_{j}=\langle g,\partial K_{d}(t,\cdot \;)/\partial t_{j}\rangle_{H}$. Thus, the gradient $\partial g(t)/\partial t_{j}$ is a bounded linear functional in H and has a representer $\partial K_{d}(t,\cdot \;)/\partial t_{j}$. By Mercer’s theorem (Riesz, 1955), the kernel function *K* admits an eigenvalue decomposition:


(8) $$K(t,t^{\prime })=\sum_{\nu \geq 1}\lambda_{\nu }\psi_{\nu }(t)\psi_{\nu }(t^{\prime }),$$

where $\lambda_{1}\geq \lambda_{2}\geq \cdots \geq 0$ are eigenvalues and $\left\{\psi_{\nu }\;:\;\nu \geq 1\right\}$ are the corresponding eigenfunctions. For example, $\lambda_{\nu }≍\nu^{-2m}$ for $W_{2}^{m}(X)$ under the Lebesgue measure (Wahba, 1990), which will be also discussed in online supplementary material Appendix.

## 3 Main results

In this section, we present a new approach for nonparametric estimation via partial derivatives and develop a fast algorithm. We also derive a new theory and show a convergence behaviour universal to this class of estimation problems.

### 3.1 Estimation via partial derivatives

We introduce a method that merges function and derivative information for better estimation. When the function $f_{0}$ in (4) is smooth in H, we add the empirical loss of partial derivatives as a penalty. Combining these information, we derive the function ${\hat{f}}_{n}$ that meets the smoothness criteria and aligns closely with the observed data,


(9) $${\hat{\;f}}_{n}=\underset{\|f{\|}_{H}\leq R_{n}}{\text{arg min}}\;\left\{\frac{1}{n}\sum_{i=1}^{n}{\left[y_{i}^{\left(0\right)}-f({\;\;t}_{i}^{\left(0\right)})\right]}^{2}+\sum_{\;j=1}^{p}w_{j}\cdot \frac{1}{n}\sum_{i=1}^{n}{\left[y_{i}^{\left(j\right)}-\frac{\partial f}{\partial t_{j}}({\;\;t}_{i}^{\left(j\right)})\right]}^{2}\right\}.$$

Here $R_{n}\geq 0$ is an appropriately chosen Hilbert radius, and $w_{j}\geq 0$ is a weight parameter, where a natural choice is $w_{j}=\sigma_{0}^{2}/\sigma_{j}^{2}$. If $\sigma_{0}^{2}$ and $\sigma_{j}^{2}$ are unknown, we can replace them with consistent estimators for variances (Hall et al., 1990). The concept of derivative-based penalty has also been employed in the generalized profiling approach of Ramsay et al. (2007), which derives a penalty by comparing the derivative of the estimated function to a trajectory generated by ordinary differential equations (ODEs). However, the approach in (9) is different by directly comparing the derivative of the estimated function with either observed or estimated derivatives at discrete data points, which avoids the complexities associated with ODE computations. The following theorem gives a closed-form solution to (9).

Theorem 1 Assume that kernel *K* satisfies the differentiability condition (7). Then, for any $R_{n}\geq 0$, there exists a minimizer ${\hat{\;f}}_{n}(t)$ of (9) in a finite-dimensional space,

$${\hat{\;f}}_{n}(\;t)=\sum_{i=1}^{n}\alpha_{i0}K_{d}({\;t}_{i}^{\left(0\right)},\;t)+\sum_{\;j=1}^{p}\sum_{i=1}^{n}\alpha_{ij}\frac{\partial K_{d}}{\partial t_{j}}({\;t}_{i}^{\left(j\right)},\;t),$$

where the coefficients $\alpha_{j}=(\alpha_{1j},\ldots ,\alpha_{nj}{)}^{⊤}\in R^{n}$ for j=0,1,…,p.

This theorem is a generalization of the well-known representer lemma for smoothing splines (Wahba, 1990). It in effect turns an infinity-dimensional optimization problem into an optimization problem over finite number of coefficients. We will devise a fast algorithm for this optimization in Section 3.2 and show its scalability for large data.

The estimator (9) is different from existing methods of incorporating gradients. For example, Morris et al. (1993) proposed a stationary Gaussian process to combine noiseless gradients, whereas the estimator (9) applies to noisy gradients. Hall and Yatchew (2007) studied a regression-kernel estimator to incorporate noisy derivatives and required special structures on the observed derivatives. However, the estimator (9) can incorporate all types of estimated or observed partial derivatives. Hall and Yatchew (2010) used a series-type estimator but could have a curse of dimensionality problem. In contrast, (9) can scale up to a large dimension *d*. Chen et al. (2013) considered a stochastic kriging method, where the correlation coefficients between gradients and function data are required to be estimated. Differently, it is unnecessary to estimate such correlations for implementing (9). Moreover, we will demonstrate that the estimator (9) outperforms competing alternatives through numerical examples in Section 4.

### 3.2 Computational algorithm

We now develop a fast algorithm for computing the minimizer ${\hat{\;f}}_{n}(t)$ in Theorem 1. Note that ${\hat{\;f}}_{n}(t)$ can be further written as, for any $t\in X^{d}$,


(10) $${\hat{\;f}}_{n}(\;t)={\tilde{\Psi }}_{d}(\;\;t{)}^{⊤}{\tilde{\;c}}_{0}+\sum_{\;j=1}^{p}\frac{\partial {\tilde{\Psi }}_{d}(\;t{)}^{⊤}{\tilde{\;c}}_{j}}{\partial t_{j}},$$

where ${\tilde{\Psi }}_{d}(t)=[{\tilde{\Psi }}^{{⊗}_{1}}(t_{1}{)}^{⊤},\ldots ,{\tilde{\Psi }}^{{⊗}_{1}}(t_{d}{)}^{⊤},{\tilde{\Psi }}^{{⊗}_{2}}(t_{1},t_{2}{)}^{⊤},\ldots ,{\tilde{\Psi }}^{{⊗}_{r}}(t_{d-r+1},t_{d-r+2},\ldots ,t_{d}{)}^{⊤}{]}^{⊤}$. The column vector ${\tilde{\Psi }}^{{⊗}_{1}}(t)$ has the *ν*th element equal to $\sqrt{\lambda_{\nu }}\psi_{\nu }(X)$ for ν≥1. The vector $\Psi^{{⊗}_{2}}(t_{i},t_{j})=\Psi^{{⊗}_{1}}(t_{i})⊗\Psi^{{⊗}_{1}}(t_{j})$ is generated by the Kronecker product that combines two vectors $\Psi^{{⊗}_{1}}(t_{i})$ and $\Psi^{{⊗}_{1}}(t_{j})$ into a single vector, where for each element in the first vector $\Psi^{{⊗}_{1}}(t_{i})$, we multiply the entire second vector $\Psi^{{⊗}_{1}}(t_{j})$ by that element, and the resulting vectors from each multiplication are then concatenated, forming a long vector that captures all pairwise interactions between the elements of $\Psi^{{⊗}_{1}}(t_{i})$ and $\Psi^{{⊗}_{1}}(t_{j})$. Similarly, ${\tilde{\Psi }}^{{⊗}_{r}}(t_{d-r+1},t_{d-r+2},\ldots ,t_{d})=\Psi^{{⊗}_{1}}(t_{d}-r+1)⊗\Psi^{{⊗}_{1}}(t_{d}-r+2)⊗\cdots ⊗\Psi^{{⊗}_{1}}(t_{d})$ is the Kronecker product of the *r* corresponding vectors. Here ${\tilde{c}}_{j}=[{\tilde{\Psi }}_{d}(t_{1}^{\left(j\right)}),\ldots ,{\tilde{\Psi }}_{d}(t_{n}^{\left(j\right)})]\alpha_{j}$ is the infinite-dimensional coefficient vector, where j=0,1,…,p.

The key idea is to employ the random feature mapping (Dai et al., 2023; Rahimi & Recht, 2007) to approximate the kernel function, which enables us to construct a projection operator between the RKHS and the original predictor space. Specifically, if the kernel functions that generate $H_{1}$ are shift-invariant, i.e. $K(t,t^{\prime })=K(t-t^{\prime })$, and integrate to one, i.e. $\int_{X}K(t-t^{\prime })d(t-t^{\prime })=1$, then the Bochner’s theorem (Bochner, 1934) states that such kernel functions satisfy the Fourier expansion:


$$K(t-t^{\prime })=\int_{R}p(w)\exp\;\left\{\sqrt{-1}w(t-t^{\prime })\right\}\;dw,$$

where p(w) is a probability density defined by


$$p(w)\;=\int_{X}K(t)e^{-2\pi \sqrt{-1}wt}\;dt.$$

We note that many kernel functions are shift-invariant and integrate to one. Examples include the Matérn kernel, $K(t,t^{\prime })={\tilde{\tau }}_{1}(1+|t-t^{\prime }|/\tau_{1}+|t-t^{\prime }{|}^{2}/3\tau_{1}^{2})e^{-|t-t^{\prime }|/\tau_{1}}$, the Laplacian kernel, $K(X,X^{\prime })={\tilde{\tau }}_{2}e^{-|X-X^{\prime }|/\tau_{2}}$, the Gaussian kernel, $K(X,X^{\prime })={\tilde{\tau }}_{3}e^{-\tau_{3}^{2}|X-X^{\prime }{|}^{2}/2}$, and the Cauchy kernel, $K(X,X^{\prime })={\tilde{\tau }}_{4}(1+\tau_{4}^{2}|X-X^{\prime }{|}^{2}{)}^{-1}$, where ${\tilde{\tau }}_{1},{\tilde{\tau }}_{2},{\tilde{\tau }}_{3},{\tilde{\tau }}_{4}$ are the normalization constants, and $\tau_{1},\tau_{2},\tau_{3},\tau_{4}$ are the scaling parameters. It is then shown that (Rahimi & Recht, 2007) the minimizer in Theorem 1 can be approximated by,


$${\hat{\;f}}_{n}(t)=\Psi_{d}(t{)}^{⊤}{\;c}_{0}+\sum_{j=1}^{p}\frac{\partial \Psi_{d}(\;t{)}^{⊤}{\;c}_{j}}{\partial t_{j}},$$

where $\Psi_{d}(t)=[\Psi^{{⊗}_{1}}(t_{1}{)}^{⊤},\ldots ,\Psi^{{⊗}_{1}}(t_{d}{)}^{⊤},\Psi^{{⊗}_{2}}(t_{1},t_{2}{)}^{⊤},\ldots ,\Psi^{{⊗}_{r}}(t_{d-r+1},t_{d-r+2},\ldots ,t_{d}{)}^{⊤}{]}^{⊤}$, and $\Psi^{{⊗}_{1}}(t_{j})=$  $[{\tilde{\psi }}_{1}(t_{j}),\ldots ,{\tilde{\psi }}_{s}(t_{j}){]}^{⊤}\in R^{s}$ is a vector of *s* Fourier bases with the frequencies drawn from the density p(w), i.e.


(11) $$\begin{array}{cc} & \omega_{\;j,\nu }\;\overset{i.i.d.}{∼}\;p(\omega ),\;b_{\;j,\nu }\;\overset{i.i.d.}{∼}\;\mathrm{Uniform}[0,2\pi ],\\ & {\tilde{\psi }}_{\nu }(t_{j})=\sqrt{\frac{2}{s}}\cos\;(t_{j}\omega_{\;j,\nu }+b_{\;j,\nu }),\;j=1,\ldots ,d,\;\nu =1,\ldots ,s, \end{array}$$

and $\Psi^{{⊗}_{2}}(t_{i},t_{j})=\Psi^{{⊗}_{1}}(t_{i})⊗\Psi^{{⊗}_{1}}(t_{j})\in R^{s^{2}}$, and so on. We write the augmented random feature vector as,


(12) $$\Psi_{(p+1)d}(t)={\left(\Psi_{d}(t{)}^{⊤},\frac{\partial \Psi_{d}(t{)}^{⊤}}{\partial t_{1}},\ldots ,\frac{\partial \Psi_{d}(t{)}^{⊤}}{\partial t_{p}}\right)}^{⊤}.$$

Then the minimizer in Theorem 1 can be approximated by,


(13) $${\hat{\;f}}_{n}(\;t)=\Psi_{(p+1)d}(t{)}^{⊤}{\;c}_{(p+1)d}.$$

We estimate the coefficient vector $c_{(p+1)d}=(c_{0}^{⊤},c_{1}^{⊤},\ldots ,c_{\;p}^{⊤}{)}^{⊤}$ by minimizing the following convex objective function,


(14) $$\frac{1}{n}\sum_{i=1}^{n}{\left[y_{i}^{\left(0\right)}-{\hat{\;f}}_{n}({\;t}_{i}^{\left(0\right)})\right]}^{2}+\sum_{\;j=1}^{p}w_{j}\cdot \frac{1}{n}\sum_{i=1}^{n}{\left[y_{i}^{\left(j\right)}-\frac{\partial {\hat{\;f}}_{n}}{\partial t_{j}}({\;t}_{i}^{\left(j\right)})\right]}^{2}+\;\lambda \sum_{\;j=0}^{p}\|c_{j}{\|}_{2}^{2},$$

where λ≥0 is the penalty parameter. We remark that the penalty in (14) is different from the penalty in kernel ridge regression (Wainwright, 2019), which takes the form $\|\Psi_{(p+1)d}(t{)}^{⊤}c_{(p+1)d}{\|}_{H}^{2}$. Since the random feature mapping generally cannot form an orthogonal basis, there is no closed-form representation of the RKHS norms $\|\Psi_{(p+1)d}(t{)}^{⊤}c_{(p+1)d}{\|}_{H}^{2}$ in our setting. As a result, the kernel ridge regression penalty is difficult to implement, and instead we adopt the $L_{2}$ penalty in (14) that is easy for computing. We choose the smoothing parameter *λ* in (14) by generalized cross-validation (GCV) (Golub et al., 1979). Let A(λ) be the influence matrix as $\hat{y}=A(\lambda )y$, where *y* is the vector of function and gradient data $y=(y_{1}^{\left(0\right)},\ldots ,y_{n}^{\left(0\right)},\ldots ,y_{1}^{\left(p\right)},\ldots ,y_{n}^{\left(p\right)}{)}^{⊤}$, and $\hat{y}$ is the estimate, $\hat{y}=({\hat{\;f}}_{n}(t_{1}^{\left(0\right)}),\ldots ,{\hat{\;f}}_{n}(t_{n}^{\left(0\right)}),\ldots ,\partial {\hat{\;f}}_{n}/\partial t_{p}(t_{1}^{\left(p\right)}),\ldots ,\partial {\hat{\;f}}_{n}/\partial t_{p}(t_{n}^{\left(p\right)}){)}^{⊤}$. Then GCV selects λ≥0 by minimizing the following risk,


$$\text{GCV}(\lambda )=\frac{\|\hat{y}-y{\|}^{2}}{[n^{-1}\text{tr}(I-A(\lambda )){]}^{2}}.$$

The use of random feature mapping achieves potentially substantial dimension reduction. More specifically, the estimator in (13) only requires to learn the finite-dimensional coefficient $c_{(p+1)d}$, compared to the estimator in (10) that involves an infinite-dimensional vector ${\tilde{c}}_{j}$ for j=0,1,…,p. It is known that the random feature mapping obtains the optimal bias-variance tradeoff if *s* scales at a certain rate and s/n→0 when *n* grows (Rudi & Rosasco, 2017). We note that the random feature mapping also efficiently reduces the computational complexity. Given any (d,r,p), the computation complexity of the estimator in (13) is only $O(ns^{2})$, compared to the computation complexity of the kernel estimator in Theorem 1 that is $O(n^{3})$. The saving of the computation is substantial if s/n→0 as *n* grows.

**Algorithm 1:** Estimation via partial derivatives.

1: **Input**: Function data $\left\{(t_{i}^{\left(0\right)},y_{i}^{\left(0\right)})\;:\;i=1,\ldots ,n\right\}$, partial derivatives $\left\{(t_{i}^{\left(j\right)},y_{i}^{\left(j\right)})\;:\;i=1,\ldots ,n;j=1,\ldots ,p\right\}$, weight parameters $\left\{w_{j}\;:\;j=1,\ldots ,p\right\}$.
2: **Step 1**: Sample *d* of i.i.d. *s*-dimensional random features $\{w_{\nu },b_{\nu }{\}}_{\nu =1}^{s}$ by (11), and construct the augmented random feature vector $\Psi_{(p+1)d}(t)$ by (12).
3: **Step 2**: Solve the coefficient vector $c_{(p+1)d}$ by (14).
4: **Output**: Function estimate ${\hat{\;f}}_{n}(t)$ in (13).

We summarize the above estimation procedure in Algorithm 1.

### 3.3 Minimax optimality

We show that our proposed estimator achieves optimality. Suppose that design points $t^{\left(0\right)}$ in (1) and $t^{\left(j\right)}$s in (2) are independently drawn from $\Pi^{\left(0\right)}$ and $\Pi^{\left(j\right)}$s, respectively, where $\Pi^{\left(0\right)}$ and $\Pi^{\left(j\right)}$s have densities bounded away from zero and infinity. We first present a minimax lower bound in the presence of partial derivatives.

Theorem 2 Assume that $\lambda_{\nu }≍\nu^{-2m}$ for some m>3/2 and the kernel *K* admits the decomposition in (8). Under the regression models (1) and (2) where $f_{0}$ follows the SS-ANOVA model (4) and $\|f{\|}_{H}\leq R_{n}$. Then under the error structure (3), there exists a constant *c* such that

liminfn→∞inff~supf0∈HP{∫Xd[f~(t)−f0(t)]2dt≥c([n(logn)1−(d−p)∧r]−2m2m+110≤p<d+[n−2mr(2m+1)r−21r≥3+n−1(logn)r−11r<3]1p=d∫Xd{f~(t)−f0(t)}2dt)}>0,

where the infimum of $\tilde{f}$ is taken over all measurable functions of the data.

This lower bound is new in the literature, and its proof is established via Fano’s lemma (Tsybakov, 2009). Next, we show that the lower bound is attainable via our estimator.

Theorem 3 Assume that $\lambda_{\nu }≍\nu^{-2m}$ for some m>3/2 and the kernel *K* admits the decomposition in (8). Under the regression models (1) and (2) where $f_{0}$ follows the SS-ANOVA model (4) and $\|f{\|}_{H}\leq R_{n}$. Then under the error structure (3) and with the number of random features in (11) set to s=O(nlogn), the estimator ${\hat{\;f}}_{n}$ in (13) satisfies

limC→∞lim supn→∞supf0∈HP{∫Xd[f^n(t)−f0(t)]2dt≤C([n(logn)1−(d−p)∧r]−2m2m+110≤p<d+[n−2mr(2m+1)r−21r≥3+n−1(logn)r−11r<3]1p=d[n(logn)1−(d−p)∧r]−2m/(2m+1))∫Xd{f~(t)−f0(t)}2dt}=1.

Here the tuning parameter *λ* in (14) is chosen by $\lambda ≍[n(\log\;n{)}^{1-(d-p)\wedge r}{]}^{-2m/(2m+1)}$ when 0≤p<d, and $\lambda ≍n^{-(2mr-2)/[(2m+1)r-2]}$ when p=d,r≥3, and $\lambda ≍(n\log\;n{)}^{-(2m-1)/2m}$ when p=d,r=2, and $\lambda ≍n^{-(m-1)/m}$ when p=d, r=1.

The proof of Theorem 3 relies on several techniques from empirical process and stochastic process theory, including the linearization method and operator gradients. In our analysis of SS-ANOVA models incorporating gradient information, unlike the approach by Lin (2000) which lacks such data, we have developed a method for the simultaneous diagonalization of two positive definite kernels: one including only function data, and the other incorporating both function and gradient data. We have obtained sharper results on the minimax rates of convergence than those in Lin (2000). Moreover, Theorem 3 demonstrates that the optimal rate in (15) can be achieved with the random feature estimator ${\hat{\;f}}_{n}(t)$, as defined in (13). This represents another contribution compared to Lin (2000).

Theorems 2 and 3 together immediately imply that the minimax optimal rate for estimating $f_{0}\in H$ is


(15) $$\begin{array}{rl} & {\left[n(\log\;n{)}^{1-(d-p)\wedge r}\right]}^{-\frac{2m}{2m+1}}1_{0\leq p<d}+\left[n^{-\frac{2mr}{(2m+1)r-2}}1_{r\geq 3}+n^{-1}(\log\;n{)}^{r-1}1_{r<3}\right]1_{\;p=d}. \end{array}$$

This result connects with two strands of literature–estimating SS-ANOVA models without gradient information, and estimating nonparametric functions using derivatives.

First, in the case of estimating SS-ANOVA models without gradient information, the result in (15) recovers the rate known in the literature (see, e.g. Lin, 2000),


(16) $${\left[n(\;\log\;n{)}^{1-r}\right]}^{-\frac{2m}{2m+1}}.$$

For a high-order interaction *r*, the exponential term $(\;\log\;n{)}^{r-1}$ in (16) introduces the *curse of interaction* and makes the SS-ANOVA models impractical. Surprisingly, the result in (15) shows that incorporating gradient data mitigates this curse. For example, when d−r≤p≤d−1, the rate given by (15) becomes,


(17) $${\left[n(\;\log\;n{)}^{1-(d-p)}\right]}^{-\frac{2m}{2m+1}}.$$

This rate is identical to the minimax optimal rate for estimating a (d−p)-interaction model without gradient information (Lin, 2000). Increasing *p* types of gradient data to (p+1) accelerates the rate at the order of $(\;\log\;n{)}^{-2m/(2m+1)}$, where p≥d−r and p+1≤d−1. Moreover, when p=d−1, the rate given by (17) is $n^{-2m/(2m+1)}$, which coincides with the optimal rate for estimating additive models without gradient information (Stone, 1985). The result in (15) indicates a phase transition from 0≤p<d to p=d. Specifically, the rate with full gradient p=d is further improved compared to that with p≤d−1. We also note that when the SS-ANOVA models have full interaction with r=d, the result in (15) yields the special result in (6).

Second, in the case of estimating functions using derivatives, Hall and Yatchew (2007) pioneered the proposal of a regression-kernel method for incorporating derivative data under random design and i.i.d. errors. Hall and Yatchew (2007) proved that with first-order partial derivatives, their estimator achieves the rate $n^{-2m/(2m+d-1)}$ for general Hölder spaces (e.g. their Theorem 3). This rate converges *slower* than the rate given by (15) when d≥2, and it suffers from the curse of dimensionality when *d* is large. In contrast, our work, employing a reproducing kernel approach within the function space of SS-ANOVA models, a subspace of Hölder spaces characterized by a tensor product structure, achieves the *improved* convergence rate in (15). This new result shows the practical value of gradient information in enhancing the scalability of nonparametric modelling, especially in high-dimensional settings typical of SS-ANOVA models.

### 3.4 Extension of the main results

We discuss various ways to extend the optimal rates established in Theorems 2 and 3. For instance, these rates can be adapted to scenarios where the function values and partial derivatives have different sample sizes. Let $n_{j}$ denote the sample size for the dataset $\left\{(t_{i}^{\left(j\right)},y_{i}^{\left(j\right)})\;:\;i=1,\ldots ,n_{j}\right\}$, where j=0,1,…,p. By applying the same arguments as in our proofs, the rate in these theorems can be expressed as


$$\begin{array}{rl} & \min\{{\left[n_{0}(\;\log\;n_{0}{)}^{1-r}\right]}^{-\frac{2m}{2m+1}},\;{\left[\left(\min_{\;j\geq 1}n_{j}\right){\left(\log\;\left(\min_{\;j\geq 1}n_{j}\right)\right)}^{1-(d-p)\wedge r}\right]}^{-\frac{2m}{2m+1}}1_{0\leq p<d}\\ & \;+\left[{\left(\min_{\;j\geq 1}n_{j}\right)}^{-\frac{2mr}{(2m+1)r-2}}1_{r\geq 3}+{\left(\min_{\;j\geq 1}n_{j}\right)}^{-1}{\left(\log\;\left(\min_{\;j\geq 1}n_{j}\right)\right)}^{r-1}1_{r<3}\right]1_{\;p=d}\}. \end{array}$$

This rate is essentially the minimum of two scenarios: the rate obtained by replacing (15) in terms of the value of $\min_{\;j\geq \;1}n_{j}$ and the conventional rate (16) based solely on the function data with $n_{0}$ samples. If the sample size $n_{0}$ for noisy function values is significantly smaller than $\min_{\;j\geq \;1}n_{j}$, the optimal rate in (15) still holds with $n=\min_{\;j\geq \;1}n_{j}$. In this case, the noisy function values contribute to anchoring the absolute level of the function, making function estimation identifiable. Conversely, if the dataset of noisy function values alone is substantially large, i.e. $n_{0}$ is much greater than $\min_{\;j\geq \;1}n_{j}$, the convergence rate by Theorems 2 and 3 aligns with the conventional rate (16) based solely on the noisy function values.

The optimal rates in Theorems 2 and 3 also apply under deterministic designs, where the design points $t^{\left(0\right)}$ in (1) and $t^{\left(j\right)}$ in (2) are equally spaced in $X^{d}$, rather than independently drawn from distributions $\Pi^{\left(0\right)}$ and $\Pi^{\left(j\right)}$, respectively. The results for deterministic designs are given in online supplementary material Appendix S1. Additionally, the optimal rates are valid under a more general error assumption than (3). Specifically, they hold when $\text{Var}(\epsilon_{i}^{\left(j\right)})=\sigma_{j}^{2}+o(n^{-1/2})$. A rigorous proof of Theorem 3 under this general error assumption follows a similar argument to that of the original proof.

Finally, we discuss additive models, which can be regarded as a special case of the SS-ANOVA model (4) by setting r=1. In this scenario, with gradient data available where p=d, Theorems 2 and 3 suggest that the estimation of additive models can achieve the parametric rate of $n^{-1}$, which is a significant improvement over the traditional optimal rate of $n^{-2m/(2m+1)}$ typically achieved without gradient information (Stone, 1985). We provide intuition behind achieving the parametric rate in additive models to illustrate the benefits of incorporating gradient information in statistical estimations. Heuristically, for a univariate function $f_{0}$, the problem of estimating $f_{0}$ with noisy gradient data is analogous to settings where $f_{0}$ is observed with noise, but the integral of $f_{0}$ is the estimation target, which can achieve the parametric rate $n^{-1}$ through nonlocal averaging (Donoho, 1994; Donoho & Liu, 1991). This analogy suggests that the availability of gradient data eliminates the need for smoothing or local averaging, typically necessary in nonparametric estimation, thus allowing for a faster parametric rate. In the case of multivariate additive models, where $f_{0}=f_{01}+\cdots +f_{0d}$, gradient data effectively provides observations on the derivatives of each component function, $df_{0j}(t_{j})/dt_{j}$, enabling the estimation of each component at the parametric rate and, consequently, the entire function $f_{0}$.

## 4 Applications

In this section, we demonstrate the aspects of our method and theory via various applications. We study a stochastic simulation example in Section 4.1, and an economics example in Section 4.2. We analyse a real data experiment of ion channel in Section 4.3.

### 4.1 Call option pricing with stochastic simulations

We discussed a motivating example of stochastic simulation in Section 2.1. Now we consider a detailed stochastic simulation of the call option pricing. The Black–Scholes stochastic differential equation is commonly used to model stock price $S_{T}$ at time *T* through


$$dS_{T}=r_{*}S_{T}dT+\sigma_{*}S_{T}dW_{T},T\geq 0,$$

where $W_{T}$ is the Wiener process, $r_{*}$ is the risk-free rate, and $\sigma_{*}$ is the volatility of the stock price. The equation has a closed-form solution: $S_{T}=S_{0}\exp\;\{(r_{*}-\frac{1}{2}\sigma_{*}^{2})T+\sigma_{*}\sqrt{T}\omega \}$ with the standard normal variable *ω* and initial stock price $S_{0}$. The European call option is the right to buy a stock at the prespecified time *T* with a prespecified price $P_{0}$. The value of the European option is


$$h(t,\omega )=e^{-r_{*}T}(S_{T}-P_{0}{)}_{+},$$

where $t=(S_{0},r_{*},\sigma_{*})$. Our goal is to estimate the expected net present value of the option with fixed *T* and $P_{0}$: $f_{0}(t)=E_{\omega }[h(t,\omega )]$. It can be seen that $f_{0}(t)$ follows the SS-ANOVA model (4). In the experiment, we fix T=1, $P_{0}=100$, and choose the design t from equally spaced points from $S_{0}\in [80,120]$, $r_{*}\in [0.01,0.05]$, and $\sigma_{*}\in [0.2,1]$ with the sample size $n=7^{3},14^{3},21^{3}$. The end points of each interval are always included. We set the number of random feature s=n/10 for constructing the random feature estimator in (13). To address the impact of stochastic simulation noise, we simulate q=1,000,2,000,5,000 i.i.d. replications of $S_{T}$ at each design point and then average the responses. Independent sampling is used across design points. It is known that IPA estimators for the gradient: $\partial f_{0}/\partial S_{0}$, $\partial f_{0}/\partial r_{*}$, $\partial f_{0}/\partial \sigma_{*}$ are given by Glasserman (2013),


(18) $$\begin{array}{rl} Y^{\left(1\right)} & =e^{-r_{*}T}\frac{S_{T}}{S_{0}}\cdot 1\{S_{T}\geq P_{0}\},\\ Y^{\left(2\right)} & =-TY^{\left(0\right)}+e^{-r_{*}T}TS_{T}\cdot 1\{S_{T}\geq P_{0}\},\\ Y^{\left(3\right)} & =e^{-r_{*}T}\frac{1}{\sigma_{*}}\left[\log\;\left(\frac{S_{T}}{S_{0}}\right)-\left(r_{*}+\frac{1}{2}\sigma_{*}^{2}\right)T\right]S_{T}\cdot 1\{S_{T}\geq P_{0}\}. \end{array}$$

The IPA estimators (18) are unbiased, $E_{\omega }[Y^{\left(1\right)}]=\partial f_{0}/\partial S_{0},E_{\omega }[Y^{\left(2\right)}]=\partial f_{0}/\partial r_{*}$, $E_{\omega }[Y^{\left(3\right)}]=\partial f_{0}/\partial \sigma_{*}$. We show in online supplementary material Appendix S2 that the error assumption (3) holds for IPA estimators in (18). In this example, obtaining function data at a new design point requires the generation of *q* new random numbers and the computation of $S_{T}$ for each of these *q* simulation replications. In contrast, the gradient estimator given by (18) can be obtained at a negligible cost and without a new simulation.

####  

##### Comparison with existing method

Stochastic kriging (Ankenman et al., 2010; Chen et al., 2013) is a popular method for the mean response estimation of a stochastic simulation. We compare the results of our estimator (13) incorporating gradient information and the stochastic kriging method without gradient. Consider the tensor product Matérn kernel,


(19) $$\prod_{\;j=1}^{3}\left(1+|t_{j}-t_{j}^{\prime }|/\tau_{j}+|t_{j}-t_{j}^{\prime }{|}^{2}/3\tau_{j}^{2}\right)\exp\;\left(-|t_{j}-t_{j}^{\prime }|/\tau_{j}\right).$$

This kernel satisfies the differentiability condition (7), where lengthscale parameters $\tau_{j}$s are chosen by the fivefold cross-validation. We use the actual output as the reference given by $f_{0}(S_{0},r_{*},\sigma_{*})=S_{0}\Phi (\;-d_{1}+\sigma_{*})-100e^{-r_{*}}\Phi (\;-d_{1})$ when $T=1,P_{0}=100$, where $d_{1}=\sigma_{*}^{-1}[\;\log\;100-\log\;(S_{0})-(r_{*}-\sigma_{*}^{2}/2)]$ and Φ(⋅) is the CDF of standard normal distribution. We estimate the $\text{MSE}=E({\hat{\;f}}_{n}-f_{0}{)}^{2}$ by a Monte Carlo sample of $10^{4}$ test points in [80,120]×[0.01,0.05]×[0.2,1].

Figure 1 reports the MSEs for different methods: stochastic kriging with only function data (i.e. p=0), our estimator with different types of gradient data. The results are averaged over 1,000 simulations in each setting. It is seen that our estimator with gradient data gives a substantial improvement in estimation compared to stochastic kriging without gradient. For example, the MSE of $n=7^{3},q=1,000$ with full gradient (i.e. p=3) is comparable to the MSE of $n=14^{3},q=1,000$ without gradient (i.e. p=0). Since it needs little additional cost to estimate gradients by (18), our estimator essentially saves the computational cost of sampling at new designs. It is also seen that a faster convergence rate is obtained when incorporating all gradient data (i.e. p=3) compared to p≤2. This confirms our theoretical finding in Section 3.3.

Table 1 reports the ratios of the MSE of our estimator with two types of gradient data (i.e. p=2) relative to the MSE of stochastic kriging with only function data (i.e. p=0). It is seen that incorporating gradient data leads to a faster convergence rate, which also agrees with our finding in Section 3.3.

**Table 1.** The ratios of mean-squared error (MSE) with two types of gradient data (i.e. p=2) relative to MSE with only function data (i.e. p=0), for the example in Section 4.1

*n*	q=1,000	q=2,000	q=5,000
$7^{3}=343$	0.6818	0.6789	0.6612
$14^{3}=2,744$	0.5850	0.5848	0.5835
$21^{3}=9,261$	0.5484	0.5483	0.5294

### 4.2 Cost estimation in economics

We consider an economic problem of the cost function estimation. Write the cost function $f_{0}(t)=f_{0}(t_{1},\ldots ,t_{d})$, where $t_{d}$ denotes the level of output and $\left(t_{1},\ldots ,t_{d-1}\right)$ represent the prices of d−1 factor inputs. The Cobb–Douglas production function (Varian, 1992) yields that


$$f_{0}(t_{1},\ldots ,t_{d})=c_{0}^{-\frac{1}{c}}\prod_{1\leq j\leq d-1}{\left(\frac{c}{c_{j}}\right)}^{\frac{c_{j}}{c}}\prod_{1\leq j\leq d-1}t_{j}^{\frac{c_{j}\;\;}{c}}t_{d}^{\frac{1}{\;\;c}}.$$

Here $c_{0}$ is the efficiency parameter, $c_{1},\ldots ,c_{d-1}$ are elasticity parameters, and $c=c_{1}+\cdots +c_{d-1}$. Our goal is to estimate the cost function $f_{0}(t)$. The function data of $f_{0}(t)$ are observed at design $t^{\left(0\right)}\in X^{d}$. The gradient data of $f_{0}(t)$ with respect to input prices are the quantities of factor inputs that are also observable (Hall & Yatchew, 2007),


$$Y^{\left(j\right)}=\frac{\partial }{\partial t_{j}}f_{0}(t^{\left(j\right)})+\epsilon^{\left(j\right)},\;{\;t}^{\left(j\right)}\in X^{d},\;j=1,\ldots ,d-1.$$

Here $t^{\left(j\right)}=t^{\left(0\right)}\in X^{d}$ for 1≤j≤d−1 that typically follows a random design. Moreover, the observational errors are usually assumed to be i.i.d. (Hall & Yatchew, 2007) and hence satisfy the error structure (3). Since the gradient data about $\partial f_{0}/\partial t_{d}$ is not usually observable, it motivates our modelling of p∈{1,…,d} in model (2). Clearly, $f_{0}(t)$ in this example follows the SS-ANOVA model (4). In the experiment, we consider d=3 and fix $t_{3}=1$ since the cost function is homogeneous of degree one in $\left(t_{1},t_{2},t_{3}\right)$, that is $f_{0}(t_{1},t_{2},t_{3},t_{4})=t_{3}f_{0}(t_{1}/t_{3},t_{2}/t_{3},1,t_{4})$. The data are generated through,


$$Y^{\left(0\right)}=f_{0}(t_{1},t_{2},1,t_{4})+\epsilon^{\left(0\right)},\;Y^{\left(j\right)}=\frac{\partial f_{0}(t_{1},t_{2},1,t_{4})}{\partial t_{j}}+\epsilon^{\left(j\right)}\;\text{ for}\;j=1,2,$$

where $c_{0}=1,c_{1}=0.8,c_{2}=0.7,c_{3}=0.6$, and the designs $t^{\left(j\right)},j=0,1,2$ follow the i.i.d. uniform distribution in $[0.5,1.5{]}^{3}$. Suppose that $\epsilon^{\left(j\right)},j=0,1,2$ are Gaussian with zero means, standard deviations 0.35, and correlation *ρ*. We consider varying sample size n=100,200,500,1,000, the correlation ρ=0,0.4,0.9, and set the number of random feature s=n/10 for constructing the random feature estimator in (13).

####  

##### Comparison with existing method

Hall and Yatchew (2007) proposed a regression-kernel method for incorporating gradient for cost function estimation. We compare the performance of our estimator (13) with that of Hall and Yatchew’s estimator. For the estimator in Hall and Yatchew (2007), we follow Hall and Yatchew’s Example 3 to use the tensor product Matérn kernel (19) to average $\left(t_{1},t_{4}\right)$ and $\left(t_{2},t_{4}\right)$ directions locally, and then average the estimates. The MSE is estimated by a Monte Carlo sample of $10^{4}$ test points in $[0.5,1.5{]}^{3}$.

Table 2 reports the MSEs and standard errors for varying sample size *n*, correlation *ρ*, and different methods: our estimator with only function data (i.e. p=0), Hall and Yatchew’s estimator with function and gradient data (i.e. p=2), our estimator with function and gradient data (i.e. p=2). The results are obtained over 1,000 simulations in each setting. It is seen that MSEs and standard errors of incorporating gradient information are significantly smaller than that without gradient. Moreover, the performances of our estimator compare favourably with that of Hall and Yatchew’s estimator.

**Table 2.** The comparison of average mean-squared errors and standard errors of our estimator with those of Hall and Yatchew’s estimator, considering various gradient types, for the example in Section 4.2 with 1,000 simulations

		Our estimator (13)	Hall and Yatchew (2007)	Our estimator (13)
		with only $Y^{\left(0\right)}$	with $Y^{\left(0\right)}+Y^{\left(1\right)}+Y^{\left(2\right)}$	with $Y^{\left(0\right)}+Y^{\left(1\right)}+Y^{\left(2\right)}$
	ρ=0	127.1471(22.8495)	61.4098(17.4460)	47.4739(13.5196)
n=100	ρ=0.4	128.9210(23.3594)	63.1006(17.9422)	49.8963(13.6218)
	ρ=0.9	129.6300(24.8577)	64.5989(19.8965)	51.9224(13.6433)
	ρ=0	76.6199(15.9333)	33.3001(11.5872)	24.1501(8.2730)
n=200	ρ=0.4	77.7602(16.1079)	35.0696(11.7615)	25.5342(8.3062)
	ρ=0.9	77.9138(16.3593)	36.2591(11.9210)	27.0137(8.6223)
	ρ=0	36.1925(8.0550)	16.3861(5.5399)	9.3499(2.5570)
n=500	ρ=0.4	38.0683(8.2180)	18.2355(5.6164)	10.4708(2.5619)
	ρ=0.9	38.9311(8.3654)	18.7698(5.6877)	11.0498(2.6124)
	ρ=0	21.8570(5.6051)	9.2788(2.2411)	4.5364(1.6147)
n=1,000	ρ=0.4	22.4943(5.6312)	10.4801(2.2433)	5.1468(1.6561)
	ρ=0.9	22.9499(5.6446)	10.6193(2.3386)	5.3288(1.8550)

*Note*. The table shows metrics: ‘average mean-squared error (standard error)’, in units of $10^{-4}$.

Table 3 reports the ratios of the MSE of our estimator incorporating two types of gradient data (i.e. p=2) relative to the MSE of Hall and Yatchew’s estimator incorporating two types of gradient data (i.e. p=2). It is seen that the ratio decreases with the sample size, which agrees with our theoretical finding in Section 3.3, since our estimator in this example converges at the rate $n^{-2m/(2m+1)}$ by Theorem 3, and Hall and Yatchew’s estimator converges at a slower rate $n^{-m/(m+1)}$.

**Table 3.** The ratios of mean-squared error (MSE) of our estimator with two types of gradient data (i.e. p=2) relative to MSE of Hall and Yatchew’s estimator with two types of gradient data (i.e. p=2), for the example in Section 4.2

	ρ=0	ρ=0.4	ρ=0.9
n=100	0.7731	0.7907	0.8038
n=200	0.7252	0.7281	0.7450
n=500	0.5706	0.5742	0.5887
n=1,000	0.4889	0.4911	0.5018

Tables 2 and 3 also indicate that s=n/10 yields sufficient accuracy for the estimations by the random feature estimator in (13). Therefore, in practical applications, an *s* significantly smaller than the theoretical minimum of s=O(nlogn) in Theorem 3 might often suffice.

### 4.3 Ion channel experiment

We consider a real data example from a single voltage clamp experiment. The experiment is on the sodium ion channel of the cardiac cell membranes. The experiment output $z_{k}$ measures the normalized current for maintaining a fixed membrane potential of −35mV and the input $x_{k}$ is the logarithm of time. The sample size of the ion channel experiment is N=19. Computer model has been used to study the ion channel experiment (Plumlee, 2017). Let η(x,t) be the computer model that approximates the physical system for the ion channel experiment, where *x* is the experiment input and $t\in X^{d}$ is the calibration parameter whose value are unobservable. For analysing the ion channel experiment, the computer model is given by $\eta (x,t)=e_{1}^{⊤}\exp\;(\;\exp\;(x)A(t))e_{4}$, where $t=(t_{1},t_{2},t_{3}{)}^{⊤}\in X^{d}$, d=3, $e_{1}=(1,0,0,0{)}^{⊤},e_{4}=(0,0,0,1{)}^{⊤}$, and


$$A(\;t)=\left(\begin{array}{cccc} -t_{2}-t_{3} & t_{1} & 0 & 0\\ t_{2} & -t_{1}-t_{2} & t_{1} & 0\\ 0 & t_{2} & -t_{1}-t_{2} & t_{1}\\ 0 & 0 & t_{2} & -t_{1} \end{array}\right).$$

Our goal is to estimate the function, $f_{0}(t)=E_{\left(x,z\right)}[z-\eta (x,t){]}^{2}$, which is useful for visualization, calibration, and understanding how well the computer model approximates the physical system in this experiment (Kennedy & O’Hagan, 2001). The function data at design $t^{\left(0\right)}\in X^{3}$ is generated by,


$$Y^{\left(0\right)}=\frac{1}{N}\sum_{k=1}^{N}[z_{k}-\eta (x_{k},{\;t}^{\left(0\right)}){]}^{2},\;\text{where}\;N=19.$$

The gradient of computer model, i.e. $\nabla_{t}\eta (x,t)$, can be obtained using the chain rule-based automatic differentiation. By the cheap gradient principle (Griewank & Walther, 2008), the cost for computing $\nabla_{t}\eta (x,t)$ is at most four or five times the cost for function evaluation η(x,t) and hence, the gradient is cheap to obtain. Then the estimator for the gradient of $f_{0}(t)$ is given by,


$$Y^{\left(j\right)}=-\frac{2}{N}\sum_{k=1}^{N}\left[z_{k}-\eta (x_{k},{\;t}^{\left(j\right)})\right]\frac{\partial }{\partial t_{j}}\eta (x_{k},{\;t}^{\left(j\right)}),\;{\;t}^{\left(j\right)}\in X^{3},\;j=1,2,3.$$

In the experiment, we choose i.i.d. uniform designs for $t^{\left(j\right)}$s, j=0,1,2,3 from $X^{3}$ with the sample size n=1,000,2,000,3,000,5,000.

####  

##### Comparison with existing method

Morris et al. (1993) proposed a stationary Gaussian process method to incorporate gradient data for estimation. We compare the performance of our estimator (13) with that of Morris et al.’s estimator. We use the Matérn kernel (19) for both our estimator and Morris et al.’s estimator, and estimate the MSE by a Monte Carlo sample of $10^{4}$ test points in $X^{3}$. We set the number of random feature s=n/10 for constructing the estimator (13). Since the true function $f_{0}(t)$ is unknown at each test point, we approximate it by using total N=19 real ion channel samples at each test point. The function and gradient training data are generated using $N^{\prime }=10$ real ion channel samples, which are randomly chosen from the total N=19 samples.

Table 4 reports the MSEs and standard errors for varying sample size *n* and different methods: our estimator with only function data (i.e. p=0), Morris et al.’s estimator with function and gradient data (i.e. p=3), our estimator with function and gradient data (i.e. p=3). The results are obtained over 1,000 simulations in each setting. It is evident that the gradient data can significantly improve the estimation performance, and our estimator outperforms Morris et al.’s estimator.

**Table 4.** The comparison of average mean-squared errors and standard errors of our estimator with those of Morris et al.’s estimator, considering various gradient types, for the example in Section 4.3 with 1,000 simulations

	Our estimator	Morris et al. (1993)	Our estimator
	with only $Y^{\left(0\right)}$	with $Y^{\left(0\right)}+\cdots +Y^{\left(3\right)}$	with $Y^{\left(0\right)}+\cdots +Y^{\left(3\right)}$
n=1,000	10.6491(4.9867)	8.8956(4.8729)	7.7804(3.6737)
n=2,000	8.5302(4.3339)	6.5494(4.0728)	5.1375(2.4687)
n=3,000	6.4296(3.9595)	4.1940(3.2242)	3.1035(1.7187)
n=5,000	5.4143(3.2268)	3.0910(1.9073)	2.1305(0.9322)

*Note*. The table shows metrics: ‘average mean-squared error (standard error)’, in units of $10^{-6}$.

## 5 Related work

We review related work from multiple fields of literature, including nonparametric regression, function interpolation, and dynamical systems.

There is a growing body of literature on nonparametric regression with derivatives. Our work relates to the pioneering work of Hall and Yatchew (2007, 2010), which established the root-*n* consistency for nonparametric estimation given mixed and sufficiently *higher-order* derivatives. However, obtaining higher-order derivatives in practice, such as in economics and stochastic simulation, is challenging. In contrast, we focus on gradient information, which comprises *first-order* derivatives that are easier to obtain in practice. We demonstrate that the minimax optimal rates for estimating SS-ANOVA models are accelerated by using gradient data. In particular, we show that SS-ANOVA models are immune to the curse of interaction when gradient information is utilized.

Function interpolation with gradients has been widely studied. For exact data and one-dimensional functions, Karlin (1969) and Wahba (1971) showed that at certain deterministic designs for data without gradients, incorporating gradients into the dataset provides no new information for function interpolation. However, this result cannot be extended to the case of noisy data. Morris et al. (1993) incorporated noiseless derivatives for deterministic surface estimation in computer experiments. Unlike these works, we consider noisy gradient information for nonparametric estimation.

Our work is also related to the literature on dynamical systems and stochastic simulation. Solak et al. (2002) considered the identified linearization around an equilibrium point for estimating derivatives in nonlinear dynamical systems, using Gaussian processes for a combination of function and derivative observations. Chen et al. (2013) used stochastic kriging to incorporate gradient estimators and improve surface estimation, where stochastic kriging (Ankenman et al., 2010) is a metamodelling technique for representing the mean response surface implied by a stochastic simulation. However, the rates of convergence are not studied in the works by Solak et al. (2002) and Chen et al. (2013). We quantify the improved rates of convergence in nonparametric estimation by using gradient data.

## 6 Conclusion

Statistical modelling of gradient information has become an increasingly important issue in many areas of science and engineering. We have developed an approach based on partial derivatives, either observed or estimated, to effectively estimate nonparametric functions. This proposed approach and computational algorithm could lead to methods useful for practitioners. Our theoretical results demonstrate that SS-ANOVA models are immune to the *curse of interaction* when using gradient information. Moreover, for additive models, the rates using gradient information achieve the *parametric rate* of root-*n*.

As a working model, we assume that the eigenvalues decay at the same polynomial rate across component RKHSs, $H^{j}$, which is characteristic of Sobolev kernels, among other commonly used kernels. It is of interest to consider incorporating gradient information in more general settings, for example, when eigenvalues decay at different rates, or if the eigenvalues for some components decay exponentially. These directions will be left for future studies.

## Supplementary Material

qkae093_Supplementary_Data

## Data Availability

The ion channel experiment data is available at https://github.com/XiaowuDai/gradient.
