# Supplementary material for: Nonparametric estimation via partial derivatives
Source: J R Stat Soc Series B Stat Methodol. 2024 Sep 11;87(2):319–36. doi: 10.1093/jrsssb/qkae093 (PMC11985098; doi:10.1093/jrsssb/qkae093)
Supplement: qkae093_Supplementary_Data [file qkae093_supplementary_data.pdf]

Supplementary Appendix for Nonparametric Estimation via Partial Derivatives

Xiaowu Dai

University of California, Los Angeles

S1 Optimal Rates Under Deterministic Designs

We present the minimax optimal rates under deterministic designs. Specifically, we consider the regular lattice design, which is also called the tensor product design. A regular lattice of size $n = l_1 \times \cdots \times l_d$ on $\mathcal{X}^d$ is a collection of design points $\{\mathbf{t}_1, \ldots, \mathbf{t}_n\} = \{(t_{i_1,1}, t_{i_2,2}, \ldots, t_{i_d,d}) \mid i_j = 1, \ldots, l_j, j = 1, \ldots, d\}$, where $t_{i,j} = i/l_j$, $i = 1, \ldots, l_j, j = 1, \ldots, d$. This design is widely used for SS-ANOVA models (Wahba et al., 1995; Lin, 2000). Under regular lattices, it is without loss of generality to assume that $f_0 : \mathcal{X}^d \mapsto \mathbb{R}$ has a periodic boundary condition. This is because any finite sequence $\{f(\mathbf{t}_1), \ldots, f(\mathbf{t}_n)\}$ can be associated with a periodic sequence,

$$
\begin{aligned}
&f^{\text{per}}\left(i_1/l_1, \cdots, i_d/l_d\right) \\
&= \sum_{q_1=-\infty}^{\infty} \cdots \sum_{q_d=-\infty}^{\infty} f\left(i_1/l_1 - q_1, \cdots, i_d/l_d - q_d\right), \quad \forall (i_1, \ldots, i_d) \in \mathbb{Z}^d,
\end{aligned}
$$

where $\mathbb{Z}$ is the set of integers, and let $f(\cdot) \equiv 0$ outside and on the unobserved boundaries of $\mathcal{X}^d$. On the other hand, any finite sequence $\{f(\mathbf{t}_1), \ldots, f(\mathbf{t}_n)\}$ can be recovered from periodic sequence $f^{\text{per}}(\cdot)$. We now present the main results under deterministic design by first stating a minimax lower bound.

Theorem S1. *Assume that $\lambda_\nu \asymp \nu^{-2m}$ for some $m > 3/2$. Under the regression models* (1) *and* (2) *where f_0 follows the SS-ANOVA model* (4) *and the designs $\mathbf{t}^{(0)}$ and $\mathbf{t}^{(j)}$s are from the regular lattice. Then under the error structure (3), there exists a constant c that does*

not depend on n such that

$$\liminf_{n\to\infty} \inf_{\tilde{f}} \sup_{f_0\in\mathcal{H}} \mathbb{E}\int_{\mathcal{X}^d} \left[\tilde{f}(\mathbf{t}) - f_0(\mathbf{t})\right]^2 d\mathbf{t} \geq \begin{cases} c\left[n(\log n)^{1-(d-p)\wedge r}\right]^{-\frac{2m}{2m+1}}, & \text{if } 0 \leq p < d, \\ c\left[n^{-\frac{2mr}{(2m+1)r-2}}\mathbb{1}_{r\geq 3} + n^{-1}(\log n)^{r-1}\mathbb{1}_{r<3}\right], & \text{if } p = d, \end{cases}$$

where the infimum of $\tilde{f}$ is taken over all measurable functions of the data.

The lower bound is established via the analysis of a version of the hardest rectangular subproblem. See, e.g., Donoho et al. (1990). We relegate its proof to Section S5. Next, we show that the rates given in the lower bound in Theorem S1 is attainable by the estimator $\widehat{f}_n$ in (9). Hence $\widehat{f}_n$ is also minimax rate optimal under deterministic design.

Theorem S2. *Assume that $\lambda_\nu \asymp \nu^{-2m}$ for some $m > 3/2$. Under the regression models* (1) *and* (2) *where f_0 follows the SS-ANOVA model* (4) *and the designs $\mathbf{t}^{(0)}$ and $\mathbf{t}^{(j)}$s are from the regular lattice. Then under the error structure (3), there exists a constant C that does not depend on n such that the estimator $\widehat{f}_n$ defined by (9) satisfies*

$$\limsup_{n\to\infty} \sup_{f_0\in\mathcal{H}} \mathbb{E}\int_{\mathcal{X}^d} \left[\widehat{f}_n(\mathbf{t}) - f_0(\mathbf{t})\right]^2 d\mathbf{t} \leq \begin{cases} C\left[n(\log n)^{1-(d-p)\wedge r}\right]^{-\frac{2m}{2m+1}}, & \text{if } 0 \leq p < d, \\ C\left[n^{-\frac{2mr}{(2m+1)r-2}}\mathbb{1}_{r\geq 3} + n^{-1}(\log n)^{r-1}\mathbb{1}_{r<3}\right], & \text{if } p = d. \end{cases}$$

Here the tuning parameter λ in (9) is chosen by $\lambda \asymp \left[n(\log n)^{1-(d-p)\wedge r}\right]^{-2m/(2m+1)}$ when $0 \leq p < d$, and $\lambda \asymp n^{-(2mr-2)/[(2m+1)r-2]}$ when $p = d, r \geq 3$, and $\lambda \asymp (n\log n)^{-(2m-1)/2m}$ when $p = d, r = 2$, and $\lambda \asymp n^{-(m-1)/m}$ when $p = d$, $r = 1$.

The proof of Theorem S2 is also presented in Section S5. Theorems S1 and S2 together imply that under deterministic design, the minimax optimal rate for estimating $f_0 \in \mathcal{H}$ with partial derivatives is

$$\left[n(\log n)^{1-(d-p)\wedge r}\right]^{-\frac{2m}{2m+1}} \mathbb{1}_{0\leq p<d} + \left[n^{-\frac{2mr}{(2m+1)r-2}}\mathbb{1}_{r\geq 3} + n^{-1}(\log n)^{r-1}\mathbb{1}_{r<3}\right]\mathbb{1}_{p=d}.$$

This result coincides with the rate given by (15) under random design. Different from ours, Hall and Yatchew (2010) proposed a series-type estimator for incorporating various derivative data under the regular lattice. Hall and Yatchew (2010) showed that their estimator

achieves the $\sqrt{n}$-consistency when sufficiently *high-order* derivatives are available. However, it is difficult to obtain high-order derivative data in practice, such as in economics and stochastic simulation. In contrast, we focus on incorporating *first-order* partial derivatives that are easier to obtain in practice. Chen et al. (2013) studied a stochastic kriging method for incorporating partial derivatives, and analyzed its estimation error under certain widely spread designs, where the spatial correlations of observational errors at distinct design points approximately vanish. However, rates of convergence are not studied in Chen et al. (2013). By contrast, we quantify the improved rates of convergence with partial derivatives, which result holds under the general error structure (3).

S2 Error structures of common gradient estimators

We give three examples to illustrate that the random error assumption in (3) holds for gradient estimators that are commonly used in real-world settings.

Example 1: Infinitesimal perturbation analysis (IPA). In Section 4.1, we studied the example of call option pricing with stochastic simulations, where the unbiased gradient estimators are derived using IPA. Generally, IPA estimators are obtained under the condition (see, Ankenman et al., 2010; Chen et al., 2013) that common random numbers are not used across design points. Then, correlation exists only within the error terms $(\epsilon_i^{(0)}, \epsilon_i^{(1)}, \ldots, \epsilon_i^{(p)})^\top$ for the same design point i and not between those of different design points, $\mathrm{Cov}[\epsilon_i^{(j)}, \epsilon_{i'}^{(j')}] = 0$, where $i \neq i'$ and $j, j' = 0, 1, \ldots, p$. Therefore, the errors of IPA gradient estimators satisfy the error assumption (3).

Moreover, define the correlation between the simulation noise in the response and in the estimator of the rth gradient component as $\rho_i^{(0,j)} = \mathrm{Corr}[\epsilon_i^{(0)}, \epsilon_i^{(j)}], j = 1, \ldots, p$. Let the correlation between the simulation noise in the estimators of a pair of distinct gradient components be $\rho_i^{(j_1,j_2)} = \mathrm{Corr}[\epsilon_i^{(j_1)}, \xi_i^{(j_2)}], j_1, j_2, = 1, \ldots, p$ and $j_1 \neq j_2$. Notably, our error assumption (3) accommodates the scenario where the correlations $\rho_i^{(0,j)}$ and $\rho_i^{(j_1,j_2)}$ at different design points are not necessarily equal. This characteristic is consistent with the properties of the IPA estimators as shown in Ankenman et al. (2010) and Chen et al. (2013).

Example 2: Observational gradients. In Section 4.2, we considered the example of cost estimation in economics, where the gradient data are directly observable. More specifically, the partial derivatives of $f_0(\mathbf{t})$ with respect to input prices correspond to observable

quantities of factor inputs.

In such observational studies where derivative data are available, the errors are commonly assumed to be i.i.d. (Hall and Yatchew, 2007). Then, $\mathrm{Cov}[\epsilon_i^{(j)}, \epsilon_{i'}^{(j')}] = 0$, where $i \neq i'$ and $j, j' = 0, 1, \ldots, p$. Therefore, the errors of observational gradients satisfy the error assumption (3).

Example 3: Finite difference method. We explore the finite difference method as an alternative approach to derivative estimation, as applied in the life table estimation example in Appendix S3.2. Specifically, we consider the finite-difference gradient estimator at $t_i^{(0)} \in \mathbb{R}$ for $i = 1, \ldots, n-1$,

$$\widehat{\frac{df_0}{dt}}(t_i^{(0)}) \equiv \frac{y_{i+1}^{(0)} - y_i^{(0)}}{t_{i+1}^{(0)} - t_i^{(0)}} = \frac{f(t_{i+1}^{(0)}) - f(t_i^{(0)})}{t_{i+1}^{(0)} - t_i^{(0)}} + \frac{\epsilon_{i+1}^{(0)} - \epsilon_i^{(0)}}{t_{i+1}^{(0)} - t_i^{(0)}}$$
$$= f'(t_i^{(0)}) + \underbrace{\left(\frac{f(t_{i+1}^{(0)}) - f(t_i^{(0)})}{t_{i+1}^{(0)} - t_i^{(0)}} - f'(t_i^{(0)}) \right)}_{\text{term I}} + \underbrace{\frac{\epsilon_{i+1}^{(0)} - \epsilon_i^{(0)}}{t_{i+1}^{(0)} - t_i^{(0)}}}_{\text{term II}}.$$

By the Taylor expansion, we have

$$\text{term I} = \frac{1}{2} f''(\tilde{t})(t_{i+1}^{(0)} - t_i^{(0)}),$$

where $\tilde{t}$ lies between $t_i^{(0)}$ and $t_{i+1}^{(0)}$. Assuming that the observation errors $\epsilon_i^{(0)}$s of function data are i.i.d. and centered, and considering the continuity of the second-order derivative of f along with $|t_{i+1}^{(0)} - t_i^{(0)}| = o(n^{-1/2})$, the bias of the finite-difference gradient estimator satisfies,

$$\mathbb{E}[\epsilon_i^{(1)}] = \mathbb{E}[\text{term I}] + \mathbb{E}[\text{term II}] = \frac{1}{2} f''(\tilde{t})(t_{i+1}^{(0)} - t_i^{(0)}) = o(n^{-1/2}).$$

Note that the assumption $|t_{i+1}^{(0)} - t_i^{(0)}| = o(n^{-1/2})$ is mild and typically satisfied in practical settings, such as when $t_i^{(0)}$'s are equally spaced in $\mathcal{X} = [0, 1]$, where $|t_{i+1}^{(0)} - t_i^{(0)}| = 1/n = o(n^{-1/2})$. Moreover, for $|i - i'| > 1$, we have $\mathrm{Cov}[\epsilon_i^{(0)}, \epsilon_{i'}^{(1)}] = 0$ and $\mathrm{Cov}[\epsilon_i^{(1)}, \epsilon_{i'}^{(1)}] = 0$. Hence, the covariance of the finite-difference gradient estimator satisfies,

$$\mathrm{Cov}[\epsilon_i^{(j)}, \epsilon_{i'}^{(j')}] = O(|i - i'|^{-2}),$$

where $i \neq i'$ and $j, j' = 0, 1$. Therefore, the errors of finite-difference gradient estimators satisfy the error assumption (3).

S3 Additional Numerical Examples

In this section, we provide additional numerical examples. We study a manufacturing example in Section S3.1, analyze a real dataset on an actuarial life table in Section S3.2, and explore a statistical inference example on cost estimation in Section S3.3.

S3.1 Flexible assembly systems in manufacturing

We study a stochastic simulation in manufacturing that generates partial derivatives. Closed-loop flexible assembly system (CLFAS) is a useful tool to lower production costs and increase flexibility in manufacturing (Suri and Leung, 1987; Chen et al., 2013).

Since building a CLFAS is expensive, it is important to provide a fast and accurate prediction to the CLFAS performance. We consider a CLFAS of six automatic workstations and a conveyor with six pallets shown in Figure S1. Note that our analysis can be extended to any number of workstations or pallets. In this CLFAS, unfinished parts are loaded and unloaded through workstation 1 and proceed on the pallets. The operation time at each workstation j, $1 \leq j \leq 6$, is given by $t_j + \mathbf{1}\{\text{jam at station } j\}R_j$, where t_j is the fixed machine time (in minutes) and R_j is the additional random time (in minutes) to clear the machine j if it jams. Let p_j be the probability of a part causing a jam at workstation j. Since the operation time is random, queueing may occur in the system. Our goal is to estimate $f_0(t_1, \ldots, t_6)$, which denotes the expected production time of the first 5000 parts completed by the CLFAS. Here f_0 can be approximated by a SS-ANOVA model in (4) because if there is no queue occurs, f_0 has an additive structure in the covariates $(t_1, \ldots, t_6)$. In the experiment, we fix $p_j = 0.5\%$ and let R_j i.i.d. uniformly sample from $[0.1, 1.1]$. The design points of $(t_1, \ldots, t_6)$ are uniformly random in $[3, 9]^6$ with the sample size $n = 100$. To address the impact of stochastic simulation noise, we simulate 1000 stochastic simulations of CLFAS at each design and then average the results.
Suri and Leung (1987) proposed an IPA derivative estimators for a CLFAS as follows.

Step 1: Let $\mathcal{A}_{j_1,j_2}$s be accumulator variables. Initialize: $\mathcal{A}_{j_1,j_2} = 0$ for $j_1, j_2 = 1, \ldots, 6$;

Step 2: At the end of an operation at station j, let $\mathcal{A}_{j,j} \leftarrow \mathcal{A}_{j,j} + 1$, $j = 1, \ldots, 6$;

Step 3: If a pallet leaving station j_1 going to station j_1' terminates an idle period of station j_1', let $\mathcal{A}_{j_1',j_2} \leftarrow \mathcal{A}_{j_1,j_2}$, $j_2 = 1, \ldots, 6$;

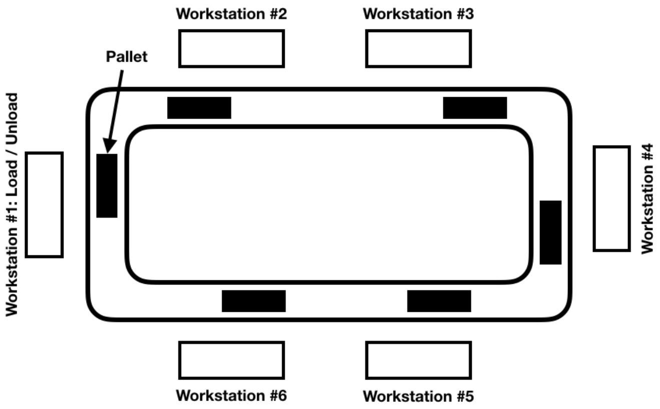

Figure S1: Diagram of CLFAS for the example in Section S3.1.

Step 4: If a pallet leaving station j_1 going to station j_1' terminates a blocked period of station j_1, let $\mathcal{A}_{j_1,j_2} \leftarrow \mathcal{A}_{j_1',j_2}$, $j_2 = 1, \ldots, 6$;

Step 5: At the end of the simulation, let P be the total number of parts completed and L be the full length of simulation in minutes. Output the function data $Y^{(0)}(\mathbf{t}) = L/P$ and the IPA derivative estimator $Y^{(j)}(\mathbf{t}) = \mathcal{A}_{6,j}/P$ for $j = 1, \ldots, 6$.

In the data generating process, the correlation only exists for function and derivative data at the same design, not data across different design points. Hence the random errors satisfy the error structure in (3). In this example, obtaining function data at a new design requires to conduct 1000 new simulation replications. However, it only needs to record a small matrix $\{\mathcal{A}_{j_1,j_2}\}_{j_1,j_2=1}^{6}$ in the algorithm of Suri and Leung (1987) for obtaining the IPA derivative estimators, whose computational cost is negligible compared to that of obtaining a new function data.

Comparison to existing method. We compare our estimator (13) and the stochastic kriging method (Ankenman et al., 2010). We use the 6-dimensional version of the tensor product Matérn kernel (19), and choose lengthscale parameters by the five-fold cross-validation. We estimate the MSE of estimation by a Monte Carlo sample of 10^4 test points in $[3, 9]^6$. Since the true production time is unknown at each test point, we approximate it by replicating 10^6 CLFAS experiments at each test point.

Figure S2 reports the MSEs for different methods: stochastic kriging with only function data (i.e., $p = 0$), and our estimator with derivative data (i.e., $p = 6$). The results are

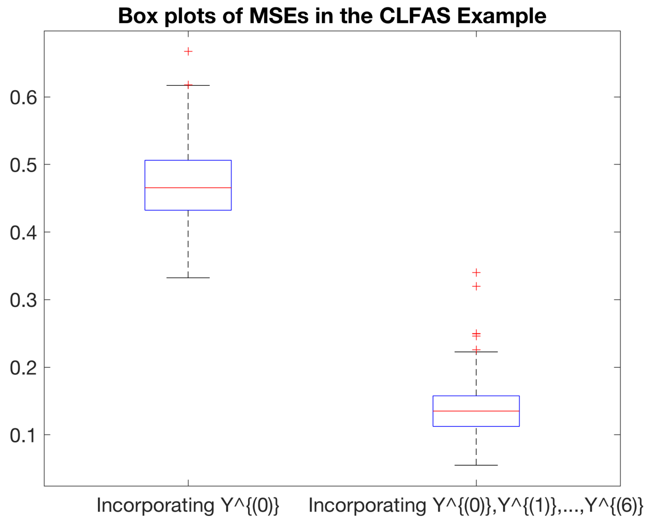

Figure S2: The box plots of MSEs of our estimator with derivative data and the stochastic kriging without derivative data, for the example in Section S3.1.

averaged over 1000 simulations. It is seen that incorporating partial derivatives leads to a significant improvement of estimation compared to without using the derivatives.

S3.2 Life table estimation

We study a real data of U.S. 2015 period life table for the social security area (`www.ssa.gov/OACT/STATS/table4c6.html#fn2`), where the data separate the male and female population. The life table in actuarial science provides probabilities of survival and death at integer ages (Frees and Valdez, 1998). To value payments that are not at integer ages, actuaries need to make a fractional age assumption of surviving at fractional ages. Our goal is to estimate the survival distribution function $f_0(t)$. Let $u(t)$ be the force of mortality function. It is known that (see, Frees and Valdez, 1998),

$$f_0'(t) = -f_0(t)u(t). \tag{S1}$$

The function data $Y^{(0)}$ on $f_0(t)$ are generated using the death probability from life table. The force of mortality function $u(t)$ can be estimated using the number of people that survive at age t, where the detail is given as follows. Denote by $l(t)$ the number of people that survive at age t. Then a divided-difference estimator for $u(t)$ is (Jones and Mereu, 2002),

$$u(0) = \frac{1}{2l(0)}[3l(0) - 4l(1) + l(2)], \quad u(t) = \frac{1}{2l(t)}[l(t-1) - l(t+1)] \text{ for } t > 0.$$

The function $Y^{(0)}$, together with the estimate of $u(t)$, yield the derivative $Y^{(1)}$ according to (S1). We choose the design t from equally spaced integers from $[0, 119]$ with the sample size $n = 5, 10, 15, 20$. The endpoints of $[0, 119]$ are included.

Table S1: The comparison of average MSEs and standard errors of our estimator with those of smoothing spline estimator, for the example in Section S3.2 with 1000 simulations. The table shows metrics: "average MSE (standard error)," in units of 10^{-4}.

		$n = 5$	$n = 10$	$n = 15$	$n = 20$
M	Smoothing spline estimator with $Y^{(0)}$	15.3674 (4.8815)	6.7944 (2.2596)	1.7687 (0.6676)	0.1745 (0.0594)
	Our estimator with $Y^{(0)} + Y^{(1)}$	**7.4381 (2.5242)**	**1.6488 (0.5009)**	**0.3446 (0.1012)**	**0.0227 (0.0098)**
F	Smoothing spline estimator with $Y^{(0)}$	23.0655 (7.1699)	9.9948 (3.8025)	2.2299 (0.8110)	0.5925 (0.1569)
	Our estimator with $Y^{(0)} + Y^{(1)}$	**9.4745 (3.2385)**	**2.4790 (0.8654)**	**0.4091 (0.1015)**	**0.0755 (0.0152)**

Comparison to existing method. Smoothing spline (Wahba, 1990) is widely used for smoothing noisy data. We compare the results of our estimator (13) using the estimated derivative and the smoothing spline without using the derivative. We use the Matérn kernel (19) and estimate the MSE by using the full sample at $t = 0, 1, \cdots, 119$.

Table S1 reports the MSEs and standard errors for varying sample size n, different population, and different methods: smoothing spline with only function data (i.e., $p = 0$), and our estimator with function and derivative data (i.e., $p = 1$). The results are obtained over 1000 simulations in each setting. It is seen that our estimator incorporating derivative data significantly improves the estimation results compared to the smoothing splines.

Table S2 reports the ratios of the MSE of our estimator incorporating derivative data (i.e., $p = 1$) relative to the MSE of smoothing spline estimator with only function data (i.e., $p = 0$). It is seen that the ratio decreases with the sample size, which agrees with our theory in Section S1 that incorporating derivative data accelerates the convergence rate.

Table S2: The ratios of MSE of our estimator with derivative data (i.e., $p = 1$) relative to MSE of spline estimator with only function data (i.e., $p = 0$), for the example in Section S3.2.

	$n = 5$	$n = 10$	$n = 15$	$n = 20$
Male	0.4840	0.2426	0.1948	0.1301
Female	0.4108	0.2480	0.1835	0.1274

S3.3 Statistical inference for the cost estimation in economics

We consider the economic problem of the cost function estimation in Section 4.2. We employ the bootstrap method (see, e.g., Efron and Tibshirani, 1993) to quantify the uncertainty of our estimators (13) for this example. The process for generating a bootstrap sample includes the following steps: (a) Produce B bootstrap samples by resampling centered residuals; (b) Re-estimate the functions to obtain B bootstrap estimates of f_0, denoted as $\widehat{f}_b^*$ for $b = 1, \ldots, B$. From this, we can derive a bootstrap confidence interval for f_0 at any new input $\mathbf{t}_{\text{new}}$. Specifically, we determine the $\alpha/2$ and $1 - \alpha/2$ sample quantiles from $\{\widehat{f}_1^*(\mathbf{t}_{\text{new}}), \ldots, \widehat{f}_B^*(\mathbf{t}_{\text{new}})\}$, represented as $z_{\alpha/2}^*$ and $z_{1-\alpha/2}^*$, respectively. The confidence interval is thus$(z_{\alpha/2}^*, z_{1-\alpha/2}^*)$. Given that bias in non-parametric regression may affect the asymptotic coverage of bootstrap confidence intervals, two common correction strategies include undersmoothing and oversmoothing (see, e.g., Härdle and Bowman, 1988; Hall, 1992a,b). Undersmoothing is often preferred due to its simplicity and effectiveness (Hall, 1992a). Our estimation procedure can be easily modified to incorporate undersmoothing by selecting a smaller smoothing parameter. Despite the potential for a modest gain in practical performance, these strategies require another ad hoc choice of the amount of undersmoothing or oversmoothing. Moreover, it is quite common to ignore this bias issue, essentially leading to the use of non-adjusted confidence intervals as suggested by Efron and Tibshirani (1993) and Ruppert et al. (2003). To keep the approach simple, we use the non-adjusted confidence intervals in this example with $B = 2000$. We set the significance level at 95%. The empirical coverage probability is calculated as the percentage of instances in which the confidence interval covers $f_0(\mathbf{t}_{\text{new}})$ across 1000 repetitions, with $\mathbf{t}_{\text{new}}$ randomly drawn from $\mathcal{X}^d$ for each repetition.

Table S3 compares the coverage probability and interval length when incorporating various levels of gradients ($p = 0, 1, 2$) using our method (13). The average length of the bootstrap confidence interval is computed across 1000 repetitions. We observe in Table S3 that the coverage probability of our estimator approximates 95% consistently across all gradient levels ($p = 0, 1, 2$). However, intervals without gradient information have larger lengths compared to those incorporating gradients. This observations aligns with our theoretical finding in Section 3.3 that the inclusion of gradient data results in a faster decease in the MSE of the estimator compared to excluding gradient data.

Table S3: Coverage probability and length of 95% bootstrap confidence intervals, incorporating various levels of gradients ($p = 0, 1, 2$) using our method (13), for the example in Section S3.3 with 1000 simulations.

		with only $Y^{(0)}$		with $Y^{(0)} + Y^{(1)}$		with $Y^{(0)} + Y^{(1)} + Y^{(2)}$	
		Prob (%)	Length	Prob (%)	Length	Prob (%)	Length
$n = 100$	$\rho = 0$	95.9722	14.4226	95.9116	13.4315	**96.8295**	**11.5146**
	$\rho = 0.4$	94.4613	15.6566	96.1340	13.6916	**96.9722**	**12.3477**
	$\rho = 0.9$	94.1245	16.4833	94.1276	14.3109	**96.1200**	**13.4637**
$n = 200$	$\rho = 0$	96.3252	11.0673	96.6061	9.1801	**97.3076**	**8.8906**
	$\rho = 0.4$	95.7476	12.1215	96.5717	10.0875	**96.2182**	**9.7177**
	$\rho = 0.9$	94.5275	12.5909	95.2201	11.3109	**96.9119**	**10.4494**
$n = 500$	$\rho = 0$	95.6127	8.4226	95.0207	6.6719	**96.4846**	**5.7415**
	$\rho = 0.4$	95.9650	8.6566	96.9369	7.4831	**95.8447**	**5.9061**
	$\rho = 0.9$	95.1417	9.4833	95.4791	7.8287	**95.4852**	**6.0834**
$n = 1000$	$\rho = 0$	95.9001	6.4732	96.2507	5.2168	**97.5913**	**3.6970**
	$\rho = 0.4$	95.3200	6.8322	95.3559	5.7529	**96.6146**	**3.8210**
	$\rho = 0.9$	95.0288	7.4983	95.9213	5.9815	**96.3667**	**4.1591**

S3.4 Additional comparisons with Hall and Yatchew's estimator

We present two additional examples to compare our estimator with the regression-kernel estimator in Hall and Yatchew (2007).

The first example is the stochastic simulation on call option pricing in Section 4.1. We adopt the same simulation setting, and use the actual output as the reference, which is given by $f_0(S_0, r_*, \sigma_*) = S_0\Phi\left(-d_1 + \sigma_*\right) - 100e^{-r_*}\Phi\left(-d_1\right)$. Here $d_1 = \sigma_*^{-1}[\log 100 - \log(S_0) - (r_* - \sigma_*^2/2)]$ and $\Phi(\cdot)$ is the CDF of standard normal distribution. For the estimator in Hall and Yatchew (2007), we follow the approach in Hall and Yatchew's Example 3 to average (S_0, r_*) and (S_0, σ_*) directions locally, and then average the estimates. The MSE$= \mathbb{E}(\widehat{f}_n - f_0)^2$ is estimated using a Monte Carlo sample of 10^4 test points in $[80, 120] \times [0.01, 0.05] \times [0.2, 1]$. Table S4 reports the MSEs and standard errors across varying sample size n, replications of the simulation q, and levels of gradient data. The results are summarized based on 1000 simulations for each scenario. It is seen that our estimator significantly enhances estimation accuracy compared to Hall and Yatchew's estimator.

The second example is the single voltage clamp experiment in Section 4.3. We follow the same simulation setting. For the estimator in Hall and Yatchew (2007), we again follow the approach in Hall and Yatchew's Example 3 to average (t_1, t_2) and (t_1, t_3) directions locally, and then average the estimates. The MSE$= \mathbb{E}(\widehat{f}_n - f_0)^2$ is estimated using a Monte Carlo

Table S4: The average MSEs and standard errors of our estimator and those of Hall and Yatchew's estimator, considering various gradient types, for the example in Section 4.1 with 1000 simulations. The table shows metrics: "average MSE (standard error)," in units of 10^{-2}.

n	q	Hall and Yatchew with $Y^{(0)}+Y^{(1)}+Y^{(2)}$	Our Estimator (13) with $Y^{(0)}+Y^{(1)}+Y^{(2)}$	Hall and Yatchew with $Y^{(0)}+Y^{(1)}+Y^{(2)}+Y^{(3)}$	Our Estimator (13) with $Y^{(0)}+Y^{(1)}+Y^{(2)}+Y^{(3)}$
7^3	1000	12.1741 (3.8190)	8.5599 (3.8415)	11.4690 (3.4460)	**3.9507 (1.3516)**
	2000	11.8920 (3.3524)	4.5767 (1.3534)	10.8306 (3.1022)	**2.2173 (0.6291)**
	5000	10.9300 (2.8547)	2.8012 (0.9527)	10.1989 (2.6965)	**1.8633 (0.5813)**
14^3	1000	7.6601 (2.5093)	2.2702 (0.8333)	7.3001 (2.1872)	**1.5684 (0.5730)**
	2000	7.2160 (2.4019)	1.7510 (0.6079)	7.0696 (2.0615)	**1.2402 (0.5062)**
	5000	6.9731 (2.3591)	1.4351 (0.5593)	6.2591 (1.9210)	**1.1468 (0.4213)**
21^3	1000	6.1625 (2.0150)	1.3341 (0.5150)	5.3861 (1.7399)	**1.0912 (0.3570)**
	2000	6.0483 (1.9180)	1.1994 (0.4180)	5.0355 (1.6164)	**0.8988 (0.2919)**
	5000	5.7112 (1.8264)	0.9541 (0.3654)	4.7698 (1.4877)	**0.7460 (0.2124)**

sample of 10^4 test points in $\mathcal{X}^3$. Since the true function $f_0(\mathbf{t})$ is unknown at each test point, we approximate it by using total $N = 19$ real ion channel samples at each test point. The function and gradient training data are generated using $N' = 10$ real ion channel samples, which are randomly chosen from the total $N = 19$ samples. Table S5 reports the MSEs and standard errors across varying sample size n, replications of the simulation q, and levels of gradient data. The results are summarized based on 1000 simulations for each scenario. Table S5 shows that our estimator outperforms Hall and Yatchew's estimator in terms of estimation accuracy.

Table S5: The average MSEs and standard errors of our estimator and those of Hall and Yatchew's estimator, considering various gradient types, for the example in Section 4.3 with 1000 simulations. The table shows metrics: "average MSE (standard error)," in units of 10^{-6}.

n	Hall and Yatchew with with only $Y^{(0)}$	Our Estimator (13) with with only $Y^{(0)}$	Hall and Yatchew with $Y^{(0)}+Y^{(1)}+Y^{(2)}+Y^{(3)}$	Our Estimator (13) with $Y^{(0)}+Y^{(1)}+Y^{(2)}+Y^{(3)}$
1000	11.0134 (5.6061)	10.6491 (4.9867)	8.6488 (4.4921)	**7.7804 (3.6737)**
2000	9.0626 (5.0207)	8.5302 (4.3339)	6.3674 (3.1476)	**5.1375 (2.4687)**
3000	7.0134 (4.2182)	6.4296 (3.9595)	5.0655 (2.4226)	**3.1035 (1.7187)**
5000	6.2315 (3.4613)	5.4143 (3.2268)	3.1745 (1.6182)	**2.1305 (0.9322)**

S4 Proofs of the Main Results

S4.1 Proof of Theorem 1

We prove a more general result in the following lemma. Let

$$l_n(f) \equiv \frac{1}{n}\sum_{i=1}^{n}\left[y_i^{(0)} - f(\mathbf{t}_i^{(0)})\right]^2 + \sum_{j=1}^{p} w_j \cdot \frac{1}{n}\sum_{i=1}^{n}\left[y_i^{(j)} - \frac{\partial f}{\partial t_j}(\mathbf{t}_i^{(j)})\right]^2.$$

Then the optimization problem (9) can be rewritten as,

$$\min_{f\in\mathcal{H}} l_n(f) \text{ subject to } \|f\|_{\mathcal{H}} \le R_n.$$

Lemma S1. *Let $f_{I,n}$ is the unique solution to the problem: $\min_{f\in\mathcal{H}} \|f\|_{\mathcal{H}}$ subject to $l_n(f) = 0$. Then, for $0 \le R_n < \|f_{I,n}\|_{\mathcal{H}}$, there exists a unique minimizer $\widehat{f}_n(\mathbf{t})$ of (9) in a finite-dimensional space. Specifically, there exist coefficients $\boldsymbol{\alpha}_j = (\alpha_{1j}, \ldots, \alpha_{nj})^\top \in \mathbb{R}^n$ for $j = 0, 1, \ldots, p$ such that,*

$$\widehat{f}_n(\mathbf{t}) = \sum_{i=1}^{n} \alpha_{i0} K_d(\mathbf{t}_i^{(0)}, \mathbf{t}) + \sum_{j=1}^{p}\sum_{i=1}^{n} \alpha_{ij} \frac{\partial K_d}{\partial t_j}(\mathbf{t}_i^{(j)}, \mathbf{t}), \tag{S2}$$

and $\|\widehat{f}_n\|_{\mathcal{H}} = R_n$. For $R_n \ge \|f_{I,n}\|_{\mathcal{H}}$, $\widehat{f}_n(\mathbf{t})$ in (S2) *is one of the minimizers of* (9).

Proof. Following the proof of Lemma 1 and Proposition 3 of Lim (2024), there exists a unique solution to the problem:

$$\min_{f\in\mathcal{H}} \|f\|_{\mathcal{H}} \text{ subject to } l_n(f) = 0,$$

which is denoted by $f_{I,n}$. Additionally, if $1 \le R_n < J(f_I)$, there exists a unique minimizer $\widehat{f}_n(\mathbf{t})$ of (9) that satisfies $\|\widehat{f}_n\|_{\mathcal{H}} = R_n$. A similar result can be found in Theorem 3 of Schoenberg (1964).

Since the optimization problem of (9) is convex, by Lagrangian duality, it can be reformulated as

$$\widehat{f}_n = \arg\min_{f\in\mathcal{H}} \left\{l_n(f) + \lambda\|f\|_{\mathcal{H}}^2\right\}.$$

Here, for a fixed set of function data and partial derivatives, the smoothing parameter $\lambda \ge 0$ is a function of the radius $R_n \ge 0$. Under the condition (7), the derivative $\partial f/\partial t_j$ is a bounded linear functional in $\mathcal{H}$. Following a similar argument to that of Theorem 1.3.1 in Wahba (1990), $\widehat{f}_n(\mathbf{t})$ takes the form in (S2). For $R_n \ge \|f_{I,n}\|_{\mathcal{H}}$, following the proof of Proposition 6 of Lim (2024), $\widehat{f}_n(\mathbf{t})$ in (S2) is one of the minimizers of (9). This completes the proof of Lemma S1. ■

Next, by Lemma S1, we know that for any $R_n \geq 0$, $\widehat{f}_n(\mathbf{t})$ in (S2) is a minimizer of (9) and it is in a finite-dimensional space spanned by $\{K_d(\mathbf{t}_i^{(0)}, \cdot), \frac{\partial K_d}{\partial t_j}(\mathbf{t}_i^{(j)}, \mathbf{t}); 1 \leq i \leq n, 1 \leq j \leq p\}$. This completes the proof of Theorem 1.

S4.2 Proof of Theorem 2

We establish the lower bound under random design via Fano's lemma (Tsybakov, 2009). It suffices to consider a particular case where the random errors $\epsilon^{(0)}$ and $\epsilon^{(j)}$s are independent Gaussian with zero mean and unit variance, and $\Pi^{(0)}$ and $\Pi^{(j)}$s are uniform distributions, and $\mathcal{H}_1$ is generated by periodic kernels. The lower bound established for this case is at least for the general cases (Tsybakov, 2009).

Let N be a natural number whose value will be clear later. We first derive the eigenvalue decay rate for the kernel K_d, which generates the RKHS $\mathcal{H}$. We introduce some additional notation. Define a family of the multi-index $\vec{\boldsymbol{\nu}}$ by

$$\mathbb{V} = \{\vec{\boldsymbol{\nu}} = (\nu_1, \dots, \nu_d)^\top \in \mathbb{N}^d, \text{ where at most } r \geq 1 \text{ of } \nu_k\text{s are not } 1\}. \tag{S3}$$

For a given $\tau > 0$, the number of multi-indices $\vec{\boldsymbol{\nu}} = (\nu_1, \dots, \nu_r) \in \mathbb{N}^r$ satisfying

$$\nu_1^{-2m} \cdots \nu_r^{-2m} \geq \tau$$

is the same as the number of multi-indices such that $\nu_1 \cdots \nu_r \leq \tau^{-1/(2m)}$, which amounts to

$$\begin{aligned} \sum_{\nu_2 \cdots \nu_r \leq \tau^{-1/(2m)}} \tau^{-1/(2m)}/(\nu_2 \cdots \nu_r) &= \tau^{-1/(2m)} \left(\sum_{\nu \leq \tau^{-1/(2m)}} 1/\nu \right)^{r-1} \\ &\asymp \tau^{-1/(2m)} (\log 1/\tau)^{r-1}. \end{aligned} \tag{S4}$$

Denote by $\lambda_N(K_d)$ the Nth eigenvalues of K_d. By inverting (S4), we obtain

$$\lambda_N(K_d) \asymp \left[N (\log N)^{1-r} \right]^{-2m}.$$

Hence, the multi-indices $\vec{\boldsymbol{\nu}} = (\nu_1, \dots, \nu_r) \in \mathbb{N}^r$ satisfying $\nu_1 \cdots \nu_r \leq N$ correspond to the first

$$c_0 N (\log N)^{r-1}$$

eigenvalues of K_d, for some constant c_0. Let b be a length-$\{c_0 N (\log N)^{r-1}\}$ binary sequence,

$$b = \{b_{\vec{\boldsymbol{\nu}}} : \nu_1 \cdots \nu_r \leq N\} \in \{0, 1\}^{c_0 N (\log N)^{r-1}}.$$

Let $\{\tilde{\lambda}_{\vec{\boldsymbol{\nu}}} : \nu_1 \cdots \nu_r \le N\}$ be the first $c_0 N(\log N)^{r-1}$ eigenvalues of K_d. Denote by

$$\{\tilde{\lambda}_{\vec{\boldsymbol{\nu}}+c_0N(\log N)^{r-1}} : \nu_1 \cdots \nu_r \le N\}$$

the $\{c_0N(\log N)^{r-1}+1\}$th, $\{c_0N(\log N)^{r-1}+2\}$th,..., $\{2c_0N(\log N)^{r-1}\}$th eigenvalues of K_d.

For brevity, we only prove for the case $p = d$ and $r \ge 3$. The other cases $p = d$, $r \le 2$ and $0 \le p < d$ can be showed similarly. Write

$$\begin{aligned} f_b(t_1, \ldots, t_r) = N^{-\frac{1}{2}+\frac{1}{r}} \sum_{\nu_1\cdots\nu_r \le N} & b_{\vec{\boldsymbol{\nu}}} \left(1 + \nu_1^2 + \cdots + \nu_r^2\right)^{-\frac{1}{2}} \\ & \times \tilde{\lambda}^{\frac{1}{2}}_{\vec{\boldsymbol{\nu}}+c_0N(\log N)^{r-1}} \psi_{\vec{\boldsymbol{\nu}}+c_0N(\log N)^{r-1}}(t_1, \ldots, t_r), \end{aligned}$$

where $\psi_{\vec{\boldsymbol{\nu}}+c_0N(\log N)^{r-1}}(t_1, \ldots, t_r)$ are the corresponding eigenfunctions of $\tilde{\lambda}_{\vec{\boldsymbol{\nu}}+c_0N(\log N)^{r-1}}$ of K_d. Note that

$$\begin{aligned} \|f_b\|_{\mathcal{H}}^2 &= N^{-1+\frac{2}{r}} \sum_{\nu_1\cdots\nu_r \le N} b_{\vec{\boldsymbol{\nu}}}^2 (1 + \nu_1^2 + \cdots + \nu_r^2)^{-1} \\ &\le N^{-1+\frac{2}{r}} \sum_{\nu_1\cdots\nu_r \le N} (1 + \nu_1^2 + \cdots + \nu_r^2)^{-1} \asymp 1, \end{aligned}$$

where the last step by Lemma S6, and this implies $f_b(\cdot) \in \mathcal{H}$.

By the Varshamov-Gilbert bound, e.g., Tsybakov (2009), there exists a collection of binary sequences $\{b^{(1)}, \ldots, b^{(M)}\} \subset \{0, 1\}^{c_0N(\log N)^{r-1}}$ such that

$$M \ge 2^{c_0N(\log N)^{r-1}/8},$$

and

$$H(b^{(l)}, b^{(q)}) \ge c_0N(\log N)^{r-1}/8, \quad \forall 1 \le l < q \le M.$$

Here $H(\cdot, \cdot)$ denotes the Hamming distance. Then, for $b^{(l)}, b^{(q)} \in \{0, 1\}^{c_0N(\log N)^{r-1}}$,

$$\begin{aligned} & \|f_{b^{(l)}} - f_{b^{(q)}}\|_{L_2}^2 \\ & \ge N^{-1+2/r}(2N)^{-2m} \sum_{\nu_1\cdots\nu_r \le N} (1 + \nu_1^2 + \cdots + \nu_r^2)^{-1} \left[b_{\vec{\boldsymbol{\nu}}}^{(l)} - b_{\vec{\boldsymbol{\nu}}}^{(q)}\right]^2 \\ & \ge N^{-1+2/r}(2N)^{-2m} \sum_{c_1 7N/8 \le \nu_1\cdots\nu_r \le N} (1 + \nu_1^2 + \cdots + \nu_r^2)^{-1} \\ & = c_2 N^{-2m} \end{aligned}$$

for some constants c_1 and c_2, where the last step is by Lemma S6.

On the other hand, for any $b^{(l)} \in \{b^{(1)}, \ldots, b^{(M)}\}$, again by Lemma S6,

$$\begin{aligned}\|f_{b^{(l)}}\|_{L_2}^2 + \sum_{j=1}^{p} \|\partial f_{b^{(l)}}/\partial t_j\|_{L_2}^2 &\le N^{-1+2/r} \sum_{\nu_1\cdots\nu_r \le N} \nu_1^{-2m} \cdots \nu_r^{-2m} \left[b_{\vec{\boldsymbol{\nu}}}^{(l)}\right]^2 \\ &\le N^{-1+2/r} \sum_{\nu_1\cdots\nu_r \le N} \nu_1^{-2m} \cdots \nu_r^{-2m} = c_3 N^{-2m+2/r} (\log N)^{r-1}\end{aligned}$$

for some constant c_3.

A standard argument gives that the lower bound can be reduced to the error probability in a multi-way hypothesis test (Tsybakov, 2009). Specifically, let Θ be a random variable uniformly distributed on $\{1, \ldots, M\}$. Note that

$$\inf_{\tilde{f}} \sup_{f_0 \in \mathcal{H}} \mathbb{P}\left\{ \|\tilde{f} - f_0\|_{L_2}^2 \ge \frac{1}{4} \min_{b^{(l)} \ne b^{(q)}} \|f_{b^{(l)}} - f_{b^{(q)}}\|_{L_2}^2 \right\} \ge \inf_{\widehat{\Theta}} \mathbb{P}\{\widehat{\Theta} \ne \Theta\}. \tag{S5}$$

The infimum on the right-hand side is taken over all decision rules that are measurable functions of the data. By Fano's lemma,

$$\begin{aligned}&\mathbb{P}\left\{ \widehat{\Theta} \ne \Theta | \mathbf{t}_1^{(0)}, \ldots, \mathbf{t}_n^{(0)}; \ldots; \mathbf{t}_1^{(p)}, \ldots, \mathbf{t}_n^{(p)} \right\} \\ &\quad \ge 1 - \frac{1}{\log M} \times \left[\mathbb{1}_{\mathbf{t}_1^{(0)}, \ldots, \mathbf{t}_n^{(0)}; \ldots; \mathbf{t}_1^{(p)}, \ldots, \mathbf{t}_n^{(p)}} (y_1^{(0)}, \ldots, y_n^{(0)}, \ldots, y_1^{(p)}, \ldots, y_n^{(p)}; \Theta) + \log 2 \right],\end{aligned} \tag{S6}$$

where

$$\mathbb{1}_{\mathbf{t}_1^{(0)}, \ldots, \mathbf{t}_n^{(0)}; \ldots; \mathbf{t}_1^{ep}, \ldots, \mathbf{t}_n^{ep}} (y_1^{(0)}, \ldots, y_n^{(0)}, \ldots, y_1^{(p)}, \ldots, y_n^{(p)})$$

is the mutual information between Θ and $\{y_1^{(0)}, \ldots, y_n^{(0)}, \ldots, y_1^{(p)}, \ldots, y_n^{(p)}\}$, and we fix the design points $\{\mathbf{t}_1^{(0)}, \ldots, \mathbf{t}_n^{(0)};\ \ldots; \mathbf{t}_1^{(p)}, \ldots, \mathbf{t}_n^{(p)}\}$. Thus,

$$\begin{aligned}&\mathbb{E}_{\mathbf{t}_1^{(0)}, \ldots, \mathbf{t}_n^{(0)}; \ldots; \mathbf{t}_1^{(p)}, \ldots, \mathbf{t}_n^{(p)}} \left[\mathbb{1}_{\mathbf{t}_1^{(0)}, \ldots, \mathbf{t}_n^{(0)}; \ldots; \mathbf{t}_1^{(p)}, \ldots, \mathbf{t}_n^{(p)}} \left(y_1^{(0)}, \ldots, y_n^{(0)}, \ldots, y_1^{(p)}, \ldots, y_n^{(p)}; \Theta \right) \right] \\ &\le \binom{M}{2}^{-1} \sum_{b^{(l)} \ne b^{(q)}} \mathbb{E}_{\mathbf{t}_1^{(0)}, \ldots, \mathbf{t}_n^{(0)}; \ldots; \mathbf{t}_1^{(p)}, \ldots, \mathbf{t}_n^{(p)}} \mathcal{K}\left(\mathbf{P}_{f_{b^{(l)}}} | \mathbf{P}_{f_{b^{(q)}}} \right) \\ &\le \frac{n(p+1)}{2} \binom{M}{2}^{-1} \sum_{b^{(l)} \ne b^{(q)}} \mathbb{E}_{\mathbf{t}_1^{(0)}, \ldots, \mathbf{t}_n^{(0)}; \ldots; \mathbf{t}_1^{(p)}, \ldots, \mathbf{t}_n^{(p)}} \|f_{b^{(l)}} - f_{b^{(q)}}\|_{*n}^2.\end{aligned} \tag{S7}$$

Here $\mathcal{K}(\cdot|\cdot)$ is the Kullback-Leibler distance, $\mathbf{P}_f$ is conditional distribution of $y_i^{(0)}$ and $y_i^{(j)}$s given $\{\mathbf{t}_1^{(0)}, \ldots, \mathbf{t}_n^{(0)}; \ldots; \mathbf{t}_1^{(p)}, \ldots, \mathbf{t}_n^{(p)}\}$, and the norm $\|\cdot\|_*$ is defined as follows,

$$\|f\|_{*n}^2 = \frac{1}{n(p+1)} \sum_{i=1}^{n} \left\{ [f(\mathbf{t}_i^{(0)})]^2 + \sum_{j=1}^{p} [\partial f(\mathbf{t}_i^{(j)})/\partial t_j]^2 \right\}, \quad \forall f : \mathcal{X}^r \mapsto \mathbb{R}.$$

Thus,

$$
\begin{aligned}
&\mathbb{E}_{\mathbf{t}_1^{(0)},\ldots,\mathbf{t}_n^{(0)};\ldots;\mathbf{t}_1^{(p)},\ldots,\mathbf{t}_n^{(p)}}\left[\mathbb{1}_{\mathbf{t}_1^{(0)},\ldots,\mathbf{t}_n^{(0)};\ldots;\mathbf{t}_1^{(p)},\ldots,\mathbf{t}_n^{(p)}}(y_1^{(0)},\ldots,y_n^{(0)},\ldots,y_1^{(p)},\ldots,y_n^{(p)};\Theta)\right] \\
&\quad\le \frac{n(p+1)}{2}\binom{M}{2}^{-1}\sum_{b^{(l)}\neq b^{(q)}}\left\{\|f_{b^{(l)}}-f_{b^{(q)}}\|_{L_2}^2+\sum_{j=1}^p\|\partial f_{b^{(l)}}/\partial t_j-\partial f_{b^{(q)}}/\partial t_j\|_{L_2}^2\right\} \\
&\quad\le \frac{n(p+1)}{2}\max_{b^{(l)}\neq b^{(q)}}\left\{\|f_{b^{(l)}}-f_{b^{(q)}}\|_{L_2}^2+\sum_{j=1}^p\|\partial f_{b^{(l)}}/\partial t_j-\partial f_{b^{(q)}}/\partial t_j\|_{L_2}^2\right\} \qquad \text{(S8)} \\
&\quad\le 2n(p+1)\max_{b^{(l)}\in\{b^{(1)},\ldots,b^{(M)}\}}\left\{\|f_{b^{(l)}}\|_{L_2}^2+\sum_{j=1}^p\|\partial f_{b^{(l)}}/\partial t_j\|_{L_2}^2\right\} \\
&\quad\le 2c_3 n(p+1)N^{-2m+\frac{2}{r}}(\log N)^{r-1}.
\end{aligned}
$$

Now, (S6) yields that

$$
\begin{aligned}
&\inf_{\tilde f}\sup_{f_0\in\mathcal{H}}\mathbb{P}\left\{\|\tilde f-f_0\|_{L_2}^2\ge\frac{1}{4}c_2N^{-2m}\right\} \\
&\ge \inf_{\widehat\Theta}\mathbb{P}\{\widehat\Theta\neq\Theta\} \\
&\ge 1-\frac{1}{\log M}\left[\mathbb{E}\mathbb{1}_{\mathbf{t}_1^{(0)},\ldots,\mathbf{t}_n^{(0)};\ldots;\mathbf{t}_1^{(p)},\ldots,\mathbf{t}_n^{(p)}}(y_1^{(0)},\ldots,y_n^{(0)},\ldots,y_1^{(p)},\ldots,y_n^{(p)};\Theta)+\log 2\right] \\
&\ge 1-\frac{2c_3n(p+1)N^{-2m+\frac{2}{r}}(\log N)^{r-1}+\log 2}{c_0(\log 2)N(\log N)^{r-1}/8}.
\end{aligned}
$$

Taking $N=c_4n^{r/(2mr+r-2)}$ with an appropriate choice of c_4, we have

$$
\limsup_{n\to\infty}\inf_{\tilde f}\sup_{f_0\in\mathcal{H}}\mathbb{P}\left\{\|\tilde f-f_0\|_{L_2}^2\ge cn^{-\frac{2mr}{(2m+1)r-2}}\right\}>0,
$$

where c does not depend on n. In addition, $\|\tilde f-f_0\|_{L_2}^2\ge\min_{\|f\|_{\mathcal{H}}\le R_n}\|f-f_0\|_{L_2}^2$. This completes the proof of this theorem.

S4.3 Proof of Theorem 3

Preliminaries. We consider a general quadratic penalty $J(\cdot)$ for the proposed method (9), where $J(\cdot)$ is any squared semi-norm on the RKHS $\mathcal{H}$. For example, when $\mathcal{H}_1=\mathcal{W}_2^m(\mathcal{X})$, it is common to choose $J(\cdot)$ for penalizing only the smooth component of a function. In this case, an explicit form of $J(\cdot)$ is presented in Wahba (1990). The following analysis holds for replacing $J(\cdot)$ with the squared norm $\|\cdot\|_{\mathcal{H}}^2$.

We define a new norm for any $f\in\mathcal{H}$,

$$
\|f\|_R^2=\frac{1}{p+1}\left[\frac{1}{\sigma_0^2}\int f^2(\mathbf{t})d\Pi^{(0)}(\mathbf{t})+\sum_{j=1}^p\frac{1}{\sigma_j^2}\int\left\{\frac{\partial f(\mathbf{t})}{\partial t_j}\right\}^2d\Pi^{(j)}(\mathbf{t})\right]+J(f). \qquad \text{(S9)}
$$

Note that $\|\cdot\|_R$ is a norm since it is a quadratic form and is equal to zero if and only if $f = 0$. Let $\langle\cdot,\cdot\rangle_R$ be the inner product associated with $\|\cdot\|_R$. Then by Lemma S7, the norm $\|\cdot\|_R$ is equivalent to the norm $\|\cdot\|_{\mathcal{H}}$ in RKHS $\mathcal{H}$. In particular, $\|f\|_R < \infty$ if and only if $\|f\|_{\mathcal{H}} < \infty$.

We introduce another norm $\|\cdot\|_0$ given by

$$\|f\|_0^2 = \frac{1}{p+1}\left[\frac{1}{\sigma_0^2}\int f^2(\mathbf{t})d\Pi^{(0)}(\mathbf{t}) + \sum_{j=1}^{p}\frac{1}{\sigma_j^2}\int\left\{\frac{\partial f(\mathbf{t})}{\partial t_j}\right\}^2 d\Pi^{(j)}(\mathbf{t})\right]. \tag{S10}$$

Let a function space F_0 be the direct sum of some set of the orthogonal subspaces in the decomposition of $\otimes_{j=1}^d L_2(\mathcal{X})$ as in (5) and equipped with the norm $\|\cdot\|_0$. Write $\langle\cdot,\cdot\rangle_0$ as the inner product associated with $\|\cdot\|_0$ in F_0.

Finally, we define the following norm. For $f \in \mathcal{H}$,

$$\|f\|_{L_2(a)}^2 = \sum_{\vec{\nu}\in\mathbb{V}}\left(1 + \frac{\rho_{\vec{\nu}}}{\|\phi_{\vec{\nu}}\|_{L_2}^2}\right)^a f_{\vec{\nu}}^2\|\phi_{\vec{\nu}}\|_{L_2}^2, \quad \text{for } 0 \le a \le 1, \tag{S11}$$

where $f_{\vec{\nu}} = \langle f, \phi_{\vec{\nu}}\rangle_0$. By direct calculations, when $a = 0$ this norm coincides with $\|\cdot\|_{L_2}$ on F_0, and when $a = 1$ this norm is equivalent to $\|\cdot\|_R$ on $\mathcal{H}$.

Denote the loss function in (9) by $l_n(f)$, that is,

$$l_n(f) = \frac{1}{n(p+1)}\left[\frac{1}{\sigma_0^2}\sum_{i=1}^{n}\{f(\mathbf{t}_i^{(0)}) - y_i^{(0)}\}^2 + \sum_{j=1}^{p}\frac{1}{\sigma_j^2}\sum_{i=1}^{n}\left\{\frac{\partial f(\mathbf{t}_i^{(j)})}{\partial t_j} - y_i^{(j)}\right\}^2\right],$$

and write $l_{n\lambda}(f) = l_n(f) + \lambda J(f)$. Then the estimator $\widehat{f}_n = \arg\min_{f\in\mathcal{H}} l_{n\lambda}(f)$. Denote the expected loss by $l_\infty(f) = \mathbb{E}l_n(f)$ and write $l_{\infty\lambda}(f) = l_\infty(f) + \lambda J(f)$. Since $l_{\infty\lambda}(f)$ a positive quadratic form in $f \in \mathcal{H}$, it has a unique minimizer in $\mathcal{H}$ given by

$$\bar{f}_{\infty\lambda} = \arg\min_{f\in\mathcal{H}} l_{\infty\lambda}(f).$$

Let $f^\dagger = \arg\min_{J(f)\le R_n^2}\|f - f_0\|_{L_2}^2$. Thus, we decompose

$$\widehat{f}_n - f_0 = (\widehat{f}_n - \bar{f}_{\infty\lambda}) + (\bar{f}_{\infty\lambda} - f^\dagger) + (f^\dagger - f_0), \tag{S12}$$

where $(\widehat{f}_n - \bar{f}_{\infty\lambda})$ is referred to *stochastic error*, $(\bar{f}_{\infty\lambda} - f^\dagger)$ is referred to *deterministic error*, and $(f^\dagger - f_0)$ is referred to *approximation error*; see, e.g., van der Vaart and Wellner (1996). We omit the subscripts of $\bar{f}_{\infty\lambda}$ and $\widehat{f}_n$ hereafter if no confusion occurs.

Outline of the proof. Since the distributions $\Pi^{(0)}$ and $\Pi^{(j)}$s are known, it suffices to consider the uniform distributions by the inverse transform sampling in Lemma S8. Moreover, since f_0 is a functional ANOVA model with component function spaces supported in a compact domain $\mathcal{X}^d \equiv [0,1]^d$, one can smoothly extend f_0 to a larger compactly supported domain $[0, 1+\delta]^d$ and achieve periodicity on the new boundary. This is proven in Lemma S9, which also shows that the eigenvalue decay rate for the RKHS associated with the extended periodic function remains the same as that for the RKHS associated with the original function. Moreover, the probability of selecting $\mathbf{t}$ within the range $\{[0, 1+\delta]^d \setminus \mathcal{X}^d\}$ is $O(\delta)$, which is negligible for a sufficiently small δ. Lemma S10 shows that the estimation error of f_0 can be upper bounded by that of the extended periodic function. Hence, the upper bound of the estimation for the periodic function also applies to the original function f_0. Therefore, we consider f_0 has a periodic boundary in $\mathcal{X}^d$ in the proof. A similar technique has been used in literature; e.g., Hall and Yatchew (2010).

Write the trigonometrical basis on $L_2(\mathcal{X})$ as $\psi_1(t) = 1$, $\psi_{2\nu}(t) = \sqrt{2}\cos 2\pi\nu t$ and $\psi_{2\nu+1}(t) = \sqrt{2}\sin 2\pi\nu t$ for $\nu \geq 1$. Let

$$\phi_{\vec{\nu}}(t_1, \ldots, t_d) = \frac{\psi_{\nu_1}(t_1)\cdots\psi_{\nu_d}(t_d)}{\|\psi_{\nu_1}(t_1)\cdots\psi_{\nu_d}(t_d)\|_0}. \tag{S13}$$

Since f_0 has a periodic boundary in $\mathcal{X}^d$ and $\pi^{(j)} \equiv 1$, $\{\phi_{\vec{\nu}}(\mathbf{t}) : \vec{\boldsymbol{\nu}} \in \mathbb{V}\}$, where $\mathbb{V}$ in (S3) forms an orthogonal basis for $\mathcal{H}$ in $\langle\cdot,\cdot\rangle_R$; an orthogonal system for $L_2(\mathcal{X}^d)$; and an orthonormal basis for F_0 in $\langle\cdot,\cdot\rangle_0$, that is $\langle\phi_{\vec{\nu}}(\mathbf{t}), \phi_{\vec{\mu}}(\mathbf{t})\rangle_0 = \delta_{\vec{\nu}\vec{\mu}}$, where $\delta_{\vec{\nu}\vec{\mu}}$ is Kronecker's delta; see, e.g., Chapter 2 in Wahba (1990). The concept of simultaneous orthogonality of a basis in multiple inner product spaces has been explored in other RKHS settings; see, e.g., Section 3 in Yuan and Cai (2010). Hence, any $f \in \mathcal{H}$ has the decomposition

$$f(t_1, \ldots, t_d) = \sum_{\vec{\boldsymbol{\nu}} \in \mathbb{V}} f_{\vec{\nu}}\phi_{\vec{\nu}}(t_1, \ldots, t_d), \qquad \text{where } f_{\vec{\nu}} = \langle f(\mathbf{t}), \phi_{\vec{\nu}}(\mathbf{t})\rangle_0. \tag{S14}$$

We denote a positive scalar series $\{\rho_{\vec{\nu}}\}_{\boldsymbol{\nu}\in\mathbb{V}}$ such that $\langle\phi_{\vec{\nu}}, \phi_{\vec{\mu}}\rangle_R = (1+\rho_{\vec{\nu}})\delta_{\vec{\nu}\vec{\mu}}$. Then,

$$J(f) = \langle f, f\rangle_R - \langle f, f\rangle_0 = \sum_{\vec{\boldsymbol{\nu}}\in\mathbb{V}} \rho_{\vec{\nu}} f_{\vec{\nu}}^2. \tag{S15}$$

First, we analyze the deterministic error $(\bar{f} - f^\dagger)$. By (S14), write $f^\dagger(\mathbf{t}) = \sum_{\vec{\boldsymbol{\nu}}\in\mathbb{V}} f_{\vec{\nu}}^\dagger \phi_{\vec{\nu}}(\mathbf{t})$ and $\bar{f}(\mathbf{t}) = \sum_{\vec{\boldsymbol{\nu}}\in\mathbb{V}} \bar{f}_{\vec{\nu}}\phi_{\vec{\nu}}(\mathbf{t})$. Note the bias satisfies $\mathbb{E}[\epsilon_i^{(j)}] = o(n^{-1/2})$, we have

$$l_\infty(f) = \sum_{\vec{\boldsymbol{\nu}}\in\mathbb{V}} (f_{\vec{\nu}} - f_{\vec{\nu}}^\dagger)^2 + o(n^{-1/2})\sqrt{\sum_{\vec{\boldsymbol{\nu}}\in\mathbb{V}} (f_{\vec{\nu}} - f_{\vec{\nu}}^\dagger)^2 + 1},$$

and

$$\bar{f}_{\vec{\boldsymbol{\nu}}} = \frac{f^{\dagger}_{\vec{\boldsymbol{\nu}}}(1+\kappa_{\vec{\boldsymbol{\nu}}})}{1+\kappa_{\vec{\boldsymbol{\nu}}}+\lambda\rho_{\vec{\boldsymbol{\nu}}}}, \quad \text{where } \kappa_{\vec{\boldsymbol{\nu}}} = o(1),\ \forall \vec{\boldsymbol{\nu}} \in \mathbb{V}. \tag{S16}$$

An upper bound of the deterministic error will be given in Lemma S2.

Second, we analyze the stochastic error $(\widehat{f}_n - \bar{f})$. The existence of the following Fréchet derivatives is guaranteed by Lemma S3:

$$\begin{aligned} Dl_n(f)g = \frac{2}{n(p+1)} \Bigg[& \frac{1}{\sigma_0^2} \sum_{i=1}^{n} \{f(\mathbf{t}_i^{(0)}) - y_i^{(0)}\} g(\mathbf{t}_i^{(0)}) \\ & + \sum_{j=1}^{p} \frac{1}{\sigma_j^2} \sum_{i=1}^{n} \left\{ \frac{\partial f(\mathbf{t}_i^{(j)})}{\partial t_j} - y_i^{(j)} \right\} \frac{\partial g(\mathbf{t}_i^{(j)})}{\partial t_j} \Bigg], \end{aligned} \tag{S17}$$

$$\begin{aligned} Dl_\infty(f)g = \frac{2}{p+1} \Bigg[& \frac{1}{\sigma_0^2} \int \left\{ f(\mathbf{t}) - f_0(\mathbf{t}) + o(n^{-1/2}) \right\} g(\mathbf{t}) d\Pi^{(0)}(\mathbf{t}) \\ & + \sum_{j=1}^{p} \frac{1}{\sigma_j^2} \int \left\{ \frac{\partial f(\mathbf{t})}{\partial t_j} - \frac{\partial f_0(\mathbf{t})}{\partial t_j} + o(n^{-1/2}) \right\} \frac{\partial g(\mathbf{t})}{\partial t_j} d\Pi^{(j)}(\mathbf{t}) \Bigg], \end{aligned} \tag{S18}$$

$$\begin{aligned} D^2 l_n(f)gh = \frac{2}{n(p+1)} \Bigg[& \frac{1}{\sigma_0^2} \sum_{i=1}^{n} g(\mathbf{t}_i^{(0)}) h(\mathbf{t}_i^{(0)}) \\ & + \sum_{j=1}^{p} \frac{1}{\sigma_j^2} \sum_{i=1}^{n} \frac{\partial g(\mathbf{t}_i^{(j)})}{\partial t_j} \frac{\partial h(\mathbf{t}_i^{(j)})}{\partial t_j} \Bigg], \end{aligned} \tag{S19}$$

$$\begin{aligned} D^2 l_\infty(f)gh = \frac{2}{p+1} \Bigg[& \frac{1}{\sigma_0^2} \int g(\mathbf{t}) h(\mathbf{t}) d\Pi^{(0)}(\mathbf{t}) \\ & + \sum_{j=1}^{p} \frac{1}{\sigma_j^2} \int \frac{\partial g(\mathbf{t})}{\partial t_j} \frac{\partial h(\mathbf{t})}{\partial t_j} d\Pi^{(j)}(\mathbf{t}) \Bigg] = 2\langle g, h \rangle_0, \end{aligned} \tag{S20}$$

where $Dl_n(f)$, $Dl_\infty(f)$, $D^2 l_n(f)g$, and $D^2 l_\infty(f)g$ are bounded linear operators on $\mathcal{H}$. By Riesz representation theorem, with a slight abuse of notation, write

$$\begin{aligned} Dl_n(f)g = \langle Dl_n(f), g \rangle_R, \quad Dl_\infty(f)g = \langle Dl_\infty(f), g \rangle_R, \\ D^2 l_n(f)gh = \langle D^2 l_n(f)g, h \rangle_R, \quad D^2 l_\infty(f)gh = \langle D^2 l_\infty(f)g, h \rangle_R. \end{aligned}$$

From Oden and Reddy (2012), there exists a bounded linear operator $U : F_0 \mapsto \mathcal{H}$ such that $U\phi_{\vec{\boldsymbol{\nu}}} = (1+\rho_{\vec{\boldsymbol{\nu}}})^{-1}\phi_{\vec{\boldsymbol{\nu}}}$ and $\langle f, Ug \rangle_R = \langle f, g \rangle_0$ for any $f \in \mathcal{H}$ and $g \in F_0$, and the restriction of U to $\mathcal{H}$ is self-adjoint and positive definite. By (S20), we further derive

$$D^2 l_{\infty\lambda}(f)\phi_{\vec{\boldsymbol{\nu}}}(\mathbf{t}) = 2(U + \lambda(I-U))\phi_{\vec{\boldsymbol{\nu}}}(\mathbf{t}) = 2(1+\rho_{\vec{\boldsymbol{\nu}}})^{-1}(1+\lambda\rho_{\vec{\boldsymbol{\nu}}})\phi_{\vec{\boldsymbol{\nu}}}(\mathbf{t}).$$

Define that $G_\lambda \phi_{\vec{\boldsymbol{\nu}}} = \frac{1}{2} D^2 l_{\infty\lambda}(\bar{f})\phi_{\vec{\boldsymbol{\nu}}}$. By the Lax-Milgram theorem, $G_\lambda : \mathcal{H} \mapsto \mathcal{H}$ has a bounded inverse G_λ^{-1} on $\mathcal{H}$, and

$$G_\lambda^{-1}\phi_{\vec{\boldsymbol{\nu}}} = (1+\rho_{\vec{\boldsymbol{\nu}}})(1+\lambda\rho_{\vec{\boldsymbol{\nu}}})^{-1}\phi_{\vec{\boldsymbol{\nu}}}. \tag{S21}$$

Define

$$\tilde{f}^* = \bar{f} - \frac{1}{2} G_\lambda^{-1} D l_{n\lambda}(\bar{f}).$$

Then the stochastic error can be decomposed as

$$\widehat{f}_n - \bar{f} = (\tilde{f}^* - \bar{f}) + (\widehat{f}_n - \tilde{f}^*).$$

The two terms on the right-hand side will be studied separately, and their upper bounds will be given in Lemma S4 and Lemma S5, respectively.

Main proof. Now, we give the details by following the above outline. First, we present an upper bound of the deterministic error $(\bar{f} - f^\dagger)$ in (S12).

Lemma S2. *For any $0 \le a \le 1$, the deterministic error in (S12) satisfies*

$$\|\bar{f} - f^\dagger\|^2_{L_2(a)} = \begin{cases} O\left\{\lambda^{1-a} R_n^2\right\} & \text{when } 0 \le p < d, \\ O\{\lambda^{\frac{(1-a)mr}{mr-1}} R_n^2\} & \text{when } p = d. \end{cases}$$

Proof. We first introduce some notations. For two positive sequences a_n and b_n, we write $a_n \lesssim b_n$ (or $a_n \gtrsim b_n$) means that there exists a constant $c > 0$ (or $c' > 0$) such that $a_n \le c b_n$ (or $a_n \ge c' b_n$) for all n. We write $a_n \asymp b_n$ if a_n/b_n is bounded away from both zero and infinity as $n \to \infty$.

For any $0 \le a \le 1$, by (S15) and (S16), we have

$$\begin{aligned} \|\bar{f} - f^\dagger\|^2_{L_2(a)} &= \sum_{\vec{\boldsymbol{\nu}} \in \mathbb{V}} \left(1 + \frac{\rho_{\vec{\boldsymbol{\nu}}}}{\|\phi_{\vec{\boldsymbol{\nu}}}\|^2_{L_2}}\right)^a \left(\frac{\lambda\rho_{\vec{\boldsymbol{\nu}}}}{1+\kappa_{\vec{\boldsymbol{\nu}}} + \lambda\rho_{\vec{\boldsymbol{\nu}}}}\right)^2 (f^\dagger_{\vec{\boldsymbol{\nu}}})^2 \|\phi_{\vec{\boldsymbol{\nu}}}\|^2_{L_2} \\ &\lesssim \lambda^2 \sup_{\vec{\boldsymbol{\nu}} \in \mathbb{V}} \frac{(1+\rho_{\vec{\boldsymbol{\nu}}}/\|\phi_{\vec{\boldsymbol{\nu}}}\|^2_{L_2})^a \rho_{\vec{\boldsymbol{\nu}}} \|\phi_{\vec{\boldsymbol{\nu}}}\|^2_{L_2}}{(1+\lambda\rho_{\vec{\boldsymbol{\nu}}})^2} \sum_{\vec{\boldsymbol{\nu}} \in \mathbb{V}} \rho_{\vec{\boldsymbol{\nu}}} (f^\dagger_{\vec{\boldsymbol{\nu}}})^2 \qquad \text{(S22)} \\ &\lesssim \lambda^2 R_n^2 \sup_{\vec{\boldsymbol{\nu}} \in \mathbb{V}} \frac{(\prod_{k=1}^d \nu_k^{2m})^{1+a}}{(1+\sum_{j=1}^p \nu_j^2 + \lambda \prod_{k=1}^d \nu_k^{2m})^2}. \end{aligned}$$

Write

$$B_\lambda(\vec{\boldsymbol{\nu}}) = \frac{(\prod_{k=1}^d \nu_k^{2m})^{1+a}}{(1+\sum_{j=1}^p \nu_j^2 + \lambda \prod_{k=1}^d \nu_k^{2m})^2}, \quad \vec{\boldsymbol{\nu}} \in \mathbb{V}.$$

We discuss $B_\lambda(\vec{\boldsymbol{\nu}})$ for $0 \le p \le d-1$ and $p = d$ separately.

For $0 \le p \le d-1$, since $\vec{\boldsymbol{\nu}} \in \mathbb{V}$, there are at most r of $\nu_1, \dots, \nu_d$ not equal to 1. Suppose for any $x = \prod_{k=1}^{d} \nu_k^{-2m} > 0$ fixed. Then $B_\lambda(\vec{\boldsymbol{\nu}})$ is maximized by letting $\sum_{j=1}^{p} \nu_j^2$ be as small as possible, which implies $\nu_1 = \nu_2 = \cdots = \nu_p = 1$. Then,

$$
\begin{aligned}
\sup_{\vec{\boldsymbol{\nu}} \in \mathbb{V}} B_\lambda(\vec{\boldsymbol{\nu}}) &\asymp \sup_{(\nu_{p+1},\dots,\nu_{(p+r)\wedge d})^\top \in \mathbb{N}^{r \wedge (d-p)}} \frac{\prod_{k=p+1}^{(p+r)\wedge d} \nu_k^{2m(1+a)}}{(1+\lambda \prod_{k=p+1}^{(p+r)\wedge d} \nu_k^{2m})^2} \\
&\asymp \sup_{x>0} \frac{x^{-(1+a)}}{(1+\lambda x^{-1})^2} \asymp \lambda^{-(a+1)},
\end{aligned}
\tag{S23}
$$

where the last step is achieved when $x \asymp \lambda$.

For $p = d$, since $\vec{\boldsymbol{\nu}} \in \mathbb{V}$ and by the symmetry of coordinates $v_1, \dots, v_d$, assume that all indices except $v_1, \dots, v_r$ being 1. Letting $z = \prod_{j=1}^{r} \nu_j^{-2m} > 0$, we have

$$
\sup_{\vec{\boldsymbol{\nu}} \in \mathbb{V}} B_\lambda(\vec{\boldsymbol{\nu}}) \asymp \sup_{z>0} \frac{z^{-(1+a)}}{(z^{-1/mr} + \lambda z^{-1})^2} \asymp \lambda^{\frac{2-(1+a)mr}{mr-1}},
\tag{S24}
$$

where the last step is achieved when $z \asymp \lambda^{mr/(mr-1)}$. Combining (S22), (S23) and (S24) we complete the proof. ■

Before we establish an upper bound for the stochastic error, we present the Fréchet derivative of the operator that will be used in the proof. Let X and Y be the normed linear spaces. The Fréchet derivative of an operator $F : X \mapsto Y$ is a bounded linear operator $DF(a) : X \mapsto Y$ with

$$
\lim_{h \to 0, h \in X} \frac{\|F(a+h) - F(a) - DF(a)h\|_Y}{\|h\|_X} = 0.
$$

For example, if $F(a+h) - F(a) = Lh + R(a,h)$ with a linear operator L and

$$
\frac{\|R(a,h)\|_Y}{\|h\|_X} \to 0, \quad \text{as } h \to 0,
$$

by definition then $L = DF(a)$ is the Fréchet derivative of $F(\cdot)$. The reader is referred to Gelfand and Silverman (2000) for a thorough investigation of the Fréchet derivative. We give the Fréchet derivative of the operator in our setting.

Lemma S3. *Denote the loss function in (9) by $l_n(f)$. With the norm $\|\cdot\|_R$ in (S9), the*

first-order Fréchet derivative of the functional $l_n(\cdot)$ for any $f, g \in \mathcal{H}$ is

$$Dl_n(f)g = \frac{2}{n(p+1)} \left[\frac{1}{\sigma_0^2} \sum_{i=1}^n \{f(\mathbf{t}_i^{(0)}) - y_i^{(0)}\} g(\mathbf{t}_i^{(0)}) + \sum_{j=1}^p \frac{1}{\sigma_j^2} \sum_{i=1}^n \left\{ \frac{\partial f(\mathbf{t}_i^{(j)})}{\partial t_j} - y_i^{(j)} \right\} \frac{\partial g(\mathbf{t}_i^{(j)})}{\partial t_j} \right].$$

The second-order Fréchet derivative of $l_n(\cdot)$ for any $f, g, h \in \mathcal{H}$ is

$$D^2 l_n(f)gh = \frac{2}{n(p+1)} \left[\frac{1}{\sigma_0^2} \sum_{i=1}^n g(\mathbf{t}_i^{(0)}) h(\mathbf{t}_i^{(0)}) + \sum_{j=1}^p \frac{1}{\sigma_j^2} \sum_{i=1}^n \frac{\partial g(\mathbf{t}_i^{(j)})}{\partial t_j} \frac{\partial h(\mathbf{t}_i^{(j)})}{\partial t_j} \right].$$

Proof. By direct calculations, we have

$$l_n(f+g) - l_n(f) = \frac{2}{n(p+1)} \left[\frac{1}{\sigma_0^2} \sum_{i=1}^n \{f(\mathbf{t}_i^{(0)}) - y_i^{(0)}\} g(\mathbf{t}_i^{(0)}) + \sum_{j=1}^p \frac{1}{\sigma_j^2} \sum_{i=1}^n \left\{ \frac{\partial f(\mathbf{t}_i^{(j)})}{\partial t_j} - y_i^{(j)} \right\} \frac{\partial g(\mathbf{t}_i^{(j)})}{\partial t_j} \right] + \mathcal{R}_n(f, g),$$

where

$$\begin{aligned} \mathcal{R}_n(f,g) &= \frac{1}{n(p+1)} \left[\frac{1}{\sigma_0^2} \sum_{i=1}^n g^2(\mathbf{t}_i^{(0)}) + \sum_{j=1}^p \frac{1}{\sigma_j^2} \sum_{i=1}^n \left\{ \frac{\partial g(\mathbf{t}_i^{(j)})}{\partial t_j} \right\}^2 \right] \\ &= \|g\|_0^2 + O(n^{-1/2}), \end{aligned}$$

and the $\|\cdot\|_0$ norm is given in (S10). Note that $|\mathcal{R}_n(f,g)|/\|g\|_R \to 0$ as $\|g\|_R \to 0$ and $n^{1/2}\|g\|_R \to \infty$. This proves the first part of the lemma. For the second-order Fréchet derivative, note that

$$\begin{aligned} & Dl_n(f+h)g - Dl_n(f)g \\ &= \frac{2}{n(p+1)} \left[\frac{1}{\sigma_0^2} \sum_{i=1}^n g(\mathbf{t}_i^{(0)}) h(\mathbf{t}_i^{(0)}) + \sum_{j=1}^p \frac{1}{\sigma_j^2} \sum_{i=1}^n \frac{\partial g(\mathbf{t}_i^{(j)})}{\partial t_j} \frac{\partial h(\mathbf{t}_i^{(j)})}{\partial t_j} \right], \end{aligned}$$

which is linear in h. By definition, the $D^2 l_n(f)gh$ in the lemma is the valid second-order Fréchet derivative of $l_n(\cdot)$. ■

By following a similar derivation for Lemma S3, it is easy to obtain the first and the second-order Fréchet derivatives of the functional $l_\infty(\cdot)$ in (S18) and (S20), respectively.

We now establish an upper bound for the term $(\tilde{f}^* - \bar{f})$, which is a part of the stochastic error.

Lemma S4. *When $0 \le p < d$, we have for any $0 \le a < 1 - 1/2m$,*

$$\|\tilde{f}^* - \bar{f}\|_{L_2(a)}^2 = O_{\mathbb{P}}\left\{n^{-1}\lambda^{-(a+1/2m)}[\log(1/\lambda)]^{(d-p)\wedge r-1}\right\}.$$

When $p = d$, we have for any $0 \le a \le 1$,

$$\begin{aligned}
&\|\tilde{f}^* - \bar{f}\|_{L_2(a)}^2 \\
&= \begin{cases} O_{\mathbb{P}}\left\{n^{-1}R_n^2\lambda^{\frac{mr}{1-mr}\left(a+\frac{r-2}{2mr}\right)}\right\}, \ \text{if } r \ge 3; \\ O_{\mathbb{P}}\left\{n^{-1}R_n^2\log(1/\lambda)\right\}, \ \text{if } r = 2, a = 0; \quad O_{\mathbb{P}}\left\{n^{-1}R_n^2\right\}, \ \text{if } r = 2, 0 < a \le 1; \\ O_{\mathbb{P}}\left\{n^{-1}R_n^2\right\}, \ \text{if } r = 1, a < \frac{1}{2m}; \quad O_{\mathbb{P}}\left\{n^{-1}\log(1/\lambda)R_n^2\right\}, \ \text{if } r = 1, a = \frac{1}{2m}; \\ O_{\mathbb{P}}\left\{n^{-1}\lambda^{\frac{1-2ma}{2m-2}}R_n^2\right\}, \ \text{if } r = 1, a > \frac{1}{2m}. \end{cases}
\end{aligned}$$

Proof. Notice that $Dl_{n,\lambda}(\bar{f}) = Dl_{n,\lambda}(\bar{f}) - Dl_{\infty,\lambda}(\bar{f}) = Dl_n(\bar{f}) - Dl_\infty(\bar{f})$. Hence, for any $g \in \mathcal{H}$,

$$\begin{aligned}
&\mathbb{E}\left[\frac{1}{2}Dl_{n,\lambda}(\bar{f})g\right]^2 = \mathbb{E}\left[\frac{1}{2}Dl_n(\bar{f})g - \frac{1}{2}Dl_\infty(\bar{f})g\right]^2 \\
&\lesssim \frac{1}{n(p+1)^2}\sum_{j=0}^p \mathrm{Var}\left[\frac{1}{\sigma_j^2}\left\{\frac{\partial \bar{f}(\mathbf{t}^{(j)})}{\partial t_j} - Y^{(j)}\right\}\frac{\partial g(\mathbf{t}^{(j)})}{\partial t_j}\right] \\
&+ \sum_{j=0}^p \frac{\sigma_j^{-4}}{n^2(p+1)^2}\sum_{i\neq i'}\mathrm{Cov}\left[\left(\frac{\partial \bar{f}(\mathbf{t}_i^{(j)})}{\partial t_j} - y_i^{(j)}\right)\frac{\partial g(\mathbf{t}_i^{(j)})}{\partial t_j}, \left(\frac{\partial \bar{f}(\mathbf{t}_{i'}^{(j)})}{\partial t_j} - y_{i'}^{(j)}\right)\frac{\partial g(\mathbf{t}_{i'}^{(j)})}{\partial t_j}\right] \\
&+ \sum_{j\neq k}\frac{\sigma_j^{-2}\sigma_k^{-2}}{n^2(p+1)^2}\sum_{i,i'=1}^n \mathrm{Cov}\left[\left(\frac{\partial \bar{f}(\mathbf{t}_i^{(j)})}{\partial t_j} - y_i^{(j)}\right)\frac{\partial g(\mathbf{t}_i^{(j)})}{\partial t_j}, \left(\frac{\partial \bar{f}(\mathbf{t}_{i'}^{(k)})}{\partial t_k} - y_{i'}^{(j)}\right)\frac{\partial g(\mathbf{t}_{i'}^{(k)})}{\partial t_k}\right],
\end{aligned} \tag{S25}$$

where the second step is due to $\sum_{i\neq i'}\mathrm{Cov}[\epsilon_i^{(j)}, \epsilon_{i'}^{(k)}] = \sum_{i\neq i'} o(|i-i'|^{-\Upsilon}) = o(n)$. Note that (S25) can be further bounded up to some constant by,

$$\begin{aligned}
&\frac{1}{n(p+1)}\left[\frac{1}{\sigma_0^4}\mathbb{E}\left\{\bar{f}(\mathbf{t}^{(0)}) - f_0(\mathbf{t}^{(0)})\right\}^2\{g(\mathbf{t}^{(0)})\}^2 + \frac{1}{\sigma_0^2}\mathbb{E}\{g(\mathbf{t}^{(0)})\}^2\right. \\
&\left.+\sum_{j=1}^p \frac{1}{\sigma_j^4}\mathbb{E}\left\{\frac{\partial \bar{f}(\mathbf{t}^{(j)})}{\partial t_j} - \frac{\partial f_0(\mathbf{t}^{(j)})}{\partial t_j}\right\}^2\left\{\frac{\partial g(\mathbf{t}^{(j)})}{\partial t_j}\right\}^2 + \sum_{j=1}^p \frac{1}{\sigma_j^2}\mathbb{E}\left\{\frac{\partial g(\mathbf{t}^{(j)})}{\partial t_j}\right\}^2\right] \\
&+ o(n^{-1})\frac{1}{(p+1)^2}\sum_{j,k=0}^p \mathbb{E}\left[\frac{\partial g(\mathbf{t}^{(j)})}{\partial t_j}\right]\mathbb{E}\left[\frac{\partial g(\mathbf{t}^{(k)})}{\partial t_k}\right],
\end{aligned} \tag{S26}$$

By Lemma S7, Lemma S11, and Cauchy-Schwarz inequality, we have that (S26) is bounded

up to some constant by

$$
\begin{aligned}
&\frac{1}{n(p+1)}\left[\frac{1}{\sigma_0^4}c_K^{2d}\|\bar{f}-f_0\|_R^2\mathbb{E}\left\{g(\mathbf{t}^{(0)})\right\}^2+\frac{1}{\sigma_0^2}\mathbb{E}\left\{g(\mathbf{t}^{(0)})\right\}^2\right.\\
&\left.+\sum_{j=1}^{p}\frac{1}{\sigma_j^4}c_K^{2d}\|\bar{f}-f_0\|_R^2\mathbb{E}\left\{\frac{\partial g(\mathbf{t}^{(j)})}{\partial t_j}\right\}^2+\sum_{j=0}^{p}\frac{1}{\sigma_j^2}\mathbb{E}\left\{\frac{\partial g(\mathbf{t}^{(j)})}{\partial t_j}\right\}^2\right] \qquad \text{(S27)}\\
&\lesssim n^{-1}R_n^2\|g\|_0^2,
\end{aligned}
$$

where the last step above is by Lemma S2 and the definition of the norm $\|\cdot\|_0$. From the definition of G_λ^{-1} in (S21), we have that $\forall g\in\mathcal{H}$,

$$
\left\|G_\lambda^{-1}g\right\|_{L_2(a)}^2=\sum_{\vec{\nu}\in\mathbb{V}}\left(1+\frac{\rho_{\vec{\nu}}}{\|\phi_{\vec{\nu}}\|_{L_2}^2}\right)^a(1+\lambda\rho_{\vec{\nu}})^{-2}\|\phi_{\vec{\nu}}\|_{L_2}^2\langle g,\phi_{\vec{\nu}}\rangle_R^2.
$$

Then by the definition of $\tilde{f}^*$,

$$
\begin{aligned}
&\mathbb{E}\|\tilde{f}^*-\bar{f}\|_{L_2(a)}^2=\mathbb{E}\left\|\frac{1}{2}G_\lambda^{-1}Dl_{n\lambda}(\bar{f})\right\|_{L_2(a)}^2\\
&=\frac{1}{4}\mathbb{E}\left[\sum_{\vec{\nu}\in\mathbb{V}}\left(1+\frac{\rho_{\vec{\nu}}}{\|\phi_{\vec{\nu}}\|_{L_2}^2}\right)^a(1+\lambda\rho_{\vec{\nu}})^{-2}\|\phi_{\vec{\nu}}\|_{L_2}^2\langle Dl_{n\lambda}(\bar{f}),\phi_{\vec{\nu}}\rangle_R^2\right]\\
&\leq\sum_{\vec{\nu}\in\mathbb{V}}\left(1+\frac{\rho_{\vec{\nu}}}{\|\phi_{\vec{\nu}}\|_{L_2}^2}\right)^a(1+\lambda\rho_{\vec{\nu}})^{-2}\|\phi_{\vec{\nu}}\|_{L_2}^2\mathbb{E}\left[\frac{1}{2}Dl_{n\lambda}(\bar{f})\phi_{\vec{\nu}}\right]^2\\
&\lesssim n^{-1}R_n^2\sum_{\vec{\nu}\in\mathbb{V}}\left(1+\frac{\rho_{\vec{\nu}}}{\|\phi_{\vec{\nu}}\|_{L_2}^2}\right)^a(1+\lambda\rho_{\vec{\nu}})^{-2}\|\phi_{\vec{\nu}}\|_{L_2}^2\|\phi_{\vec{\nu}}\|_0^2\\
&\asymp n^{-1}R_n^2N_a(\lambda),
\end{aligned}
$$

where the fourth step is by (S27) and the last step is because of $\|\phi_{\vec{\nu}}\|_0=1$, $\|\phi_{\vec{\nu}}\|_{L_2}^2\asymp(1+\sum_{j=1}^p\nu_j^2)^{-1}$, $\rho_{\vec{\nu}}\asymp(1+\sum_{j=1}^p\nu_j^2)^{-1}\prod_{k=1}^d\nu_k^{2m}$, and $N_a(\lambda)$ is defined in Lemma S12. Hence, by Lemma S12, we complete the proof. ■

We now give an upper bound of $(\widehat{f}_n-\tilde{f}^*)$, which is another part of the stochastic error. Since $l_{n\lambda}(f)$ is a quadratic form of f, the Taylor expansion of $Dl_{n\lambda}(\widehat{f}_n)=0$ at $\bar{f}$ gives

$$
Dl_{n\lambda}(\bar{f})+D^2l_{n\lambda}(\bar{f})(\widehat{f}_n-\bar{f})=0,
$$

and by the definition of $\tilde{f}^*$ and G_λ, we have

$$
Dl_{n\lambda}(\bar{f})+D^2l_{\infty\lambda}(\bar{f})(\tilde{f}^*-\bar{f})=0.
$$

Thus, $G_\lambda(\widehat{f}_n-\tilde{f}^*)=\frac{1}{2}D^2l_\infty(\bar{f})(\widehat{f}_n-\bar{f})-\frac{1}{2}D^2l_n(\bar{f})(\widehat{f}_n-\bar{f})$, and

$$
\widehat{f}_n-\tilde{f}^*=G_\lambda^{-1}\left[\frac{1}{2}D^2l_\infty(\bar{f})(\widehat{f}_n-\bar{f})-\frac{1}{2}D^2l_n(\bar{f})(\widehat{f}_n-\bar{f})\right]. \qquad \text{(S28)}
$$

Lemma S5. *If* $n^{-1}\lambda^{-(2a+3/2m)}[\log(1/\lambda)]^{r-1} \to 0$ *and* $1/2m < a < (2m-3)/4m$*, we have for any* $0 \le c \le a + 1/m$*,*

$$\|\widehat{f}_n - \tilde{f}^*\|^2_{L_2(c)} = o_{\mathbb{P}}\left\{\|\tilde{f}^* - \bar{f}\|^2_{L_2(c)}\right\}.$$

Proof. A sufficient condition for this lemma is that for any $1/(2m) < a < (2m-3)/(4m)$ and $0 \le c \le a + 1/m$,

$$\begin{aligned}
&\|\widehat{f}_n - \tilde{f}^*\|^2_{L_2(c)} \\
&= \begin{cases} O_{\mathbb{P}}\left\{n^{-1}\lambda^{-(c+a+1/2m)}[\log(1/\lambda)]^{r\wedge(d-p)-1}\right\} \\ \qquad \cdot\|\widehat{f}_n - \bar{f}\|^2_{L_2(a+1/m)}, & \text{if } 0 \le p < d, \\ O_{\mathbb{P}}\left\{n^{-1}\lambda^{\frac{mr}{1-mr}\left(a+c+\frac{r-2}{2mr}\right)}\right\}\|\widehat{f}_n - \bar{f}\|^2_{L_2(a+1/m)}, & \text{if } p = d, r \ge 3, \\ O_{\mathbb{P}}\left\{n^{-1}\right\}\|\widehat{f}_n - \bar{f}\|_{L_2(a+1/m)}, & \text{if } p = d, r = 2, \\ O_{\mathbb{P}}\left\{n^{-1}\lambda^{\frac{1-2m(a+c)}{2m-2}}\right\}\|\widehat{f}_n - \bar{f}\|_{L_2(a+1/m)}, & \text{if } p = d, r = 1. \end{cases}
\end{aligned} \tag{S29}$$

This is because once (S29) established, by letting $c = a + 1/m$ and under the assumption that $n^{-1}\lambda^{-(2a+3/2m)}[\log(1/\lambda)]^{r-1} \to 0$, we have

$$\|\widehat{f}_n - \tilde{f}^*\|^2_{L_2(a+1/m)} = o_{\mathbb{P}}(1)\|\widehat{f}_n - \bar{f}\|^2_{L_2(a+1/m)}.$$

By the triangle inequality, we have $\|\tilde{f}^* - \bar{f}\|_{L_2(a+1/m)} \ge \|\widehat{f}_n - \bar{f}\|_{L_2(a+1/m)} - \|\widehat{f}_n - \tilde{f}^*\|_{L_2(a+1/m)} = [1 - o_{\mathbb{P}}(1)]\|\widehat{f}_n - \bar{f}\|_{L_2(a+1/m)}$, which implies $\|\widehat{f}_n - \bar{f}\|^2_{L_2(a+1/m)} = O_{\mathbb{P}}\{\|\tilde{f}^* - \bar{f}\|^2_{L_2(a+1/m)}\}$. Thus, by (S29) and Lemma S4, we complete the proof.

We now are in the position to prove (S29). For any $0 \le c \le a + 1/m$, by (S28), we have

$$\begin{aligned}
&\|\widehat{f}_n - \tilde{f}^*\|^2_{L_2(c)} \\
&\le \sum_{\vec{\boldsymbol{\nu}} \in \mathbb{V}} \left(1 + \frac{\rho_{\vec{\boldsymbol{\nu}}}}{\|\phi_{\vec{\boldsymbol{\nu}}}\|^2_{L_2}}\right)^c (1 + \lambda\rho_{\vec{\boldsymbol{\nu}}})^{-2}\|\phi_{\vec{\boldsymbol{\nu}}}\|^2_{L_2} \cdot \frac{1}{p+1} \cdot \\
&\quad \left\{\left[\frac{\sum_{i=1}^n (\widehat{f}_n - \bar{f})(\mathbf{t}_i^{(0)})\phi_{\vec{\boldsymbol{\nu}}}(\mathbf{t}_i^{(0)})}{n\sigma_0^2} - \frac{\int (\widehat{f}_n - \bar{f})(\mathbf{t})\phi_{\vec{\boldsymbol{\nu}}}(\mathbf{t})d\Pi^{(0)}(\mathbf{t})}{\sigma_0^2}\right]^2\right. \\
&\quad \left. + \sum_{j=1}^p \left[\frac{\sum_{i=1}^n \frac{\partial(\widehat{f}_n - \bar{f})}{\partial t_j}(\mathbf{t}_i^{(j)})\frac{\partial\phi_{\vec{\boldsymbol{\nu}}}}{\partial t_j}(\mathbf{t}_i^{(j)})}{n\sigma_j^2} - \frac{\int \frac{\partial(\widehat{f}_n - \bar{f})(\mathbf{t})}{\partial t_j}\frac{\partial\phi_{\vec{\boldsymbol{\nu}}}(\mathbf{t})}{\partial t_j}d\Pi^{(j)}(\mathbf{t})}{\sigma_j^2}\right]^2\right\}.
\end{aligned} \tag{S30}$$

Let $g_j(\mathbf{t}) = \frac{1}{\sigma_j^2}\frac{\partial(\widehat{f}_n - \bar{f})}{\partial t_j}\frac{\partial\phi_{\vec{\boldsymbol{\nu}}}}{\partial t_j}$ and $g_0(\mathbf{t}) = \frac{1}{\sigma_0^2}(\widehat{f}_n - \bar{f})\phi_{\vec{\boldsymbol{\nu}}}$. Hence, we can do the expansion on the basis $\{\phi_{\vec{\boldsymbol{\mu}}}\}_{\vec{\boldsymbol{\mu}} \in \mathbb{N}^d}$,

$$g_j(\mathbf{t}) = \sum_{\vec{\boldsymbol{\mu}} \in \mathbb{N}^d} Q^j_{\vec{\boldsymbol{\mu}}}\phi_{\vec{\boldsymbol{\mu}}}(\mathbf{t}), \qquad \text{where } Q^j_{\vec{\boldsymbol{\mu}}} = \langle g_j(\mathbf{t}), \phi_{\vec{\boldsymbol{\mu}}}(\mathbf{t})\rangle_0. \tag{S31}$$

Unlike (S14) with the multi-index $\vec{\boldsymbol{\nu}} \in \mathbb{V}$, we require $\vec{\boldsymbol{\mu}} \in \mathbb{N}^d$ in (S31) since now $g_j(\mathbf{t})$is a product function. By Cauchy-Schwarz inequality,

$$\begin{aligned}
&\left[\frac{1}{n\sigma_j^2}\sum_{i=1}^n \frac{\partial(\widehat{f}_n-\bar{f})}{\partial t_j}(\mathbf{t}_i^{(j)})\frac{\partial\phi_{\vec{\boldsymbol{\nu}}}}{\partial t_j}(\mathbf{t}_i^{(j)}) - \frac{1}{\sigma_j^2}\int \frac{\partial(\widehat{f}_n-\bar{f})(\mathbf{t})}{\partial t_j}\frac{\partial\phi_{\vec{\boldsymbol{\nu}}}(\mathbf{t})}{\partial t_j}\right]^2 \\
&= \left[\sum_{\vec{\boldsymbol{\mu}}\in\mathbb{N}^d} Q^j_{\vec{\boldsymbol{\mu}}}\left(\frac{1}{n}\sum_{i=1}^n \phi_{\vec{\boldsymbol{\mu}}}(\mathbf{t}_i^{(j)}) - \int \phi_{\vec{\boldsymbol{\mu}}}(\mathbf{t})\right)\right]^2 \\
&\le \left[\sum_{\vec{\boldsymbol{\mu}}\in\mathbb{N}^d} (Q^j_{\vec{\boldsymbol{\mu}}})^2\left(1+\frac{\rho_{\vec{\boldsymbol{\mu}}}}{\|\phi_{\vec{\boldsymbol{\mu}}}\|^2_{L_2}}\right)^a \|\phi_{\vec{\boldsymbol{\mu}}}\|^2_{L_2}\right] \\
&\cdot\left[\sum_{\vec{\boldsymbol{\mu}}\in\mathbb{N}^d}\left(1+\frac{\rho_{\vec{\boldsymbol{\mu}}}}{\|\phi_{\vec{\boldsymbol{\mu}}}\|^2_{L_2}}\right)^{-a}\|\phi_{\vec{\boldsymbol{\mu}}}\|^{-2}_{L_2}\left(\frac{1}{n}\sum_{i=1}^n \phi_{\vec{\boldsymbol{\mu}}}(\mathbf{t}_i^{(j)}) - \int \phi_{\vec{\boldsymbol{\mu}}}(\mathbf{t})\right)^2\right].
\end{aligned} \tag{S32}$$

By Lemma S13, if $a > 1/2m$, then the sum of the first part in the right-hand side of (S32) over $j = 0, 1, \ldots, p$ is bounded by

$$\begin{aligned}
&\sum_{j=0}^p\sum_{\vec{\boldsymbol{\mu}}\in\mathbb{N}^d}\left(1+\frac{\rho_{\vec{\boldsymbol{\mu}}}}{\|\phi_{\vec{\boldsymbol{\mu}}}\|^2_{L_2}}\right)^a\|\phi_{\vec{\boldsymbol{\mu}}}\|^2_{L_2}\left\langle\frac{\partial(\widehat{f}_n-\bar{f})}{\partial t_j}\frac{\partial\phi_{\vec{\boldsymbol{\nu}}}}{\partial t_j},\phi_{\vec{\boldsymbol{\mu}}}\right\rangle_0^2 \\
&\lesssim \|\widehat{f}_n-\bar{f}\|^2_{L_2(a+1/m)}\sum_{j=0}^p\sum_{\vec{\boldsymbol{\mu}}\in\mathbb{N}^d}\left(1+\frac{\rho_{\vec{\boldsymbol{\mu}}}}{\|\phi_{\vec{\boldsymbol{\mu}}}\|^2_{L_2}}\right)^a\|\phi_{\vec{\boldsymbol{\mu}}}\|^2_{L_2}\left\langle\frac{\partial\phi_{\vec{\boldsymbol{\nu}}}}{\partial t_j},\phi_{\vec{\boldsymbol{\mu}}}\right\rangle_0^2 \\
&\lesssim \|\widehat{f}_n-\bar{f}\|^2_{L_2(a+1/m)}\left(1+\frac{\rho_{\vec{\boldsymbol{\nu}}}}{\|\phi_{\vec{\boldsymbol{\nu}}}\|^2_{L_2}}\right)^a\|\phi_{\vec{\boldsymbol{\nu}}}\|^2_{L_2}\left(1+\sum_{j=1}^p\nu_j^2\right) \\
&\asymp \|\widehat{f}_n-\bar{f}\|^2_{L_2(a+1/m)}\left(1+\frac{\rho_{\vec{\boldsymbol{\nu}}}}{\|\phi_{\vec{\boldsymbol{\nu}}}\|^2_{L_2}}\right)^a.
\end{aligned} \tag{S33}$$

The second part on the right-hand side of (S32) can be bounded by

$$\begin{aligned}
&\mathbb{E}\left[\sum_{\vec{\boldsymbol{\mu}}\in\mathbb{N}^d}\left(1+\frac{\rho_{\vec{\boldsymbol{\mu}}}}{\|\phi_{\vec{\boldsymbol{\mu}}}\|^2_{L_2}}\right)^{-a}\|\phi_{\vec{\boldsymbol{\mu}}}\|^{-2}_{L_2}\left(\frac{1}{n}\sum_{i=1}^n \phi_{\vec{\boldsymbol{\mu}}}(\mathbf{t}_i^{(j)}) - \int \phi_{\vec{\boldsymbol{\mu}}}(\mathbf{t})\right)^2\right] \\
&\quad\le n^{-1}\sum_{\vec{\boldsymbol{\mu}}\in\mathbb{N}^d}\left(1+\frac{\rho_{\vec{\boldsymbol{\mu}}}}{\|\phi_{\vec{\boldsymbol{\mu}}}\|^2_{L_2}}\right)^{-a}\|\phi_{\vec{\boldsymbol{\mu}}}\|^{-2}_{L_2}\int\phi^2_{\vec{\boldsymbol{\mu}}}(\mathbf{t}) \\
&\quad\asymp n^{-1}\sum_{\vec{\boldsymbol{\mu}}\in\mathbb{N}^d}\left(1+\frac{\rho_{\vec{\boldsymbol{\mu}}}}{\|\phi_{\vec{\boldsymbol{\mu}}}\|^2_{L_2}}\right)^{-a} \lesssim n^{-1}\sum_{\vec{\boldsymbol{\mu}}\in\mathbb{N}^d}\mu_1^{-2ma}\cdots\mu_d^{-2ma} \\
&\quad\le n^{-1}\left(\sum_{\mu_1=1}^\infty \mu_1^{-2ma}\right)^d \asymp n^{-1},
\end{aligned} \tag{S34}$$

where the third step uses $\rho_{\vec{\mu}}/\|\phi_{\vec{\mu}}\|_{L_2}^2 \asymp \mu_1^{2m}\cdots\mu_d^{2m}$, and the fourth step holds for $a > 1/2m$. Combing (S33) and (S34), we have that for $a > 1/2m$,

$$\begin{aligned}&\sum_{j=0}^{p}\mathbb{E}\left[\sum_{\vec{\mu}\in\mathbb{N}^d} Q_{\vec{\mu}}^j\left(\frac{1}{n}\sum_{i=1}^{n}\phi_{\vec{\mu}}(\mathbf{t}_i^{(j)}) - \int\phi_{\vec{\mu}}(\mathbf{t})\right)\right]^2\\&\lesssim \frac{1}{n}\|\widehat{f}_n - \bar{f}\|_{L_2(a+1/m)}^2\left(1+\frac{\rho_{\vec{\nu}}}{\|\phi_{\vec{\nu}}\|_{L_2}^2}\right)^a.\end{aligned} \tag{S35}$$

Putting all together. Therefore, if $1/2m < a < (2m-3)/4m$ and $0 \le c \le a + 1/m$, (S30) and (S35) imply that

$$\mathbb{E}\|\widehat{f}_n - \tilde{f}^*\|_{L_2(c)}^2 \lesssim n^{-1}\|\widehat{f}_n - \bar{f}\|_{L_2(a+1/m)}^2 N_{a+c}(\lambda).$$

By Lemma S12 we complete the proof for (S29) and this lemma. ■

Finally, we combine (S12) and Lemmas S2–S5 to obtain the following proposition.

Proposition S1. *Under the conditions of Theorem 2 and assuming the distributions* $\Pi^{(0)}$ *and* $\Pi^{(j)}$*s are known. If* $1/2m < a < (2m-3)/4m$*, and* $n^{-1}\lambda^{-(2a+3/2m)}[\log(1/\lambda)]^{r-1} \to 0$*, then for any* $c \in [0, a+1/m]$*, the* $\widehat{f}_n$ *given by (9) satisfies, when* $0 \le p < d$*,*

$$\|\widehat{f}_n - f_0\|_{L_2(c)}^2 = O\left\{\min_{J(f)\le R_n^2}\|f-f_0\|_{L_2(c)}^2 + \lambda^{1-c}R_n^2\right\} + O_{\mathbb{P}}\left\{n^{-1}R_n^2\lambda^{-(c+1/2m)}[\log(1/\lambda)]^{r\wedge(d-p)-1}\right\},$$

and when $p = d$*,*

$$\begin{aligned}&\|\widehat{f}_n - f_0\|_{L_2(c)}^2\\&=\begin{cases} O\left\{\min_{J(f)\le R_n^2}\|f-f_0\|_{L_2(c)}^2 + \lambda^{\frac{(1-c)mr}{mr-1}}R_n^2\right\} + O_{\mathbb{P}}\left\{n^{-1}R_n^2\lambda^{\frac{mr}{1-mr}\left(c+\frac{r-2}{2mr}\right)}\right\} & \text{if } r\ge 3,\\ O\left\{\min_{J(f)\le R_n^2}\|f-f_0\|_{L_2(c)}^2 + \lambda^{\frac{2m}{2m-1}}R_n^2\right\} + O_{\mathbb{P}}\left\{n^{-1}R_n^2\log(1/\lambda)\right\} & \text{if } r=2, c=0,\\ O\left\{\min_{J(f)\le R_n^2}\|f-f_0\|_{L_2(c)}^2 + \lambda^{\frac{2(1-c)m}{2m-1}}R_n^2\right\} + O_{\mathbb{P}}\left\{n^{-1}R_n^2\lambda^{\frac{2mc}{1-2m}}\right\} & \text{if } r=2, c>0,\\ O\left\{\min_{J(f)\le R_n^2}\|f-f_0\|_{L_2(c)}^2 + \lambda^{\frac{(1-c)m}{m-1}}R_n^2\right\} + O_{\mathbb{P}}\left\{n^{-1}R_n^2\right\} & \text{if } r=1, c<\frac{1}{2m},\\ O\left\{\min_{J(f)\le R_n^2}\|f-f_0\|_{L_2(c)}^2 + \lambda^{\frac{2m-1}{2(m-1)}}R_n^2\right\} + O_{\mathbb{P}}\left\{n^{-1}R_n^2\log(1/\lambda)\right\} & \text{if } r=1, c=\frac{1}{2m},\\ O\left\{\min_{J(f)\le R_n^2}\|f-f_0\|_{L_2(c)}^2 + \lambda^{\frac{(1-c)m}{m-1}}R_n^2\right\} + O_{\mathbb{P}}\left\{n^{-1}R_n^2\lambda^{\frac{1-2mc}{2m-2}}\right\} & \text{if } r=1, c>\frac{1}{2m}.\end{cases}\end{aligned}$$

By Proposition S1, we can derive the convergence rates by the estimator $\widehat{f}_n$ defined by (9). In fact, for $p = d$ and $r \ge 3$, by letting $\lambda \asymp n^{-\frac{2mr-2}{(2m+1)r-2}}$, $a = 1/2m + \epsilon$ for some $\epsilon > 0$ and $c = 0$, we have that $n^{-1}\lambda^{-(2a+3/2m)}[\log(1/\lambda)]^{r-1} \to 0$ is equivalent to

$$-1 + \frac{5(mr-1)}{2m^2r + mr - 2m} < 0.$$

Thus, the conditions for Proposition S1 are satisfied. Similarly, we can verify that when $p = d$ and $r = 2$, $\lambda \asymp [n(\log n)]^{-(2m-1)/2m}$ satisfies the conditions for Proposition S1. When $p = d$ and $r = 1$, $\lambda \asymp n^{-(m-1)/m}$ satisfies the conditions for the above proposition. When $0 \le p \le d-r$, $\lambda \asymp [n(\log n)^{1-r}]^{-2m/(2m+1)}$ satisfies the conditions for the above Proposition, as well as when $d - r < p < d$ by letting $\lambda \asymp [n(\log n)^{1+p-d}]^{-2m/(2m+1)}$. This observation leads to the following theorem for $\widehat{f}_n$ in (9).

Theorem S3. *Assume that $\lambda_\nu \asymp \nu^{-2m}$ for some $m > 3/2$. Under the regression models (1) and (2) where f_0 follows the SS-ANOVA model (4) and $\|f\|_{\mathcal{H}} \le R_n$. Then under the general error structure (3), the estimator $\widehat{f}_n$ defined by (9) satisfies*

$$\lim_{C\to\infty} \limsup_{n\to\infty} \sup_{f_0\in\mathcal{H}} \mathbb{P}\left\{ \int_{\mathcal{X}^d} \left[\widehat{f}_n(\mathbf{t}) - f_0(\mathbf{t})\right]^2 d\mathbf{t} \le C \left(\left[n(\log n)^{1-(d-p)\wedge r}\right]^{-\frac{2m}{2m+1}} \mathbb{1}_{0\le p<d} \right.\right.$$
$$\left.\left. + \left[n^{-\frac{2mr}{(2m+1)r-2}} \mathbb{1}_{r\ge 3} + n^{-1}(\log n)^{r-1}\mathbb{1}_{r<3}\right] \mathbb{1}_{p=d} \right) \right\} = 1.$$

Here the tuning parameter λ in (14) is chosen by $\lambda \asymp \left[n(\log n)^{1-(d-p)\wedge r}\right]^{-2m/(2m+1)}$ when $0 \le p < d$, and $\lambda \asymp n^{-(2mr-2)/[(2m+1)r-2]}$ when $p = d, r \ge 3$, and $\lambda \asymp (n \log n)^{-(2m-1)/2m}$ when $p = d, r = 2$, and $\lambda \asymp n^{-(m-1)/m}$ when $p = d$, $r = 1$.

Finally, we approximate the estimator $\widehat{f}_n$ in (9) with the random feature estimator defined by (13),

$$\widehat{f}_n^{\mathrm{RF}} = \mathbf{\Psi}_{(p+1)d}(\mathbf{t})^\top \mathbf{c}_{(p+1)d}.$$

We have the following decomposition,

$$\widehat{f}_n^{\mathrm{RF}} - \widehat{f}_n = \underbrace{(S_s\widehat{C}_{s,\lambda}^{-1}\widehat{S}_s^* y - L_s L_{s,\lambda}^{-1} y)}_{\text{Error I}} + \underbrace{(L_s L_{s,\lambda}^{-1} y - L L_\lambda^{-1} y)}_{\text{Error II}}. \tag{S36}$$

Here the notations are similar to those in the Definition 2 of Rudi and Rosasco (2017). Specifically, let y be the vector of data, $y = (y_1^{(0)}, \dots, y_n^{(0)}, \dots, y_1^{(p)}, \dots, y_n^{(p)})^\top$. Moreover,

- The approximated kernel $K_s = \mathbf{\Psi}_{(p+1)d}(\mathbf{t})^\top(\mathbf{t})\mathbf{\Psi}_{(p+1)d}(\mathbf{t}')$.
- S_s: $(S_s\beta)(\cdot) = \mathbf{\Psi}_{(p+1)d}(\cdot)^\top \beta$.
- S_s^*: $S_s^* g = \frac{1}{\sqrt{s}} \int \mathbf{\Psi}_{(p+1)d}(\mathbf{t}) g(\mathbf{t}) d\mathbf{t}$.
- L_s: $(L_s g)(\cdot) = \int K_s(\cdot, \mathbf{t}) g(\mathbf{t}) d\mathbf{t}$.
- C_s: $C_s = \int \mathbf{\Psi}_{(p+1)d}(\mathbf{t}) \mathbf{\Psi}_{(p+1)d}(\mathbf{t})^\top d\mathbf{t}$.

- $\widehat{C}_s$: $\widehat{C}_s = \frac{1}{n}\sum_{i=1}^n \mathbf{\Psi}_{(p+1)d}(\mathbf{t}_i)\mathbf{\Psi}_{(p+1)d}(\mathbf{t}_i)^\top$.
- The random feature mapping estimator $\widehat{f}_n^{\mathrm{RF}} = S_s\widehat{C}_{s,\lambda}^{-1}\widehat{S}_s^* y$.

We analyze the two error terms in (S36) separately. For the Error I, note that, $L_sL_{s,\lambda}^{-1} = S_sC_{s,\lambda}^{-1}S_s^*$. Then,

$$\begin{aligned}
\text{Error I} &= S_s\widehat{C}_{s,\lambda}^{-1}\widehat{S}_s^* y - L_sL_{s,\lambda}^{-1}y \\
&= S_s\widehat{C}_{s,\lambda}^{-1}(\widehat{S}_s^* - S_s^*)y + S_s(\widehat{C}_{s,\lambda}^{-1} - C_{s,\lambda}^{-1})S_s^* y \\
&= S_s\widehat{C}_{s,\lambda}^{-1}(\widehat{S}_s^* - S_s^*)y + S_s\widehat{C}_{s,\lambda}^{-1}(C_{s,\lambda} - \widehat{C}_{s,\lambda})C_{s,\lambda}^{-1}S_s^* y \\
&= S_s\widehat{C}_{s,\lambda}^{-1}(\widehat{S}_s^* - S_s^*)y + (S_s\widehat{C}_{s,\lambda}^{-1}C_{s,\lambda}^{1/2})\left[C_{s,\lambda}^{-1/2}(C_{s,\lambda} - \widehat{C}_{s,\lambda})\right]C_{s,\lambda}^{-1}S_s^* y.
\end{aligned}$$

By Lemma 7 in Rudi and Rosasco (2017), we obtain that,

$$\|\text{Error I}\|_{L_2} \le O\left(\lambda^{-1/2}n^{-1} + n^{-1/2}\lambda^{-1/4m}\right).$$

By Lemma S4, both the term $\lambda^{-1/2}n^{-1}$ and the term $n^{-1/2}\lambda^{-1/4m}$ are dominated by $\|\tilde{f}^* - \bar{f}\|_{L_2}$. By Proposition S1,

$$\|\text{Error I}\|_{L_2} = O(\|\widehat{f}_n - f_0\|_{L_2}^2). \tag{S37}$$

For the Error II, by Lemma 8 and Equation (14) in Rudi and Rosasco (2017), we have

$$\|\text{Error II}\|_{L_2} = O\left(\sqrt{\frac{\log(1/\lambda)}{s}}\right),$$

By Proposition S1, and letting $s = O(n\log n)$, we have $\|\text{Error II}\|_{L_2} = O(n^{-1/2})$. Hence

$$\|\text{Error II}\|_{L_2} = O(\|\widehat{f}_n - f_0\|_{L_2}^2). \tag{S38}$$

By combining (S37) and (S38), we have $\|\widehat{f}_n^{\mathrm{RF}} - \widehat{f}_n\|_{L_2} = O(\|\widehat{f}_n - f_0\|_{L_2})$. By triangle inequality,

$$\begin{aligned}
\|\widehat{f}_n^{\mathrm{RF}} - f_0\|_{L_2} &= \|\{\widehat{f}_n^{\mathrm{RF}} - \widehat{f}_n\} + \{\widehat{f}_n - f_0\}\|_{L_2} \\
&\le \|\widehat{f}_n^{\mathrm{RF}} - \widehat{f}_n\|_{L_2} + \|\widehat{f}_n - f_0\|_{L_2} \\
&= O(\|\widehat{f}_n - f_0\|_{L_2}).
\end{aligned} \tag{S39}$$

Using Theorem S3 and (S39), we complete the proof of Theorem 3.

S4.4 Auxiliary Lemmas for Theorems 2 and 3

Lemma S6. *Suppose that* $\beta \geq 0$ *and* $0 < \alpha \leq 2$. *Then, as* $\Xi \to \infty$,

$$\int_{x_1 \cdots x_r \leq \Xi, x_k \geq 1} \prod_{k=1}^{r} x_k^{\beta} (x_1^{\alpha} + x_2^{\alpha} + \cdots + x_r^{\alpha})^{-1} dx_1 \cdots dx_r$$

$$\asymp \begin{cases} \Xi^{\beta+1-\alpha/r}, \ \textit{if}\ r \geq 3; \\ \log(\Xi), \ \textit{if}\ r = 2, \beta = \alpha/2 - 1; \quad \Xi^{\beta+1-\alpha/2}\ \textit{if}\ r = 2, \beta > \alpha/2 - 1; \\ 1, \ \textit{if}\ r = 1, \beta < \alpha - 1; \quad \log(\Xi)\ \textit{if}\ r = 1, \beta = \alpha - 1; \\ \Xi^{\beta-\alpha+1}\ \textit{if}\ r = 1, \beta > \alpha - 1. \end{cases}$$

Proof. By the symmetry of covariates,

$$\begin{aligned} &\int_{x_1 \cdots x_r \leq \Xi, x_k \geq 1} \prod_{k=1}^{r} x_k^{\beta} (x_1^{\alpha} + x_2^{\alpha} + \cdots + x_r^{\alpha})^{-1} dx_1 \cdots dx_r \\ \asymp &\int_{x_1 \cdots x_r \leq \Xi, x_1 \geq x_2 \geq \cdots \geq x_r \geq 1} \prod_{k=1}^{r} x_k^{\beta} (x_1^{\alpha} + x_2^{\alpha} + \cdots + x_r^{\alpha})^{-1} dx_r \cdots dx_1 \\ := &\mathcal{E}. \end{aligned}$$

First, we prove when $r \geq 3$, as $\Xi \to \infty$, we have

$$\mathcal{E} \lesssim \Xi^{\beta+1-\alpha/r}. \tag{S40}$$

For this, define the set $\mathcal{K} = \left\{0 \leq k \leq r-2 : \left(\frac{\Xi}{x_1 \cdots x_{r-k-1}}\right)^{1/(k+1)} \leq x_{r-k-1}\right\}$. If $\mathcal{K}$ is not empty, we denote the smallest element in $\mathcal{K}$ by k^*. Then $0 \leq k^* \leq r-2$. For any $(x_1, \ldots, x_r) \in \{(x_1, \ldots, x_r) : x_1 \cdots x_r \leq \Xi, x_1 \geq x_2 \geq \cdots \geq x_r \geq 1, x_r \leq x_{r-1} \leq \frac{\Xi}{x_1 \cdots x_{r-1}}\}$, we have

$$\begin{cases} 1 \leq x_{r-k} \leq x_{r-k-1} & \text{for } 0 \leq k \leq k^* - 1, \\ 1 \leq x_{r-k^*} \leq \left(\frac{\Xi}{x_1 \cdots x_{r-k^*-1}}\right)^{1/(k^*+1)} & \text{for } k = k^*, \\ x_{r-k} \geq \left(\frac{\Xi}{x_1 \cdots x_{r-k-1}}\right)^{1/(k+1)} & \text{for } k^* + 1 \leq k \leq r-2, \\ x_1 \geq \Xi^{1/r} & \text{for } k = r-1. \end{cases} \tag{S41}$$

Thus, as $\Xi \to \infty$,

$$
\begin{aligned}
\mathcal{E} &\lesssim \int_{x_1 \cdots x_r \le \Xi, x_1 \ge x_2 \ge \cdots \ge x_r \ge 1} \\
&\qquad \left\{ (x_1)^{\beta - \alpha/(r-1)} \cdots (x_{r-k^*-1})^{\beta - \alpha/(r-1)} \right\} x_{r-k^*}^{\beta} \\
&\qquad\quad \cdot \left\{ (x_{r-k^*+1})^{\beta - \alpha/(r-1)} \cdots (x_r)^{\beta - \alpha/(r-1)} \right\} d\mathbf{x} \\
&\asymp \int_{x_1 \cdots x_r \le \Xi, x_1 \ge x_2 \ge \cdots \ge x_r \ge 1} \\
&\qquad \left\{ (x_1)^{\beta - \alpha/(r-1)} \cdots (x_{r-k^*-1})^{\beta - \alpha/(r-1)} \right\} \\
&\qquad\quad \cdot (x_{r-k^*})^{[\beta + 1 - \alpha/(r-1)]k^* + \beta} dx_{r-k^*} dx_{r-k^*-1} \cdots dx_1 \\
&\asymp \int_{x_1 \cdots x_r \le \Xi, x_1 \ge x_2 \ge \cdots \ge x_r \ge 1} \\
&\qquad \left\{ (x_1)^{-1 - \alpha/[(r-1)(k^*+1)]} \cdots (x_{r-k^*-1})^{-1 - \alpha/[(r-1)(k^*+1)]} \right\} \\
&\qquad\quad \cdot \Xi^{\beta + 1 - \alpha k^*/[(r-1)(k^*+1)]} dx_{r-k^*-1} \cdots dx_1 \\
&= \Xi^{\beta + 1 - \alpha/r},
\end{aligned}
\tag{S42}
$$

where the first step uses $x_{r-k^*} \ge 1$ and Lemma S14, the second step uses $x_{r-k} \le x_{r-k-1}$ for all $k \le k^* - 1$ in (S41), the third step uses the upper bound on x_{r-k^*} in (S41), the fourth step uses the lowers bounds on x_{r-k} for all $k^* + 1 \le k \le r - 2$ in (S41). If $\mathcal{K}$ is empty, then for any $(x_1, \ldots, x_r) \in \{(x_1, \ldots, x_r) : x_1 \cdots x_r \le \Xi, x_1 \ge x_2 \ge \cdots \ge x_r \ge 1, x_r \le x_{r-1} \le \Xi/(x_1 \cdots x_{r-1})\}$, it satisfies

$$
1 \le x_k \le x_{k-1} \text{ for any } 2 \le k \le r, \quad \text{and} \quad 1 \le x_1 \le \Xi^{1/r}.
$$

Thus, as $\Xi \to \infty$,

$$
\begin{aligned}
\mathcal{E} &= \int_1^{\Xi^{1/r}} \cdots \int_1^{x_{r-2}} \int_1^{x_{r-1}} \\
&\qquad \prod_{k=1}^{r} x_k^{\beta} (x_1^{\alpha} + x_2^{\alpha} + \cdots + x_{r-1}^{\alpha} + x_r^{\alpha})^{-1} dx_r dx_{r-1} \cdots dx_1 \\
&\lesssim \int_1^{\Xi^{1/r}} \cdots \int_1^{x_{r-2}} \int_1^{x_{r-1}} \\
&\qquad x_1^{\beta - \alpha/r} \cdots x_{r-1}^{\beta - \alpha/r} x_r^{\beta - \alpha/r} dx_r dx_{r-1} \cdots dx_1 \asymp \Xi^{\beta + 1 - \alpha/r}.
\end{aligned}
\tag{S43}
$$

Combining (S42) and (S43) completes the proof for (S40).

On the other hand, when $r \geq 3$ and as $\Xi \to \infty$,

$$\begin{aligned}\mathcal{E} &\geq \int_1^{\Xi^{1/r}} \cdots \int_1^{x_{r-2}} \int_1^{x_{r-1}} \\ &\qquad \prod_{k=1}^{r} x_k^{\beta} (x_1^{\alpha} + \cdots + x_{r-1}^{\alpha} + x_r^{\alpha})^{-1} dx_r dx_{r-1} \cdots dx_1 \\ &\geq \int_1^{\Xi^{1/r}} \cdots \int_1^{x_{r-2}} \int_1^{x_{r-1}} \\ &\qquad \prod_{k=1}^{r} x_k^{\beta} \cdot r^{-1} x_1^{-\alpha} dx_r dx_{r-1} \cdots dx_1 \asymp \Xi^{\beta+1-\alpha/r}.\end{aligned} \tag{S44}$$

Therefore, combining (S40) and (S44) completes the proof of the lemma for $r \geq 3$.

Then we consider for $r = 2$. For $0 < \alpha \leq 2$,

$$\begin{aligned}\mathcal{E} &\leq 2 \int_1^{\sqrt{\Xi}} \int_1^{x_1} x_1^{\beta-\alpha} x_2^{\beta} dx_2 dx_1 + 2 \int_{\sqrt{\Xi}}^{\Xi} \int_1^{\Xi/x_1} x_1^{\beta-\alpha} x_2^{\beta} dx_2 dx_1 \\ &\asymp \begin{cases} \log(\Xi) & \text{when } 2\beta + 2 - \alpha = 0 \\ \Xi^{\beta+1-\alpha/2} & \text{when } 2\beta + 2 - \alpha > 0 \end{cases} \quad \text{as } \Xi \to \infty.\end{aligned} \tag{S45}$$

On the other hand, we have

$$\begin{aligned}\mathcal{E} &\geq \int_1^{\sqrt{\Xi}} \int_1^{x_1} x_1^{\beta} x_2^{\beta} (x_1^{\alpha} + x_2^{\alpha})^{-1} dx_2 dx_1 \\ &\geq 2^{-1} \int_1^{\sqrt{\Xi}} \int_1^{x_1} x_1^{\beta-2} x_2^{\beta} dx_2 dx_1 \\ &\asymp \begin{cases} \log(\Xi) & \text{when } 2\beta + 2 - \alpha = 0 \\ \Xi^{m} & \text{when } 2\beta + 2 - \alpha > 0 \end{cases} \quad \text{as } \Xi \to \infty.\end{aligned} \tag{S46}$$

Combining (S45) and (S46) completes the proof of the lemma for $r = 2$.

Finally, we consider for $r = 1$. Note that $\int_1^{\Xi} x_1^{\beta} x_1^{-\alpha} dx_1 \asymp 1$ when $0 \leq \beta < \alpha - 1$, and $\int_1^{\Xi} x_1^{\beta} x_1^{-\alpha} dx_1 \asymp \log(\Xi)$ when $\beta = \alpha - 1$, and $\int_1^{\Xi} x_1^{\beta} x_1^{-\alpha} dx_1 \asymp \Xi^{\beta-\alpha+1}$ when $\beta > \alpha - 1$. This completes the proof. ■

Lemma S7. *The norm $\|\cdot\|_R$ is equivalent to $\|\cdot\|_{\mathcal{H}}$ in $\mathcal{H}$.*

Proof. Observe that for any $g \in \mathcal{H}$, by the assumption that $\Pi^{(0)}$ and $\Pi^{(j)}$s are bounded

away from 0 and infinity, we have

$$\begin{aligned}&\frac{1}{p+1}\left[\frac{1}{\sigma_0^2}\int g^2(\mathbf{t})\Pi^{(0)}(\mathbf{t})+\sum_{j=1}^{p}\frac{1}{\sigma_j^2}\int\left\{\frac{\partial g(\mathbf{t})}{\partial t_j}\right\}^2\Pi^{(j)}(\mathbf{t})\right]\\&\leq c_1\left[\int g^2(\mathbf{t})+\sum_{j=1}^{p}\int\left\{\frac{\partial g(\mathbf{t})}{\partial t_j}\right\}^2\right]\leq c_2\cdot c_K^{2d}\|g\|_{\mathcal{H}}^2,\end{aligned}$$

for some constant c_1 and c_2, where the last step is by Lemma S11. Hence

$$\|g\|_R^2\leq(c_2c_K^{2d}+1)\|g\|_{\mathcal{H}}^2. \tag{S47}$$

On the other hand, for any $g\in\mathcal{H}$ we can do the orthogonal decomposition $g=g^0+g^1$ where $\langle g^0,g^1\rangle_{\mathcal{H}}=0$, g^0 is in the null space of $J(\cdot)$ and g^1 is in the orthogonal space of the null space of $J(\cdot)$ in $\mathcal{H}$. Since the null space of $J(\cdot)$ has a finite basis that forms a positive definite kernel matrix, we assume the minimal eigenvalue of the kernel matrix is $\mu'_{\min}>0$. Then there exists a constant $c_3>0$ such that

$$\|g^0\|_R^2\geq c_3\|g^0\|_{L_2}^2\geq c_3\mu'_{\min}\|g^0\|_{\mathcal{H}}^2. \tag{S48}$$

For g^1, we have $\|g^1\|_R^2\geq J(g^1)=\|g^1\|_{\mathcal{H}}^2$. Thus, for any $g\in\mathcal{H}$,

$$\begin{aligned}\|g\|_R^2&\geq c_3\int\left(g^0+g^1\right)^2+\|g^1\|_{\mathcal{H}}^2\\&\geq c_3\left\{\|g^0\|_{L_2}^2+\frac{1+c_3}{c_3}\|g^1\|_{L_2}^2-2\|g^0\|_{L_2}\|g^1\|_{L_2}\right\}\\&\geq\frac{c_3}{1+c_3}\|g^0\|_{L_2}^2,\end{aligned}$$

where the second inequality is by $\|g^1\|_{\mathcal{H}}^2\geq\|g^1\|_{L_2}^2$. By (S48), we obtain $\|g\|_R^2\geq(1+c_3)^{-1}c_3\mu'_{\min}\|g^0\|_{\mathcal{H}}^2$. Together with $\|g\|_R^2\geq J(g^1)=\|g^1\|_{\mathcal{H}}^2$, we have

$$\|g\|_R^2\geq\left(1+\frac{1+c_3}{c_3\mu'_{\min}}\right)^{-1}\|g\|_{\mathcal{H}}^2. \tag{S49}$$

Combining (S47) and (S49) completes the proof. ■

Lemma S8 (Inverse transformation)**.** *Suppose that designs* $\mathbf{t}^{(j)}$, $j=0,\ldots,p$ *are independently drawn from known distributions* $\Pi^{(j)}$ *supported in* $\mathcal{X}^d$*. Then, there exists a linear transformation to data* $(\mathbf{t}^{(j)},Y^{(j)})$ *such that transformed design points* $\mathbf{x}^{(j)}$*s are independently uniformly distributed on* $\mathcal{X}^d$*.*

Proof. First, we consider function and derivative data sharing a common design, i.e., $\mathbf{t}_i^{(j)} = \mathbf{t}_i^{(k)}$, $\forall 1 \le i \le n, 0 \le j < k \le p$. Write $\mathbf{t}^{(j)} = (t_1^{(j)}, \dots, t_d^{(j)}) \in \mathcal{X}^d$. We allow covariates of $\mathbf{t}^{(j)}$ can be correlated; that is, the density of $\mathbf{t}^{(j)}$ is decomposed as:

$$d\Pi^{(j)}(t_1, \dots, t_d) = d\Pi_d^{(j)}(t_d) d\Pi_{d-1}^{(j)}(t_{d-1}|t_d) \cdots d\Pi_1^{(j)}(t_1|t_d, t_{d-1}, \dots, t_2).$$

Now let

$$x_d^{(j)} = \Pi_d^{(j)}(t_d^{(j)}), \quad x_{d-1}^{(j)} = \Pi_{d-1}^{(j)}(t_{d-1}^{(j)}|t_d^{(j)}), \dots, \quad x_1^{(j)} = \Pi_1^{(j)}(t_1^{(j)}|t_d^{(j)}, t_{d-1}^{(j)} \dots, t_2^{(j)}).$$

Then, $\mathbf{x}^{(j)} = (x_1^{(j)}, x_2^{(j)}, \dots, x_d^{(j)})$ is uniformly distributed on $\mathcal{X}^d$. Define that

$$\begin{aligned} &h(x_1, x_2, \dots, x_d) \\ &= f(\{\Pi_1^{(j)}\}^{-1}(x_1|x_d, \dots, x_2), \{\Pi_2^{(j)}\}^{-1}(x_2|x_d, \dots, x_3), \dots, \{\Pi_d^{(j)}\}^{-1}(x_d)). \end{aligned}$$

Thus,

$$\frac{\partial h(\mathbf{x})}{\partial x_j} = \sum_{k=1}^{j} \frac{\partial f(\mathbf{t})}{\partial t_k} \cdot \frac{\partial t_k}{\partial x_j} = \sum_{k=1}^{j-1} \frac{\partial f}{\partial t_k} \cdot \frac{\partial t_k}{\partial x_j} + \frac{\partial f}{\partial t_j} \cdot \frac{1}{d\Pi_j^{(j)}(t_j|t_d, \dots, t_{j+1})}.$$

With the design $\mathbf{x}^{(j)}$ defined, we transform the responses $Y^{(j)}$s to $Z^{(j)}$s by letting $Z^{(0)} = Y^{(0)}$ and for any $j = 1, \dots, p$,

$$Z^{(j)} = \sum_{k=1}^{j-1} Y^{(k)} \frac{\partial t_k^{(j)}(x_d^{(j)}, x_{d-1}^{(j)} \dots, x_k^{(j)})}{\partial x_j} + \frac{Y^{(j)}}{d\Pi_j^{(j)}(t_j^{(j)}|t_d^{(j)}, \dots, t_{j+1}^{(j)})}.$$

Write

$$\tilde{\sigma}_j^2 = \sum_{k=1}^{j-1} \sigma_k^2 \left[\frac{\partial t_k^{(j)}}{\partial x_j}(x_d^{(j)}, x_{d-1}^{(j)}, \dots, x_k^{(j)}) \right]^2 + \frac{\sigma_j^2}{[d\Pi_j^{(j)}(t_j^{(j)}|t_d^{(j)}, \dots, t_{j+1}^{(j)})]^2}.$$

Then it is clear that $Z^{(j)} = \partial h / \partial x_j(\mathbf{x}^{(j)}) + \widetilde{\epsilon^{(j)}}$, where the errors $\widetilde{\epsilon^{(j)}}$s are independent and centered noises with variance $\tilde{\sigma}_j^2$s.

Second, we consider that not all types of function observations and partial derivatives data share a common design, i.e., $\exists 0 \le j \ne k \le p$ and $1 \le i \le n$ such that $\mathbf{t}_i^{(j)} \ne \mathbf{t}_i^{(k)}$. We require the covariates of each $\mathbf{t}^{(j)}$ are independent; that is, the density of $\mathbf{t}^{(j)}$ can be decomposed as:

$$d\Pi^{(j)}(t_1, \dots, t_d) = d\Pi_1^{(j)}(t_1) d\Pi_2^{(j)}(t_2) \cdots d\Pi_d^{(j)}(t_d)$$

Now let

$$x_1^{(j)} = \Pi_1^{(j)}(t_1^{(j)}), \quad x_2^{(j)} = \Pi_2^{(j)}(t_2^{(j)}), \quad \dots, \quad x_d^{(j)} = \Pi_d^{(j)}(t_d^{(j)}).$$

Then $\mathbf{x}^{(j)} = (x_1^{(j)}, x_2^{(j)}, \ldots, x_d^{(j)})$ is uniformly distributed on $\mathcal{X}^d$. Define the function

$$h(x_1, \ldots, x_d) = f(\{\Pi_1^{(j)}\}^{-1}(x_1), \{\Pi_2^{(j)}\}^{-1}(x_2), \ldots, \{\Pi_d^{(j)}\}^{-1}(x_d)).$$

Thus, we have

$$\frac{\partial h(\mathbf{x})}{\partial x_j} = \frac{\partial f(\mathbf{t})}{\partial t_j} \cdot \frac{\partial t_j(x_j)}{\partial x_j} = \frac{\partial f(\mathbf{t})}{\partial t_j} \cdot \frac{1}{d\Pi_j^{(j)}(t_j)}.$$

Correspondingly, the responses $Y^{(j)}$ is transformed to $Z^{(j)}$, $0 \le j \le p$, by letting $Z^{(0)} = Y^{(0)}$ and $Z^{(j)} = Y^{(j)}/d\Pi_j^{(j)}(t_j^{(j)})$ for $1 \le j \le d$, and write the transformed variance $\tilde{\sigma}_j^2 = \sigma_j^2/[d\Pi_j^{(j)}(t_j^{(j)})]^2$. ■

Lemma S9. *Suppose that f_0 follows the SS-ANOVA model in (4), defined on $\mathcal{X}^d \equiv [0,1]^d$. Then, there exists a periodic function $\tilde{f}_0$ on the expanded domain $[0, 1+\delta]^d$ for any $\delta > 0$ such that $\tilde{f}_0(\mathbf{t}) \equiv f_0(\mathbf{t})$ for $\mathbf{t} \in \mathcal{X}^d$, and $\tilde{f}_0$ maintains the same order of smoothness as f_0, in the sense that $\tilde{f}_0$ follows the same RKHS in (5), defined on $[0, 1+\delta]^d$.*

Proof. The construction of the periodic function consists of four main steps.

Step 1: We show that when $\lambda_\nu \asymp \nu^{-2m}$, the m-th order Sobolev space on $\mathcal{X}$ can be embedded into the RKHS $\mathcal{H}_1$. Specifically, let $\mathcal{W}_2^m(\mathcal{X})$ denote the Sobolev space of order m, consisting of functions whose derivatives up to order $m-1$ are absolutely continuous and whose m-th derivative is square-integrable:

$$\mathcal{W}_2^m(\mathcal{X}) = \Big\{ g : \mathcal{X} \to \mathbb{R}\ g, dg/dt, \ldots, d^{m-1}g/dt^{m-1} \text{ are absolutely continuous, and } d^m g/dt^{(m)} \in L_2 \Big\}.$$

There are many possible norms that can be quipped with $\mathcal{W}_2^m$ to make it a RKHS. For example, it can be endowed with the norm,

$$\|g\|_{\mathcal{W}_2^m}^2 = \sum_{q=0}^{m-1} \left(\int g^{(q)} \right)^2 + \int \left(g^{(m)} \right)^2.$$

Following the results in Chapter 2 of Wahba (1990), the eigenvalues of the associated kernel decay at a rate of $\lambda_\nu \asymp \nu^{-2m}$ for $\nu \ge 1$.

Step 2: For any $f_{0j} \in \mathcal{H}_1$ on $\mathcal{X}$, $j = 1, \ldots, d$, we construct the function g_j as,

$$g_j(t_j) = \sum_{k=0}^{2m+1} c_{jk} t_j^k, \text{ for } t_j \in [1, 1+\delta], \tag{S50}$$

where the coefficients $\{c_{jk}\}_{k=0}^{2m+1}$ are computed by satisfying the linear system:

$$g_j^{(q)}(1) = f_{0j}^{(q)}(1) \text{ and } g_j^{(q)}(1+\delta) = f_{0j}^{(q)}(0), \quad \forall q = 0, 1, \dots, m. \tag{S51}$$

Since the linear system (S51) has $2m+2$ equations and the function g_j in (S50) has $2m+2$ free coefficients $\{c_{jk}\}_{k=0}^{2m+1}$, there is a unique solution. We define the extended function as,

$$\tilde{f}_{0j}(t_j) = \begin{cases} f_{0j}(t_j), & t_j \in [0,1], \\ g_j(t_j), & t_j \in [1, 1+\delta], \end{cases}$$

where g_j is the $(2m+1)$-th order polynomial defined in (S50). Since g_j is continuous and has $m-1$ absolutely continuous derivatives, together with the property that the m-th derivative of g_j is in L_2, we know that $\tilde{f}_{0j}(t_j) \in \mathcal{W}_2^m([0, 1+\delta])$. By the result in Step 1, the m-th order Sobolev space $\mathcal{W}_2^m(\mathcal{X})$ can be embedded to the RKHS $\mathcal{H}_1$. Hence $\tilde{f}_{0j}(t_j)$ follows the same RKHS as $f_{0j}(t_j)$ with the expanded domain on $[0, 1+\delta]$.

Step 3: For any $f_{0j_1j_2\cdots j_r} \in \mathcal{H}_1 \otimes \mathcal{H}_1 \otimes \cdots \otimes \mathcal{H}_1$, $1 \le j_1 < j_2 < \cdots < j_r \le d$ and $1 \le r \le d$, there exists a finite integer s and functions $f_{0j_1\nu}, f_{0j_2\nu}, \dots, f_{0j_r\nu} \in \mathcal{H}_1$ for $\nu = 1, \dots, s$, such that

$$f_{0j_1j_2\cdots j_r}(t_{j_1}, t_{j_2}, \dots, t_{j_r}) = \sum_{\nu=1}^{s} f_{0j_1\nu}(t_{j_1}) f_{0j_2\nu}(t_{j_2}) \cdots f_{0j_r\nu}(t_{j_r}).$$

By the construction in Step 2, we can find $g_{j\nu}(t_{j_1}) = \sum_{k=0}^{2m+1} c_{j\nu k} t_j^k$ for $t_j \in [1, 1+\delta]$ and $j = j_1, j_2, \dots, j_r$, such that,

$$g_{j\nu}^{(q)}(1) = f_{0j\nu}^{(q)}(1) \text{ and } g_{j\nu}^{(q)}(1+\delta) = f_{0j\nu}^{(q)}(0), \quad \forall q = 0, 1, \dots, m, \tag{S52}$$

Since the linear system (S52) has $(2m+2)$ equations and the function $g_{j\nu}(t_{j_1}) = \sum_{k=0}^{2m+1} c_{j\nu k} t_j^k$ has $(2m+2)$ free coefficients $\{c_{j\nu k}\}_{k=0}^{2m+1}$, there is a unique solution. We define the extended function as,

$$\tilde{f}_{0j_1j_2\cdots j_r}(t_{j_1}, t_{j_2}, \dots, t_{j_r}) = \sum_{\nu=1}^{s} h_{j_1\nu}(t_{j_1}) h_{j_2\nu}(t_{j_2}) \cdots h_{j_r\nu}(t_{j_r}),$$

for any $(t_{j_1}, t_{j_2}, \dots, t_{j_r}) \in [0, 1+\delta]^r$, where for any $j = j_1, j_2, \dots, j_r$, the function $h_{j\nu}$ is defined as,

$$h_{j\nu}(t_j) = \begin{cases} f_{0j\nu}(t_j) & t_j \in [0,1], \\ g_{j\nu}(t_j) & t_j \in [1, 1+\delta], \end{cases}$$

and $g_{j\nu}(t_{j_1}) = \sum_{k=0}^{2m+1} c_{j\nu k} t_j^k$ is the $(2m+1)$-th order polynomial. Since $g_{j\nu}$ is continuous and has $(m-1)$ absolutely continuous derivatives, together with the property that the m-th

derivative of $g_{j\nu}$ is in L_2, we know that $\tilde{f}_{0j_1j_2\cdots j_r}(t_{j_1}, t_{j_2}, \ldots, t_{j_r}) \in \mathcal{W}_2^m([0, 1+\delta]) \otimes \mathcal{W}_2^m([0, 1+\delta]) \otimes \cdots \otimes \mathcal{W}_2^m([0, 1+\delta])$. By the result in Step 1, the m-th order Sobolev space $\mathcal{W}_2^m(\mathcal{X})$ can be embedded to the RKHS $\mathcal{H}_1$. Hence $\tilde{f}_{0j_1j_2\cdots j_r}(t_{j_1}, t_{j_2}, \ldots, t_{j_r})$ follows the same RKHS as $f_{0j_1j_2\cdots j_r}$ with the expanded domain on $[0, 1+\delta]^r$.

Step 4: For f_0 follows the SS-ANOVA model (4) on $\mathcal{X}^d$, we can define the function $\tilde{f}_0(\mathbf{t})$ that extends f_0 from $\mathcal{X}^d$ to $[0, 1+\delta]^d$ for any $\delta > 0$. Specifically, let

$$\tilde{f}_0(\mathbf{t}) = \text{constant} + \sum_{j=1}^{d} \tilde{f}_{0j}(t_j) + \cdots + \sum_{1 \le j_1 < j_2 < \cdots < j_r \le d} \tilde{f}_{0j_1j_2\cdots j_r}(t_{j_1}, t_{j_2}, \ldots, t_{j_r}).$$

By the construction in Steps 2 and 3, we have that $\tilde{f}_0(\mathbf{t}) = f_0(\mathbf{t})$ for $\mathbf{t} \in \mathcal{X}^d$, which implies that $\tilde{f}_0(\mathbf{t})$ coincides with $f_0(\mathbf{t})$ on the original domain $\mathcal{X}^d$. Moreover, $\tilde{f}_0(\mathbf{t})$ the same order of smoothness as $f_0(\mathbf{t})$ in the sense that $\tilde{f}_0(\mathbf{t})$ follows the same RKHS in (5) defined on $[0, 1+\delta]^d$. Hence, the eigenvalue decay rate of the RKHS for $\tilde{f}_0(\mathbf{t})$ is the same as that of the RKHS for $f_0(\mathbf{t})$. Finally, by (S51) and (S52), we have that for any $j = 1, \ldots, d$ and $(t_1, \ldots, t_{j-1}, t_{j+1}, \ldots, t_d) \in [0, 1+\delta]^{d-1}$,

$$\tilde{f}_0(t_1, \ldots, t_{j-1}, 0, t_{j+1}, \ldots, t_d) = \tilde{f}_0(t_1, \ldots, t_{j-1}, 1+\delta, t_{j+1}, \ldots, t_d),$$

which shows that the extended function $\tilde{f}_0$ has a periodic boundary on the expanded domain $[0, 1+\delta]^d$ for any $\delta > 0$. This completes the proof. ■

Lemma S10. *Suppose that f_0 follows the SS-ANOVA model in (4), defined on $\mathcal{X}^d \equiv [0,1]^d$, and the periodic function $\tilde{f}_0$ is constructed in Lemma S9, defined on $[0, 1+\delta]^d$. Then, if $\delta > 0$ and for any estimator $\widehat{f}_n$ on $[0, 1+\delta]^d$, we have that $\int_{\mathcal{X}^d}[\widehat{f}_n(\mathbf{t}) - f_0(\mathbf{t})]^2 d\mathbf{t} \le \int_{[0,1+\delta]^d}[\widehat{f}_n(\mathbf{t}) - \tilde{f}_0(\mathbf{t})]^2 d\mathbf{t}$.*

Proof. We decompose the estimation error of $\tilde{f}_0$ as follows:

$$\begin{aligned}
&\int_{[0,1+\delta]^d} \left[\widehat{f}_n(\mathbf{t}) - \tilde{f}_0(\mathbf{t})\right]^2 d\mathbf{t} \\
&= \int_{\mathcal{X}^d} \left[\widehat{f}_n(\mathbf{t}) - \tilde{f}_0(\mathbf{t})\right]^2 d\mathbf{t} + \int_{[0,1+\delta]^d \setminus \mathcal{X}^d} \left[\widehat{f}_n(\mathbf{t}) - \tilde{f}_0(\mathbf{t})\right]^2 d\mathbf{t} \\
&= \int_{\mathcal{X}^d} \left[\widehat{f}_n(\mathbf{t}) - f_0(\mathbf{t})\right]^2 d\mathbf{t} + \int_{[0,1+\delta]^d \setminus \mathcal{X}^d} \left[\widehat{f}_n(\mathbf{t}) - \tilde{f}_0(\mathbf{t})\right]^2 d\mathbf{t} \\
&\ge \int_{\mathcal{X}^d} \left[\widehat{f}_n(\mathbf{t}) - f_0(\mathbf{t})\right]^2 d\mathbf{t},
\end{aligned}$$

where the second step uses the property that $\tilde{f}_0(\mathbf{t}) \equiv f_0(\mathbf{t})$ for $\mathbf{t} \in \mathcal{X}^d$. ■

Lemma S11. *For any $g \in \mathcal{H}$, there exists a constant c_K which is independent of g such that*

$$\sup_{\mathbf{t} \in \mathcal{X}^d} |g(\mathbf{t})| \le c_K^d \|g\|_{\mathcal{H}},$$

and

$$\sup_{\mathbf{t} \in \mathcal{X}^d} \left| \frac{\partial g(\mathbf{t})}{\partial t_j} \right| \le c_K^d \|g\|_{\mathcal{H}}, \quad \forall 1 \le j \le d.$$

Proof. Since we assume that K is continuous in the compact domain $\mathcal{X}$ and satisfies (7), there exists some constant c_K such that

$$\sup_{t \in \mathcal{X}} |K(t,t)| \le c_K \quad \text{and} \quad \sup_{t \in \mathcal{X}} \left| \frac{\partial^2 K(t,t)}{\partial t \partial t'} \right| \le c_K.$$

This implies for any $\mathbf{t} \in \mathcal{X}^d$,

$$\left\| \frac{\partial K_d(\mathbf{t}, \cdot)}{\partial t_j} \right\|_{\mathcal{H}}^2 = \left| \frac{\partial^2 K(t_j, t_j)}{\partial t_j \partial t_j'} \right| \prod_{l \ne j} |K(t_l, t_l)| \le c_K^d.$$

Thus, for any $g \in \mathcal{H}$, by the Cauchy-Schwarz inequality,

$$\sup_{\mathbf{t} \in \mathcal{X}^d} \left| \frac{\partial g(\mathbf{t})}{\partial t_j} \right| \le \sup_{\mathbf{t} \in \mathcal{X}^d} \left\| \frac{\partial K_d(\mathbf{t}, \cdot)}{\partial t_j} \right\|_{\mathcal{H}} \|g\|_{\mathcal{H}} \le c_K^d \|g\|_{\mathcal{H}}, \quad \forall 1 \le j \le d.$$

Similarly, we can show that $\sup_{\mathbf{t}} |g(\mathbf{t})| \le c_K^d \|g\|_{\mathcal{H}}$.

■

Lemma S12. *Recall that $\mathbb{V}$ as a family of multi-index $\vec{\boldsymbol{\nu}}$ is defined in (S3). We let*

$$N_a(\lambda) = \sum_{\vec{\boldsymbol{\nu}} \in \mathbb{V}} \frac{\left(\prod_{k=1}^d \nu_k^{2m} \right)^a \left(1 + \sum_{j=1}^p \nu_j^2 \right)}{\left(1 + \sum_{j=1}^p \nu_j^2 + \lambda \prod_{k=1}^d \nu_k^{2m} \right)^2}. \tag{S53}$$

Then, when $0 \le p < d$, we have for any $0 \le a < 1 - 1/2m$,

$$N_a(\lambda) = O\left\{ \lambda^{-a-1/2m} \left[\log(1/\lambda) \right]^{(d-p) \wedge r - 1} \right\},$$

and when $p = d$, we have for any $0 \le a \le 1$,

$$N_a(\lambda) = \begin{cases} O\left\{ \lambda^{\frac{mr}{1-mr}\left(a + \frac{r-2}{2mr}\right)} \right\}, \ \textit{if } r \ge 3; \\ O\{\log(1/\lambda)\}, \ \textit{if } r = 2, a = 0; \quad O\{1\}, \ \textit{if } r = 2, 0 < a \le 1; \\ O\{1\}, \ \textit{if } r = 1, a < \frac{1}{2m}; \quad O\{\log(1/\lambda)\}, \ \textit{if } r = 1, a = \frac{1}{2m}; \\ O\left\{ \lambda^{\frac{1-2ma}{2m-2}} \right\}, \ \textit{if } r = 1, a > \frac{1}{2m}. \end{cases}$$

Proof. We will discuss three separate cases for $0 \le p \le d-r$, $d-r < p < d$ and $p = d$.

First, consider $0 \le p \le d-r$. Since $\boldsymbol{\vec{\nu}} \in \mathbb{V}$, there are at most r of $\nu_1, \ldots, \nu_d$ not equal to 1, which implies that the number of combinations of non-1 indices being summed in (S53) is no greater than $C_d^1 + C_d^2 + \cdots + C_d^r < \infty$. Due to the appearance of $(1 + \sum_{j=1}^p \nu_j^2)$ in the denominator of (S53), the largest terms of the summation (S53) over $\boldsymbol{\vec{\nu}} \in \mathbb{V}$ correspond to the combinations of r indices whereas few $\nu_1, \ldots, \nu_p$ being summed as possible, which is the indices $\boldsymbol{\vec{\nu}} = (\nu_{k_1}, \nu_{k_2}, \ldots, \nu_{k_r})^\top \in \mathbb{N}^r$ with $k_1, k_2, \ldots, k_r > p$. Thus, by the integral approximation,

$$
\begin{aligned}
N_a(\lambda) &\asymp \sum_{\nu_{p+1}=1}^{\infty} \cdots \sum_{\nu_{p+r-1}=1}^{\infty} \sum_{\nu_{p+r}=1}^{\infty} \frac{\prod_{k=p+1}^{p+r} \nu_k^{2ma}}{\left(1 + \lambda \prod_{k=p+1}^{p+r} \nu_k^{2m}\right)^2} \\
&\asymp \int_1^\infty \int_1^\infty \cdots \int_1^\infty \left(1 + \lambda x_{p+1}^b \cdots x_{p+r-1}^b x_{p+r}^b\right)^{-2} dx_{p+1} \cdots dx_{p+r-1} dx_{p+r},
\end{aligned}
$$

where $b = 2m/(2ma+1)$. Let $z_k = x_{p+1}x_{p+2}\cdots x_k$ for $k = p+1, \ldots, p+r$. By using the change of variables to replace $(x_{p+1}, \ldots, x_{p+r})$ by $(z_{p+1}, \ldots, z_{p+r})$ and z_{p+r} by $x = \lambda^{1/b} z_{p+r}$,

$$
\begin{aligned}
N_a(\lambda) &\asymp \int_1^\infty \int_1^{z_{p+r}} \cdots \int_1^{z_{p+2}} \left(1 + \lambda z_{p+r}^b\right)^{-2} z_{p+1}^{-1} \cdots z_{p+r-1}^{-1} dz_{p+1} \cdots dz_{p+r-1} dz_{p+r} \\
&\asymp \int_1^\infty (1 + \lambda z_{p+r}^b)^{-2} (\log z_{p+r})^{r-1} dz_{p+r} \\
&\asymp \lambda^{-1/b} \int_{\lambda^{1/b}}^\infty (1 + x^b)^{-2} \left(\log x - b^{-1} \log \lambda\right)^{r-1} dx \asymp \lambda^{-a-1/2m} \left[\log(1/\lambda)\right]^{r-1},
\end{aligned}
$$

where the last step follows from the fact that $2b > 1$ for any $0 \le a < (2m-1)/(2m)$.

Second, we consider $d - r < p < d$. As discussed in the previous case, the number of combinations of non-1 indices being summed is finite, and the largest terms of the summation (S53) over $\boldsymbol{\vec{\nu}} \in \mathbb{V}$ correspond to the indices $\boldsymbol{\vec{\nu}} = (\nu_{k_1}, \ldots, \nu_{k_{r+p-d}}, \nu_{p+1}, \ldots, \nu_d)^\top \in \mathbb{N}^r$, where the indices $k_1, \ldots, k_{r+p-d} \le p$. Thus, by the integral approximation,

$$
\begin{aligned}
N_a(\lambda) &\asymp \sum_{v_{d-r+1}=1}^{\infty} \cdots \sum_{v_d=1}^{\infty} \frac{\prod_{k=d-r+1}^{d} \nu_k^{2ma} \left(1 + \sum_{k=d-r+1}^{p} \nu_k^2\right)}{\left(1 + \sum_{k=d-r+1}^{p} \nu_k^2 + \lambda \prod_{k=d-r+1}^{d} \nu_k^{2m}\right)^2} \\
&\asymp \int_1^\infty \cdots \int_1^\infty \frac{1 + x_{d-r+1}^{b/m} + \cdots + x_p^{b/m}}{\left(1 + x_{d-r+1}^{b/m} + \cdots + x_p^{b/m} + \lambda x_{d-r+1}^b \cdots x_d^b\right)^2} dx_{d-r+1} \cdots dx_d,
\end{aligned}
$$

where $b = 2m/(2ma+1)$. Set $z_k = x_{p+1}x_{p+2}\cdots x_k$ for $k = p+1, \ldots, d$. By using the change the variables to replace $(x_{p+1}, \ldots, x_d)$ by $(z_{p+1}, \ldots, z_d)$, and z_d by $x = \lambda^{1/b} z_d$, and x by

$u = x_{d-r+1} \cdots x_p \cdot x$. We have

$$N_a(\lambda) \asymp \int_1^\infty \cdots \int_1^\infty \left[\int_1^\infty \int_1^{z_d} \cdots \int_1^{z_{p+2}} x_{d-r+1}^{b/m} \left(1 + x_{d-r+1}^{b/m} + \cdots x_p^{b/m} + \lambda x_{d-r+1}^b \cdots x_p^b z_d^b \right)^{-2} \cdot z_{p+1}^{-1} \cdots z_{d-1}^{-1} dz_{p+1} \cdots dz_{d-1} dz_d \right] dx_{d-r+1} \cdots dx_p$$

$$\asymp \lambda^{-1/b} \int_1^\infty \cdots \int_1^\infty \left[\int_{\lambda^{1/b}}^\infty x_{d-r+1}^{b/m} (1 + x_{d-r+1}^{b/m} + \cdots x_p^{b/m} + x_{d-r+1}^b \cdots x_p^b x^b)^{-2} \cdot \left(\log x - b^{-1} \log \lambda\right)^{d-p-1} dx \right] dx_{d-r+1} \cdots dx_p$$

$$\lesssim \lambda^{-1/b} \int_{\lambda^{1/b}}^\infty \left[\int_1^\infty \cdots \int_1^\infty x_{d-r+1}^{b/m} \left(1 + x_{d-r+1}^{b/m} + \cdots + x_p^{b/m} + u^b \right)^{-2} x_{d-r+1}^{-1} \cdots x_p^{-1} \cdot \left(\log u - \log x_{d-r+1} - \cdots - \log x_p - b^{-1} \log \lambda \right)^{d-p-1} dx_{d-r+1} \cdots dx_p \right] du.$$

By Lemma S14, then for any $0 < \tau < 1$,

$$\left(1 + x_{d-r+1}^{b/m} + x_{d-r+2}^{b/m} + \cdots + x_p^{b/m} + u^b\right)^{-2} \lesssim \left(1 + x_{d-r+2}^{b/m} + \cdots + x_p^{b/m} + u^b\right)^{-1+\tau} \cdot \left(x_{d-r+1}^{b/m}\right)^{-(1+\tau)}.$$

Together with the fact $\int_1^\infty t^{-1-\tau} (\log t)^k dt < \infty$ for any $k < \infty$, we have

$$N_a(\lambda) \lesssim \lambda^{-1/b} \int_{\lambda^{1/b}}^\infty \left[\int_1^\infty \cdots \int_1^\infty \left(1 + x_{d-r+2}^{b/m} + \cdots + x_p^{b/m} + u^b\right)^{-1+\tau} x_{d-r+2}^{-1} \cdots x_p^{-1} \times \left(\log u - \log x_{d-r+2} - \cdots - \log x_p - b^{-1} \log \lambda\right)^{d-p-1} dx_{d-r+2} \cdots dx_p \right] du.$$

Continuing this procedure gives

$$N_a(\lambda) \lesssim \lambda^{-1/b} \int_{\lambda^{1/b}}^\infty \left(1 + u^b\right)^{-(1-\tau)^{p-d+r}} \left(\log u - b^{-1} \log \lambda\right)^{d-p-1} du.$$

Since for any $\epsilon > 0$ and $d - r < p < d$, we know if $\tau < \epsilon/d$,

$$(1-\tau)^{p-d+r} \geq 1 - \tau(p - d + r) \geq 1 - \tau(d-1) > 1 - \epsilon.$$

Hence, for any $0 \leq a < (2m-1)/(2m)$, there exists τ such that $(1-\tau)^{p-d+r} > a + 1/(2m) = 1/b$. Therefore,

$$N_a(\lambda) \lesssim \lambda^{-1/b} \left[\log(1/\lambda)\right]^{d-p-1} = \lambda^{-a-1/2m} \left[\log(1/\lambda)\right]^{d-p-1}.$$

Finally, we consider $p = d$. As argued in the previous two cases, the number of combinations of non-1 indices being summed is finite. Now since $p = d$, by the symmetry of indices,

the largest terms of the summation (S53) over $\vec{\boldsymbol{\nu}} \in \mathbb{V}$ correspond to any combinations of r non-1 indices, for example, the first r indices. Thus, by the integral approximation,

$$\begin{aligned} N_a(\lambda) &\asymp \sum_{\nu_1=1}^{\infty} \cdots \sum_{\nu_{r-1}=1}^{\infty} \sum_{\nu_r=1}^{\infty} \frac{\prod_{k=1}^r \nu_k^{2ma} (1 + \sum_{k=1}^r \nu_k^2)}{(1 + \sum_{k=1}^r \nu_k^2 + \lambda \prod_{k=1}^r \nu_k^{2m})^2} \\ &\asymp \int_1^{\infty} \int_1^{\infty} \cdots \int_1^{\infty} \frac{1 + x_1^{b/m} + \cdots + x_{r-1}^{b/m} + x_r^{b/m}}{\left(1 + x_1^{b/m} + \cdots + x_r^{b/m} + \lambda x_1^b \cdots x_{r-1}^b x_r^b\right)^2} dx_1 \cdots dx_{r-1} dx_r, \end{aligned}$$

where $b = 2m/(2ma+1)$. Observe that if $x_1 \cdots x_{r-1} x_r \lesssim \lambda^{mr/[b(1-mr)]}$, then

$$\lambda x_1^b \cdots x_{r-1}^b x_r^b \lesssim x_1^{b/m} + \cdots + x_{r-1}^{b/m} + x_r^{b/m}.$$

By Lemma S6 with $\beta = 0$ and $\alpha = b/m \leq 2$, we have

$$\begin{aligned} N_a(\lambda) &\asymp \int_{x_1 \cdots x_{r-1} x_r \lesssim \lambda^{mr/[b(1-mr)]}} \left(1 + x_1^{b/m} + \cdots + x_{r-1}^{b/m} + x_r^{b/m}\right)^{-1} dx_1 \cdots dx_{r-1} dx_r \\ &\asymp \begin{cases} \lambda^{\frac{mr}{1-mr}\left(a + \frac{r-2}{2mr}\right)}, \text{ if } r \geq 3; \\ \log(1/\lambda), \text{ if } r = 2, a = 0; \quad \lambda^{\frac{2ma}{1-2m}}, \text{ if } r = 2, 0 < a \leq 1; \\ 1, \text{ if } r = 1, a < \frac{1}{2m}; \quad \log(1/\lambda), \text{ if } r = 1, a = \frac{1}{2m}; \\ \lambda^{\frac{1-2ma}{2m-2}}, \text{ if } r = 1, a > \frac{1}{2m}. \end{cases} \end{aligned} \tag{S54}$$

On the other hand, if $\lambda^{mr/[b(1-mr)]}(x_1 \cdots x_{r-1} x_r)^{-1} = o(1)$, then without loss of generality we assume $x_r = \min\{x_1, \cdots, x_r\}$. Let $z = \lambda^{1/b} x_1 \cdots x_{r-1} x_r$. By changing x_r to z, we have

$$\begin{aligned} N_a(\lambda) &\asymp \int_{\lambda^{mr/[b(1-mr)]}(x_1 \cdots x_{r-1} x_r)^{-1} = o(1)} \\ &\qquad \left(1 + x_1^{b/m} + \cdots + x_r^{b/m} + \lambda x_1^b \cdots x_{r-1}^b x_r^b\right)^{-1} dx_1 \cdots dx_{r-1} dx_r \\ &\lesssim \lambda^{-1/b} \int_{\lambda^{1/[b(1-mr)]} z^{-1} = o(1), \lambda^{-(r-1)/(br)} z^{(r-1)/r} \leq x_1 \cdots x_{r-1} \leq \lambda^{-1/b} z} \\ &\qquad \left(1 + x_1^{b/m} + \cdots + x_{r-1}^{b/m} + z^b\right)^{-1} x_1^{-1} \cdots x_{r-1}^{-1} dx_1 \cdots dx_{r-1} dz \\ &\lesssim \lambda^{-1/b} \int_{\lambda^{1/[b(1-mr)]} z^{-1} = o(1)} \left[\int_{\lambda^{-(r-1)/(br)} z^{(r-1)/r} \leq x_1 \cdots x_{r-1} \leq \lambda^{-1/b} z} \right. \\ &\qquad \left. \left(x_1^{b/m} + \cdots + x_{r-1}^{b/m}\right)^{-\tau} x_1^{-1} \cdots x_{r-1}^{-1} dx_1 \cdots dx_{r-1} \right] z^{b(-1+\tau)} dz \\ &\lesssim \lambda^{-1/b} \int_{\lambda^{1/[b(1-mr)]} z^{-1} = o(1)} \lambda^{\tau/(mr)} z^{-\tau b/(mr)} \cdot z^{b(-1+\tau)} dz = o\left[\lambda^{\frac{mr}{1-mr}\left(a + \frac{r-2}{2mr}\right)}\right], \end{aligned} \tag{S55}$$

where the third step follows from the Lemma S15 for $\beta = -1$ and $\alpha = \tau b/m$. Combining (S54) and (S55), we complete the proof for $p = d$ and this lemma. ∎

Lemma S13 (Bounding the norm of the product of functions)**.** *For any $f, g \in \otimes^d \mathcal{H}_1$, $a > 1/2m$, and $1 \le p \le d$, we have that*

$$\sum_{\vec{\boldsymbol{\nu}} \in \mathbb{N}^d} \left(1 + \frac{\rho_{\vec{\boldsymbol{\nu}}}}{\|\phi_{\vec{\boldsymbol{\nu}}}\|_{L_2}^2}\right)^a \|\phi_{\vec{\boldsymbol{\nu}}}\|_{L_2}^2 \left\langle \frac{\partial f(\mathbf{t})}{\partial t_j} \frac{\partial g(\mathbf{t})}{\partial t_j}, \phi_{\vec{\boldsymbol{\nu}}}(\mathbf{t}) \right\rangle_0^2$$
$$\lesssim \|f\|_{L_2(a+1/m)}^2 \left[\sum_{\vec{\boldsymbol{\nu}} \in \mathbb{N}^d} \left(1 + \frac{\rho_{\vec{\boldsymbol{\nu}}}}{\|\phi_{\vec{\boldsymbol{\nu}}}\|_{L_2}^2}\right)^a \|\phi_{\vec{\boldsymbol{\nu}}}\|_{L_2}^2 \left\langle \frac{\partial g(\mathbf{t})}{\partial t_j}, \phi_{\vec{\boldsymbol{\nu}}}(\mathbf{t}) \right\rangle_0^2 \right].$$

Proof. Recall that $\{\psi_\nu(t)\}_{\nu \ge 1}$ is the trigonometrical basis on $L_2(\mathcal{X})$ and $\phi_{\vec{\boldsymbol{\nu}}}(\cdot)$ is defined in (S13). Write $\psi_{\vec{\boldsymbol{\nu}}}(\mathbf{t}) = \psi_{\nu_1}(t_1)\psi_{\nu_2}(t_2)\cdots\psi_{\nu_d}(t_d)$. Note that

$$\sum_{\vec{\boldsymbol{\nu}} \in \mathbb{N}^d} \left(1 + \frac{\rho_{\vec{\boldsymbol{\nu}}}}{\|\phi_{\vec{\boldsymbol{\nu}}}\|_{L_2}^2}\right)^a \|\phi_{\vec{\boldsymbol{\nu}}}\|_{L_2}^2 \langle f, \phi_{\vec{\boldsymbol{\nu}}}\rangle_0^2 = \sum_{\vec{\boldsymbol{\nu}} \in \mathbb{N}^d} \left(1 + \frac{\rho_{\vec{\boldsymbol{\nu}}}}{\|\phi_{\vec{\boldsymbol{\nu}}}\|_{L_2}^2}\right)^a \left(\int_{\mathcal{X}^d} f\psi_{\vec{\boldsymbol{\nu}}}\right)^2.$$

By Theorem A.2.2 and Corollary A.2.1 in Lin (1998), if $a > 1/2m$, then for any $f, g \in \otimes^d \mathcal{H}_1$,

$$\sum_{\vec{\boldsymbol{\nu}} \in \mathbb{N}^d} \left(1 + \frac{\rho_{\vec{\boldsymbol{\nu}}}}{\|\phi_{\vec{\boldsymbol{\nu}}}\|_{L_2}^2}\right)^a \left(\int_{\mathcal{X}^d} fg\psi_{\vec{\boldsymbol{\nu}}}\right)^2$$
$$\lesssim \left[\sum_{\vec{\boldsymbol{\nu}} \in \mathbb{N}^d} \left(1 + \frac{\rho_{\vec{\boldsymbol{\nu}}}}{\|\phi_{\vec{\boldsymbol{\nu}}}\|_{L_2}^2}\right)^a \left(\int_{\mathcal{X}^d} f\psi_{\vec{\boldsymbol{\nu}}}\right)^2 \right] \left[\sum_{\vec{\boldsymbol{\nu}} \in \mathbb{N}^d} \left(1 + \frac{\rho_{\vec{\boldsymbol{\nu}}}}{\|\phi_{\vec{\boldsymbol{\nu}}}\|_{L_2}^2}\right)^a \left(\int_{\mathcal{X}^d} g\psi_{\vec{\boldsymbol{\nu}}}\right)^2 \right].$$

Thus,

$$\sum_{\vec{\boldsymbol{\nu}} \in \mathbb{N}^d} \left(1 + \frac{\rho_{\vec{\boldsymbol{\nu}}}}{\|\phi_{\vec{\boldsymbol{\nu}}}\|_{L_2}^2}\right)^a \|\phi_{\vec{\boldsymbol{\nu}}}\|_{L_2}^2 \left\langle \frac{\partial f(\mathbf{t})}{\partial t_j} \frac{\partial g(\mathbf{t})}{\partial t_j}, \phi_{\vec{\boldsymbol{\nu}}}(\mathbf{t}) \right\rangle_0^2$$
$$= \sum_{\vec{\boldsymbol{\nu}} \in \mathbb{N}^d} \left(1 + \frac{\rho_{\vec{\boldsymbol{\nu}}}}{\|\phi_{\vec{\boldsymbol{\nu}}}\|_{L_2}^2}\right)^a \left(\int_{\mathcal{X}^d} \frac{\partial f(\mathbf{t})}{\partial t_j} \frac{\partial g(\mathbf{t})}{\partial t_j} \psi_{\vec{\boldsymbol{\nu}}}(\mathbf{t})\right)^2$$
$$\lesssim \left[\sum_{\vec{\boldsymbol{\nu}} \in \mathbb{N}^d} \nu_j^2 \left(1 + \prod_{k=1}^d \nu_k^{2m}\right)^a \left(\int_{\mathcal{X}^d} f(\mathbf{t})\psi_{\vec{\boldsymbol{\nu}}}(\mathbf{t})\right)^2 \right] \left[\sum_{\vec{\boldsymbol{\nu}} \in \mathbb{N}^d} \left(1 + \frac{\rho_{\vec{\boldsymbol{\nu}}}}{\|\phi_{\vec{\boldsymbol{\nu}}}\|_{L_2}^2}\right)^a \left(\int_{\mathcal{X}^d} \frac{\partial g(\mathbf{t})}{\partial t_j} \psi_{\vec{\boldsymbol{\nu}}}(\mathbf{t})\right)^2 \right]$$
$$\le \left[\sum_{\vec{\boldsymbol{\nu}} \in \mathbb{N}^d} \left(1 + \prod_{k=1}^d \nu_k^{2m}\right)^{a+\frac{1}{m}} \left(\int_{\mathcal{X}^d} f(\mathbf{t})\psi_{\vec{\boldsymbol{\nu}}}(\mathbf{t})\right)^2 \right] \left[\sum_{\vec{\boldsymbol{\nu}} \in \mathbb{N}^d} \left(1 + \frac{\rho_{\vec{\boldsymbol{\nu}}}}{\|\phi_{\vec{\boldsymbol{\nu}}}\|_{L_2}^2}\right)^a \left(\int_{\mathcal{X}^d} \frac{\partial g(\mathbf{t})}{\partial t_j} \psi_{\vec{\boldsymbol{\nu}}}(\mathbf{t})\right)^2 \right]$$
$$\asymp \|f\|_{L_2(a+1/m)}^2 \left[\sum_{\vec{\boldsymbol{\nu}} \in \mathbb{N}^d} \left(1 + \frac{\rho_{\vec{\boldsymbol{\nu}}}}{\|\phi_{\vec{\boldsymbol{\nu}}}\|_{L_2}^2}\right)^a \left(\int_{\mathcal{X}^d} \frac{\partial g(\mathbf{t})}{\partial t_j} \psi_{\vec{\boldsymbol{\nu}}}(\mathbf{t})\right)^2 \right].$$

This completes the proof. ■

Lemma S14 (A variant of Young's inequality)**.** *For any $a, b \ge 0$ and $0 < \tau < 1$, we have*

$$(a+b)^{-2} \le \frac{(1-\tau)^{1-\tau}(1+\tau)^{1+\tau}}{4} a^{-(1+\tau)} b^{-(1-\tau)}. \tag{S56}$$

When τ is small, the coefficient $(1-\tau)^{1-\tau}(1+\tau)^{1+\tau}/4$ is close to $1/4$.

Proof. To prove (S56), it is sufficient to show

$$a + b \geq 2(1-\tau)^{-(1-\tau)/2}(1+\tau)^{-(1+\tau)/2}a^{(1+\tau)/2}b^{(1-\tau)/2}.$$

Letting $p = 2/(1+\tau)$, $a' = a^{1/p}$, $b' = [b/(p-1)]^{(p-1)/p}$, the above formula is equivalent to

$$\frac{a'}{p} + \frac{(b')^{p/(p-1)}}{p/(p-1)} \geq a'b',$$

which holds by Young's inequality. This completes the proof. ■

Lemma S15. *Suppose that $\beta \leq -1$ and $\alpha > 0$. Then, as $\Xi \to \infty$,*

$$\int_{x_1 \cdots x_r \geq \Xi, x_k \geq 1} \prod_{k=1}^{r} x_k^{\beta}(x_1^{\alpha} + x_2^{\alpha} + \cdots + x_r^{\alpha})^{-1} dx_1 \cdots dx_r \asymp \Xi^{\beta+1-\alpha/r}.$$

Proof. The proof is similar to the proof for Lemma S6. We omit the details here. ■

S5 Proofs for Section S1

For brevity, we consider the regular lattice $l_1 = \cdots = l_d = l$ and $n = l^d$. Other regular lattices can be shown similarly. Write

$$\psi_1(t) = 1, \quad \psi_{2\nu}(t) = \sqrt{2}\cos 2\pi\nu t, \quad \psi_{2\nu+1}(t) = \sqrt{2}\sin 2\pi\nu t, \tag{S57}$$

for $\nu \geq 1$. As discussed in Section S1, it is without loss of generality to assume that $f_0 : \mathcal{X}^d \mapsto \mathbb{R}$ has a periodic boundaries on $\mathcal{X}^d$. Hence $\{\psi_\nu(t)\}_{\nu \geq 1}$ forms an orthonormal system in $L_2(\mathcal{X})$ and an eigenfunction system for K. For a d-dimensional vector $\vec{\boldsymbol{\nu}} = (\nu_1, \ldots, \nu_d) \in \mathbb{N}^d$, write

$$\psi_{\vec{\boldsymbol{\nu}}}(\mathbf{t}) = \psi_{\nu_1}(t_1) \cdots \psi_{\nu_d}(t_d) \quad \text{and} \quad \lambda_{\vec{\boldsymbol{\nu}}} = \lambda_{\nu_1}\lambda_{\nu_2} \cdots \lambda_{\nu_d}, \tag{S58}$$

where λ_{ν_j}s and $\psi_{\nu_j}(t_j)$s are defined according to the spectral theorem, $j = 1, \ldots, d$. Then, any function $f(\cdot)$ in $\mathcal{H}$ admits the Fourier expansion $f(\mathbf{t}) = \sum_{\vec{\boldsymbol{\nu}} \in \mathbb{N}^d} \theta_{\vec{\boldsymbol{\nu}}}\psi_{\vec{\boldsymbol{\nu}}}(\mathbf{t})$, where $\theta_{\vec{\boldsymbol{\nu}}} = \langle f(\mathbf{t}), \psi_{\vec{\boldsymbol{\nu}}}(\mathbf{t})\rangle_{L_2}$, and $J(f) = \sum_{\vec{\boldsymbol{\nu}} \in \mathbb{N}^d} \lambda_{\vec{\boldsymbol{\nu}}}^{-1}\theta_{\vec{\boldsymbol{\nu}}}^2$. We also write $f_0(\mathbf{t}) = \sum_{\vec{\boldsymbol{\nu}} \in \mathbb{N}^d} \theta_{\vec{\boldsymbol{\nu}}}^0 \psi_{\vec{\boldsymbol{\nu}}}(\mathbf{t})$.

By Page 23 of Wahba (1990), it is known that

$$l^{-1}\sum_{i=1}^{l} \psi_\mu(i/l)\psi_\nu(i/l) = \begin{cases} 1, & \text{if } \mu = \nu = 1, \ldots, l, \\ 0, & \text{if } \mu \neq \nu, \mu, \nu = 1, \ldots, l. \end{cases}$$

Define

$$\vec{\psi}_{\vec{\boldsymbol{\nu}}} = (\psi_{\vec{\boldsymbol{\nu}}}(\mathbf{t}_1), \ldots, \psi_{\vec{\boldsymbol{\nu}}}(\mathbf{t}_n))^\top,$$

where $\{\mathbf{t}_1, \dots, \mathbf{t}_n\}$ are the regular lattice design points. Thus, we have

$$\langle \vec{\psi}_{\vec{\boldsymbol{\nu}}}, \vec{\psi}_{\vec{\boldsymbol{\mu}}} \rangle_n = \begin{cases} 1, & \text{if } \nu_j = \mu_j = 1, \dots, l;\ j = 1, \dots, d, \\ 0, & \text{if there exists some } j \text{ such that } \nu_j \neq \mu_j, \end{cases}$$

where $\langle \cdot, \cdot \rangle_n$ is the empirical inner product in $\mathbb{R}^n$. This implies that $\{\vec{\psi}_{\vec{\boldsymbol{\nu}}} \mid \nu_j = 1, \dots, l; j = 1, \dots, d\}$ form an orthogonal basis in $\mathbb{R}^n$ with respect to the empirical norm $\| \cdot \|_n$. Denote the observed data vectors by $\mathbf{y}^{(0)} = (y_1^{(0)}, \dots, y_n^{(0)})^\top$ and $\mathbf{y}^{(j)} = (y_1^{(j)}, \dots, y_n^{(j)})^\top$, and write

$$\begin{cases} z_{\vec{\boldsymbol{\nu}}}^{(0)} & = \langle \mathbf{y}^{(0)}, \vec{\psi}_{\vec{\boldsymbol{\nu}}} \rangle_n, \\ z_{\nu_1, \dots, 2\nu_j - 1, \dots, \nu_d}^{(j)} & = (2\pi)^{-1} \langle \mathbf{y}^{(j)}, \vec{\psi}_{\nu_1, \dots, 2\nu_j, \dots, \nu_d} \rangle_n, \\ z_{\nu_1, \dots, 2\nu_j, \dots, \nu_d}^{(j)} & = -(2\pi)^{-1} \langle \mathbf{y}^{(j)}, \vec{\psi}_{\nu_1, \dots, 2\nu_j - 1, \dots, \nu_d} \rangle_n, \end{cases} \tag{S59}$$

for $\nu_j = 1, \dots, l$ and $j = 1, \dots, d$. Then, $z_{\vec{\boldsymbol{\nu}}}^{(0)} = \tilde{\theta}_{\vec{\boldsymbol{\nu}}}^0 + \delta_{\vec{\boldsymbol{\nu}}}^{(0)}$ and $z_{\vec{\boldsymbol{\nu}}}^{(j)} = \nu_j \tilde{\theta}_{\vec{\boldsymbol{\nu}}}^0 + \delta_{\vec{\boldsymbol{\nu}}}^{(j)}$, where $\tilde{\theta}_{\vec{\boldsymbol{\nu}}}^0 = \theta_{\vec{\boldsymbol{\nu}}}^0 + \sum_{\mu_j \geq l+1, j=1,\dots,d} \theta_{\vec{\boldsymbol{\mu}}}^0 \langle \vec{\psi}_{\vec{\boldsymbol{\nu}}}, \vec{\psi}_{\vec{\boldsymbol{\mu}}} \rangle_n$. The errors $\delta_{\vec{\boldsymbol{\nu}}}^{(0)}$ satisfy

$$\begin{aligned} \mathbb{E}[\delta_{\vec{\boldsymbol{\nu}}}^{(0)}] &= \frac{1}{n} \sum_{i=1}^n \mathbb{E}[\epsilon_i^{(0)}] \vec{\psi}_{\vec{\boldsymbol{\nu}}}(i) \leq \frac{1}{n} \sqrt{\sum_{i=1}^n \{\mathbb{E}[\epsilon_i^{(0)}]\}^2} \sqrt{\sum_{i=1}^n \vec{\psi}_{\vec{\boldsymbol{\nu}}}^2(i)} = o(n^{-1/2}), \\ \mathrm{Var}[\delta_{\vec{\boldsymbol{\nu}}}^{(0)}] &= \frac{1}{n^2} \sum_{i=1}^n \mathrm{Var}[\epsilon_i^{(0)}] \vec{\psi}_{\vec{\boldsymbol{\nu}}}^2(i) + \frac{1}{n^2} \sum_{i \neq i'} \mathrm{Cov}[\epsilon_i^{(0)}, \epsilon_{i'}^{(0)}] \vec{\psi}_{\vec{\boldsymbol{\nu}}}(i) \vec{\psi}_{\vec{\boldsymbol{\nu}}}(i') \\ &\leq \frac{\sigma_0^2}{n} \cdot \frac{1}{n} \sum_{i=1}^n \vec{\psi}_{\vec{\boldsymbol{\nu}}}^2(i) + \frac{2}{n^2} \sum_{i \neq i'} \mathrm{Cov}[\epsilon_i^{(0)}, \epsilon_{i'}^{(0)}] \\ &= O(n^{-1}) + \frac{2}{n^2} \sum_{i \neq i'} o(|i - i'|^{-\Upsilon}) = O(n^{-1}) + o(n^{-1}) = O(n^{-1}). \end{aligned}$$

Similarly for any j, $\delta_{\vec{\boldsymbol{\nu}}}^{(j)}$s have mean $o(n^{-1/2})$ and covariances $O(n^{-1})$.

S5.1 Proof of Theorem S1

We now prove the lower bound under the regular lattices. By the data transformation (S59), it suffices to show the optimal rate in a special case

$$\begin{cases} z_{\vec{\boldsymbol{\nu}}}^{(0)} & = \theta_{\vec{\boldsymbol{\nu}}}^0 + \delta_{\vec{\boldsymbol{\nu}}}^{(0)}, \\ z_{\vec{\boldsymbol{\nu}}}^{(j)} & = \nu_j \theta_{\vec{\boldsymbol{\nu}}}^0 + \delta_{\vec{\boldsymbol{\nu}}}^{(j)}, \quad \text{for } 1 \leq j \leq p, \end{cases} \tag{S60}$$

where $\delta_{\vec{\boldsymbol{\nu}}}^{(j)} \sim \mathcal{N}(0, \sigma_j^2/n)$ are independent. For any $\vec{\boldsymbol{\nu}} \in \mathbb{N}^d$, if we have the prior that $|\tilde{\theta}_{\vec{\boldsymbol{\nu}}}^0| \leq \pi_{\vec{\boldsymbol{\nu}}}$, then the minimax linear estimator is

$$\widehat{\theta}_{\vec{\boldsymbol{\nu}}}^L = \frac{\sigma_0^{-2} z_{\vec{\boldsymbol{\nu}}}^{(0)} + \sum_{j=1}^p \sigma_j^{-2} \nu_j z_{\vec{\boldsymbol{\nu}}}^{(j)}}{n^{-1} \pi_{\vec{\boldsymbol{\nu}}}^{-2} + \sigma_0^{-2} + \sum_{j=1}^p \sigma_j^{-2} \nu_j^2},$$

and the minimax linear risk is

$$n^{-1}\left[n^{-1}\pi_{\vec{\boldsymbol{\nu}}}^{-2}+\sigma_0^{-2}+\sum_{j=1}^{p}\sigma_j^{-2}\nu_j^2\right]^{-1}.$$

By Lemma 6 and Theorem 7 in Donoho et al. (1990), if σ_j^2s are known, the minimax risk of estimating $\theta_{\vec{\boldsymbol{\nu}}}^0$ under the model (S60) is larger than 80% of the minimax linear risk of the hardest rectangle subproblem, and the latter linear risk is

$$R^L=n^{-1}\max_{\sum_{\vec{\boldsymbol{\nu}}\in\mathbb{V}}(1+\lambda_{\vec{\boldsymbol{\nu}}})\pi_{\vec{\boldsymbol{\nu}}}^2=1}\sum_{\vec{\boldsymbol{\nu}}\in\mathbb{V}}\left[n^{-1}\pi_{\vec{\boldsymbol{\nu}}}^{-2}+\sigma_0^{-2}+\sum_{j=1}^{p}\sigma_j^{-2}\nu_j^2\right]^{-1}, \tag{S61}$$

where $\lambda_{\vec{\boldsymbol{\nu}}}$ is the product of eigenvalues in (S58) and recall that the set V is defined in (S3).

We use the Lagrange multiplier method to find $\pi_{\vec{\boldsymbol{\nu}}}^2$ for solving (S61). Let a be the scalar multiplier and define

$$L(\pi_{\vec{\boldsymbol{\nu}}}^2,a)=\sum_{\vec{\boldsymbol{\nu}}\in\mathbb{V}}\left[n^{-1}\pi_{\vec{\boldsymbol{\nu}}}^{-2}+\sigma_0^{-2}+\sum_{j=1}^{p}\sigma_j^{-2}\nu_j^2\right]^{-1}-a(1+\lambda_{\vec{\boldsymbol{\nu}}})\pi_{\vec{\boldsymbol{\nu}}}^2.$$

Taking partial derivative with respect to $\pi_{\vec{\boldsymbol{\nu}}}^2$ gives

$$\frac{\partial L}{\partial\pi_{\vec{\boldsymbol{\nu}}}^2}=n^{-1}\left[n^{-1}+\left(\sigma_0^{-2}+\sum_{j=1}^{p}\sigma_j^{-2}\nu_j^2\right)\pi_{\vec{\boldsymbol{\nu}}}^2\right]^{-2}-a(1+\lambda_{\vec{\boldsymbol{\nu}}})=0.$$

This implies

$$\widehat{\pi}_{\vec{\boldsymbol{\nu}}}^2=\left(\sigma_0^{-2}+\sum_{j=1}^{p}\sigma_j^{-2}\nu_j^2\right)^{-1}\left[b(1+\lambda_{\vec{\boldsymbol{\nu}}})^{-1/2}-n^{-1}\right]_+,$$

where $b=(na)^{-1/2}$. On one hand, plugging the above formula into the constraint $\sum_{\vec{\boldsymbol{\nu}}\in\mathbb{V}}(1+\lambda_{\vec{\boldsymbol{\nu}}})\pi_{\vec{\boldsymbol{\nu}}}^2=1$ gives,

$$\sum_{\vec{\boldsymbol{\nu}}\in\mathbb{V}}\prod_{k=1}^{d}\nu_k^{2m}\left(\sigma_0^{-2}+\sum_{j=1}^{p}\sigma_j^{-2}\nu_j^2\right)^{-1}\left[b\prod_{k=1}^{d}\nu_k^{-m}-n^{-1}\right]_+\asymp 1.$$

By restricting $\prod_{j=1}^{d}\nu_j\leq(nb)^{1/m}$, this becomes

$$\sum_{\vec{\boldsymbol{\nu}}\in\mathbb{V},\prod_{k=1}^{d}\nu_k\leq(nb)^{1/m}}\left(\sigma_0^{-2}+\sum_{j=1}^{p}\sigma_j^{-2}\nu_j^2\right)^{-1}\left(b\prod_{k=1}^{d}\nu_k^{m}-n^{-1}\prod_{k=1}^{d}\nu_k^{2m}\right)\asymp 1. \tag{S62}$$

On the other hand, the linear risk in (S61) can be written as

$$R^L \asymp n^{-1} \sum_{\vec{\boldsymbol{\nu}} \in \mathbb{V}, \prod_{k=1}^d \nu_k \le (nb)^{1/m}} \left(1 - \frac{1}{nb}\prod_{k=1}^d \nu_k^m\right) \times \left(\sigma_0^{-2} + \sum_{j=1}^p \sigma_j^{-2}\nu_j^2\right)^{-1}. \tag{S63}$$

We discuss for R^L in the above (S63) under the condition (S62) for three cases with $0 \le p \le d-r$, $d-r < p < d$ and $p = d$.

If $0 \le p \le d-r$, since $\vec{\boldsymbol{\nu}} \in \mathbb{V}$, there are at most r of $\nu_1, \ldots, \nu_d$ not equal to 1, which implies that the number of combinations of non-1 indices being summed in (S62) is no greater than $C_d^1 + C_d^2 + \cdots + C_d^r < \infty$. Due to the term $(\sigma_0^{-2} + \sum_{j=1}^p \sigma_j^{-2}\nu_j^2)^{-1}$, the largest terms of the summation (S62) over $\vec{\boldsymbol{\nu}} \in \mathbb{V}$ correspond to the combinations of indices whereas fewer $\nu_1, \ldots, \nu_p$ being summed as possible, for example, $v_k \equiv 1$ for $k \le p$ and $k > p+r$, and $(\nu_{p+1}, \ldots, \nu_{p+r}) \in \mathbb{N}^r$ are non-1. Thus, (S62) is equivalent to

$$\sum_{\prod_{k=1}^r \nu_{p+k} \le (nb)^{1/m}} \left(b\prod_{k=1}^r \nu_{p+k}^m - n^{-1}\prod_{k=1}^r \nu_{p+k}^{2m}\right) \asymp 1.$$

Using the integral approximation, we have

$$\int_{\prod_{k=1}^r x_{p+k} \le (nb)^{1/m}, x_{p+k} \ge 1} \left(b\prod_{k=1}^r x_{p+k}^m - \frac{1}{n}\prod_{k=1}^r x_{p+k}^{2m}\right) dx_{p+1}\cdots dx_{p+r} \asymp 1.$$

By letting $z_j = \prod_{1 \le k \le j} x_{p+k}$, $j = 1, 2, \ldots, r$, we have

$$\int_1^{(nb)^{1/m}} \left[\int_1^{z_r} \cdots \int_1^{z_2} \left(bz_r^m - \frac{1}{n}z_r^{2m}\right) z_1^{-1}\cdots z_{r-1}^{-1} dz_1 \cdots dz_{r-1}\right] dz_r \asymp 1,$$

where the left-hand side term is the order of $n^{(m+1)/m} b^{(2m+1)/m} [\log(nb)]^{r-1}$ and hence

$$b \asymp n^{-(m+1)/(2m+1)} (\log n)^{-m(r-1)/(2m+1)}. \tag{S64}$$

The linear risk in (S63) becomes

$$\begin{aligned} R^L &\asymp n^{-1} \int_{\prod_{k=1}^r x_{p+k} \le (nb)^{1/m}, x_{p+k} \ge 1} \left(1 - \frac{1}{nb}\prod_{k=1}^r x_{p+k}^m\right) \\ &\asymp [\log(nb)]^{r-1} n^{-1+1/m} b^{1/m} \asymp [n(\log n)^{1-r}]^{-2m/(2m+1)}, \end{aligned}$$

where the last step is by (S64).

If $d - r < p < d$, as discussed in the previous case, the number of combinations of non-1 indices being summed is finite, and the largest terms of the summation (S62) over $\vec{\boldsymbol{\nu}} \in \mathbb{V}$

correspond to the combinations of indices whereas fewer than $\nu_1, \ldots, \nu_p$ being summed as possible, for example, $v_k \equiv 1$ for $k \leq d-r$, and $(\nu_{d-r+1}, \ldots, \nu_d) \in \mathbb{N}^r$ are non-1. Thus, (S62) is equivalent to

$$\sum_{\prod_{k=1}^r \nu_{d-r+k} \leq (nb)^{1/m}} \left(b \prod_{k=1}^r \nu_{d-r+k}^m - n^{-1} \prod_{k=1}^r \nu_{d-r+k}^{2m} \right) \left(1 + \sum_{j=d-r+1}^p \nu_j^2 \right)^{-1} \asymp 1.$$

Using the integral approximation, we have

$$\int_{\prod_{k=1}^r x_{d-r+k} \leq (nb)^{1/m}, x_{d-r+k} \geq 1} \left(b \prod_{k=1}^r x_{d-r+k}^m - n^{-1} \prod_{k=1}^r x_{d-r+k}^{2m} \right) \times \left(1 + \sum_{j=d-r+1}^p x_j^2 \right)^{-1} dx_{d-r+1} \cdots dx_d \asymp 1.$$

By letting $z_j = x_{p+1} x_{p+2} \cdots x_j$, $j = p+1, \ldots, d$, we get

$$\begin{aligned}
1 &\asymp \int_{x_{d-r+1} \cdots x_p z_d \leq (nb)^{1/m}} \left[\int_1^{z_d} \cdots \int_1^{z_{p+2}} \right. \\
&\quad \left(b x_{d-r+1}^m \cdots x_p^m z_d^m - \frac{1}{n} x_{d-r+1}^{2m} \cdots x_p^{2m} z_d^{2m} \right) z_{p+1}^{-1} \cdots z_{d-1}^{-1} \\
&\quad \left. \times \left(1 + x_{d-r+1}^2 + \cdots + x_p^2 \right)^{-1} dz_{p+1} \cdots dz_{d-1} \right] dx_{d-r+1} \cdots dx_p dz_d \\
&= \int_{x_{d-r+1} \cdots x_p z_d \leq (nb)^{1/m}} b x_{d-r+1}^m \cdots x_p^m z_d^m \left(1 - \frac{1}{nb} x_{d-r+1}^m \cdots x_p^m z_d^m \right) \\
&\quad \times (\log z_d)^{d-p-1} \left(1 + x_{d-r+1}^2 + \cdots + x_p^2 \right)^{-1} dx_{d-r+1} \cdots dx_p dz_d \\
&\asymp [\log(nb)]^{d-p-1} n^{1+1/m} b^{2+1/m}.
\end{aligned}$$

The last step is by Lemma S16. Hence,

$$b \asymp n^{-(m+1)/(2m+1)} (\log n)^{-m(d-p-1)/(2m+1)}. \tag{S65}$$

The linear risk in (S63) becomes

$$\begin{aligned}
R^L &\asymp n^{-1} \int_{\prod_{k=d-r+1}^d x_k \leq (nb)^{1/m}, x_k \geq 1} \left(1 - \frac{1}{nb} x_{d-r+1}^m \cdots x_d^m \right) \\
&\quad \cdot (1 + x_{d-r+1}^2 + \cdots + x_p^2)^{-1} dx_{d-r+1} \cdots dx_d \\
&\asymp n^{-1} \int_{x_{d-r+1} \cdots x_p z_d \leq (nb)^{1/m}} \left(1 - \frac{1}{nb} x_{d-r+1}^m \cdots x_p^m z_d^m \right) (\log z_d)^{d-p-1} \\
&\quad \cdot (1 + x_{d-r+1}^2 + \cdots + x_p^2)^{-1} dx_{d-r+1} \cdots dx_p dz_d \\
&\asymp [\log(nb)]^{d-p-1} n^{-1+1/m} b^{1/m},
\end{aligned}$$

where the second step uses the same change of variables by letting $z_j = x_{p+1}x_{p+2}\cdots x_j$, $j = p+1, \ldots, d$, and the last step is by Lemma S16. By (S65), we have

$$R^L \asymp [n(\log n)^{1+p-d}]^{-2m/(2m+1)}.$$

If $p = d$, as discussed in the previous two cases, the number of combinations of non-1 indices being summed is finite, and the largest terms of the summation (S62) over $\vec{\boldsymbol{\nu}} \in \mathbb{V}$ correspond to any combinations of r non-1 indices, for example, $\nu_k \equiv 1$ for $k \geq r+1$, and $(\nu_1, \ldots, \nu_r) \in \mathbb{N}^r$. Thus, (S62) is equivalent to

$$\sum_{\prod_{k=1}^r \nu_k \leq (nb)^{1/m}} \left(b \prod_{k=1}^r \nu_k^m - n^{-1} \prod_{k=1}^r \nu_k^{2m} \right) \left(1 + \sum_{k=1}^r \nu_k^2 \right)^{-1} \asymp 1.$$

Using the integral approximation, we have

$$\begin{aligned} 1 &\asymp \int_{\prod_{k=1}^r x_k \leq (nb)^{1/m}, x_k \geq 1} \left(b \prod_{k=1}^r x_k^m - n^{-1} \prod_{k=1}^r x_k^{2m} \right) \left(1 + \sum_{k=1}^r x_k^2 \right)^{-1} dx_1 \cdots dx_r \\ &\asymp \int_{\prod_{k=1}^r x_k \leq (nb)^{1/m}, x_k \geq 1} b \prod_{k=1}^r x_k^m \left(1 + \sum_{k=1}^r x_k^2 \right)^{-1} dx_1 \cdots dx_r \end{aligned}$$

By letting $\beta = m > 1$ and $\alpha = 2$ in Lemma S6, we have for any $r \geq 1$,

$$b \asymp n^{-(mr+r-2)/(2mr+r-2)}. \tag{S66}$$

The linear risk in (S63) becomes

$$\begin{aligned} R^L &\asymp n^{-1} \int_{\prod_{k=1}^r x_k \leq (nb)^{1/m}, x_k \geq 1} \left(1 - \frac{1}{nb} x_1^m \cdots x_r^m \right) \\ &\qquad \cdot (1 + x_1^2 + \cdots + x_r^2)^{-1} dx_1 \cdots dx_r \\ &\asymp n^{-1} \int_{\prod_{k=1}^r x_k \leq (nb)^{1/m}, x_k \geq 1} (1 + x_1^2 + \cdots + x_r^2)^{-1} dx_1 \cdots dx_r \\ &\asymp \left[n^{-1} (nb)^{(r-2)/(mr)} \right] \mathbb{1}_{r \geq 3} + \left[n^{-1} \log(nb) \right] \mathbb{1}_{r=2} + \left(n^{-1} \right) \mathbb{1}_{r=1}, \end{aligned}$$

where the last step uses Lemma S6 with $\beta = 0$ and $\alpha = 2$. By (S66), we have

$$R^L \asymp \left[n^{-(2mr)/[(2m+1)r-2]} \right] \mathbb{1}_{r \geq 3} + \left[n^{-1} \log(n) \right] \mathbb{1}_{r=2} + n^{-1} \mathbb{1}_{r=1},$$

where the constant factor does not depend on n. This completes the proof.

S5.2 Proof of Theorem S2

We now prove the theorem for only $r = d$ and $p = d - 1$. Other settings can be shown similarly. Using the discrete transformed data (S59), the estimator $\widehat{f}_n$ in (9) can be obtained through

$$\widehat{\theta}_{\vec{\boldsymbol{\nu}}} = \arg\min_{\tilde{\theta}_{\vec{\boldsymbol{\nu}}} \in \mathbb{R}} \left\{ \frac{1}{n(p+1)} \left[\frac{1}{\sigma_0^2} \sum_{\vec{\boldsymbol{\nu}} \in V, \|\vec{\boldsymbol{\nu}}\|_{\min} \le l} \left(z_{\vec{\boldsymbol{\nu}}}^{(0)} - \theta_{\vec{\boldsymbol{\nu}}} \right)^2 \right.\right.$$

$$\left.\left. + \sum_{j=1}^{p} \frac{1}{\sigma_j^2} \sum_{\vec{\boldsymbol{\nu}} \in V, \|\vec{\boldsymbol{\nu}}\|_{\min} \le l} \left(z_{\vec{\boldsymbol{\nu}}}^{(j)} - \nu_j \theta_{\vec{\boldsymbol{\nu}}} \right)^2 \right] + \lambda \sum_{\vec{\boldsymbol{\nu}} \in V, \|\vec{\boldsymbol{\nu}}\|_{\min} \le l} \lambda_{\vec{\boldsymbol{\nu}}} \theta_{\vec{\boldsymbol{\nu}}}^2 \right\}$$

and $\widehat{f}_n(\mathbf{t}) = \sum_{\vec{\boldsymbol{\nu}} \in \mathbb{V}, \|\vec{\boldsymbol{\nu}}\|_{\min} \le l} \widehat{\theta}_{\vec{\boldsymbol{\nu}}} \psi_{\vec{\nu}}(\mathbf{t})$, where $\mathbb{V}$ is defined in (S3). Direct calculations give

$$\widehat{\theta}_{\vec{\boldsymbol{\nu}}} = \frac{\sigma_0^{-2} z_{\vec{\boldsymbol{\nu}}}^{(0)} + \sum_{j=1}^{p} \sigma_j^{-2} \nu_j z_{\vec{\boldsymbol{\nu}}}^{(j)}}{\sigma_0^{-2} + \sum_{j=1}^{p} \sigma_j^{-2} \nu_j^2 + \lambda \lambda_{\vec{\boldsymbol{\nu}}}^{-1}}.$$

The deterministic error of $\widehat{f}_n$ can be analyzed in two parts. On one hand, since $f_0 \in \mathcal{H}$ and $\lambda_\nu \asymp \nu^{-2m}$, we know $\sum_{\vec{\boldsymbol{\nu}} \in \mathbb{V}, \|\vec{\boldsymbol{\nu}}\|_{\min} \ge l+1} (\theta_{\vec{\boldsymbol{\nu}}}^0)^2 \asymp n^{-2m}$. This is the truncation error due to $\widehat{\theta}_{\vec{\boldsymbol{\nu}}} = 0$ for $\nu_k \ge l + 1$, $1 \le k \le d$. On the other hand, note that $\langle \vec{\psi}_{\vec{\boldsymbol{\nu}}}, \vec{\psi}_{\vec{\boldsymbol{\mu}}} \rangle_n^2 \le 1$ and then

$$\left(\sum_{\vec{\boldsymbol{\mu}} \in \mathbb{V}, \|\vec{\boldsymbol{\mu}}\|_{\min} \ge l+1} \theta_{\vec{\boldsymbol{\mu}}}^0 \langle \vec{\psi}_{\vec{\boldsymbol{\nu}}}, \vec{\psi}_{\vec{\boldsymbol{\mu}}} \rangle_n \right)^2 \le \sum_{\vec{\boldsymbol{\mu}} \in \mathbb{V}, \|\vec{\boldsymbol{\mu}}\|_{\min} \ge l+1} (\theta_{\vec{\boldsymbol{\mu}}}^0)^2 \asymp n^{-2m}.$$

Thus,

$$\sum_{\vec{\boldsymbol{\nu}} \in \mathbb{V}, \|\vec{\boldsymbol{\nu}}\|_{\min} \le l} \left(\mathbb{E}\widehat{\theta}_{\vec{\boldsymbol{\nu}}} - \theta_{\vec{\boldsymbol{\nu}}}^0 \right)^2$$

$$\lesssim \sum_{\vec{\boldsymbol{\nu}} \in \mathbb{V}, \|\vec{\boldsymbol{\nu}}\|_{\min} \le l} \frac{(\lambda \lambda_{\vec{\boldsymbol{\nu}}}^{-1} \theta_{\vec{\boldsymbol{\nu}}}^0)^2 + [\mathbb{E}\delta_{\vec{\boldsymbol{\nu}}}^{(0)}]^2 + \sum_{j=1}^{p} \nu_j^2 [\mathbb{E}\delta_{\vec{\boldsymbol{\nu}}}^{(j)}]^2}{(\sigma_0^{-2} + \sum_{j=1}^{p} \sigma_j^{-2} \nu_j^2 + \lambda \lambda_{\vec{\boldsymbol{\nu}}}^{-1})^2} + n^{-2m+1}$$

$$\le \lambda^2 \sup_{\vec{\boldsymbol{\nu}} \in \mathbb{V}} \frac{\lambda_{\vec{\boldsymbol{\nu}}}^{-1}}{\left(\sigma_0^{-2} + \sum_{j=1}^{p} \sigma_j^{-2} \nu_j^2 + \lambda \lambda_{\vec{\boldsymbol{\nu}}}^{-1} \right)^2} \sum_{\vec{\boldsymbol{\nu}} \in \mathbb{V}} \lambda_{\vec{\boldsymbol{\nu}}}^{-1} (\theta_{\vec{\boldsymbol{\nu}}}^0)^2$$

$$+ o(n^{-1}) \sum_{\vec{\boldsymbol{\nu}} \in \mathbb{V}, \|\vec{\boldsymbol{\nu}}\|_{\min} \le l} \frac{1 + \sum_{j=1}^{p} \nu_j^2}{(1 + \sum_{j=1}^{p} \nu_j^2 + \lambda \nu_1^{2m} \cdots \nu_d^{2m})^2} + n^{-2m+1}$$

$$\asymp \lambda^2 J(f_0) \sup_{\vec{\boldsymbol{\nu}} \in \mathbb{V}} \frac{\nu_1^{2m} \cdots \nu_d^{2m}}{(1 + \sum_{j=1}^{p} \nu_j^2 + \lambda \nu_1^{2m} \cdots \nu_d^{2m})^2} + o\{n^{-1} \lambda^{-1/2m}\} + n^{-2m+1},$$

where the last step uses Lemma S12 with $a = 0$ and $p = d - 1$. Define that

$$B_\lambda(\vec{\boldsymbol{\nu}}) = \frac{\nu_1^{2m} \cdots \nu_d^{2m}}{(1 + \sum_{j=1}^p \nu_j^2 + \lambda \nu_1^{2m} \cdots \nu_d^{2m})^2}.$$

For the $\sup_{\vec{\boldsymbol{\nu}} \in \mathbb{V}} B_\lambda(\vec{\boldsymbol{\nu}})$ term above, suppose that $\prod_{j=1}^d \nu_j^{2m} > 0$ is fixed and denoted by x^{-1}, then $B_\lambda(\vec{\boldsymbol{\nu}})$ is maximized by letting $\sum_{j=1}^p \nu_j^2$ be as small as possible, where $p = d - 1$. This suggests $\nu_1 = \nu_2 = \cdots = \nu_p = 1$, and

$$\sup_{\vec{\boldsymbol{\nu}} \in \mathbb{V}} B_\lambda(\vec{\boldsymbol{\nu}}) \asymp \sup_{x > 0} \frac{x^{-1}}{(1 + \lambda x^{-1})^2} \asymp \lambda^{-1},$$

where the last step is achieved when $x \asymp \lambda$. Combining all parts of bias gives

$$\sum_{\vec{\boldsymbol{\nu}} \in \mathbb{V}} \left(\mathbb{E}\widehat{\theta}_{\vec{\boldsymbol{\nu}}} - \theta^0_{\vec{\boldsymbol{\nu}}} \right)^2 = O\left\{ \lambda J(f_0) + n^{-2m+1} \right\} + o\{n^{-1}\lambda^{-1/2m}\}. \tag{S67}$$

The constant factor on the upper bound does not depend on n.

The stochastic error is bounded as follows:

$$\begin{aligned}
\sum_{\vec{\boldsymbol{\nu}} \in \mathbb{V}} \mathbb{E} \left(\widehat{\theta}_{\vec{\boldsymbol{\nu}}} - \mathbb{E}\widehat{\theta}_{\vec{\boldsymbol{\nu}}} \right)^2 &= \sum_{\vec{\boldsymbol{\nu}} \in \mathbb{V}, \|\vec{\boldsymbol{\nu}}\|_{\min} \le l} \frac{n^{-1}(\sigma_0^{-2} + \sum_{j=1}^p \sigma_j^{-2}\nu_j^2)}{(\sigma_0^{-2} + \sum_{j=1}^p \sigma_j^{-2}\nu_j^2 + \lambda\lambda_{\vec{\boldsymbol{\nu}}}^{-1})^2} \\
&\lesssim \sum_{\vec{\boldsymbol{\nu}} \in \mathbb{V}, \|\vec{\boldsymbol{\nu}}\|_{\min} \le l} \frac{1 + \sum_{j=1}^p \nu_j^2}{n(1 + \sum_{j=1}^p \nu_j^2 + \lambda \nu_1^{2m} \cdots \nu_d^{2m})^2}.
\end{aligned}$$

Using Lemma S12 with $a = 0$ and $p = d - 1$, we have

$$\sum_{\vec{\boldsymbol{\nu}} \in \mathbb{V}} \mathbb{E} \left(\widehat{\theta}_{\vec{\boldsymbol{\nu}}} - \mathbb{E}\widehat{\theta}_{\vec{\boldsymbol{\nu}}} \right)^2 = O\left\{ n^{-1}\lambda^{-1/2m} \right\}. \tag{S68}$$

Combining (S67) and (S68) and letting $\lambda \asymp n^{-2m/(2m+1)}$ completes the proof.

S5.3 Auxiliary Lemmas for Theorems S1 and S2

Lemma S16. *Suppose that $s \ge 1$, $\beta \ge 0$ and $\beta \ne 1$, and $r \ge 1$. Then as $\Xi \to \infty$,*

$$\int_{x_1 \cdots x_r \cdot z \le \Xi, x_k \ge 1, z \ge 1} x_1^\beta \cdots x_r^\beta z^\beta (\log z)^s (x_1^2 + \cdots + x_r^2)^{-1} dx_1 \cdots dx_r dz \asymp \Xi^{\beta+1} (\log \Xi)^s.$$

Proof. For any $\tau \ge 1$, we have

$$\{1 \le z \le \Xi\tau^{-r}, 1 \le x_k \le \tau, k = 1, \ldots, r\} \subset \{x_1 \cdots x_r \cdot z \le \Xi, z \ge 1, x_k \ge 1, k = 1, \ldots, r\}.$$

Thus, if $\Xi \to \infty$,

$$\begin{aligned}
&\int_{x_1\cdots x_r\cdot z\leq\Xi, x_k\geq 1, z\geq 1} x_1^\beta\cdots x_r^\beta z^\beta(\log z)^s(x_1^2+\cdots+x_r^2)^{-1}dx_1\cdots dx_r dz\\
&\geq \int_1^{\Xi\tau^{-r}}\int_1^\tau\cdots\int_1^\tau z^\beta(\log z)^s x_1^{\beta-2}\cdots x_r^{\beta-2}dx_1\cdots dx_r dz\\
&\asymp \Xi^{\beta+1}\tau^{-r(\beta+1)}(\log\Xi - r\log\tau)^s\tau^{r(\beta-1)}.
\end{aligned}$$

Let $\tau\to 1$, we have $\int_{x_1\cdots x_r\cdot z\leq\Xi, x_k\geq 1, z\geq 1}(\log z)^s(x_1^2+\cdots+x_r^2)^{-1}dx_1\cdots dx_r dz \gtrsim \Xi^{\beta+1}(\log\Xi)^s$.

On the other hand, define $u = x_1\cdots x_r\cdot z$ and change the variable z to u. We have that as $\Xi\to\infty$,

$$\begin{aligned}
&\int_{x_1\cdots x_r\cdot z\leq\Xi, x_k\geq 1, z\geq 1} x_1^\beta\cdots x_r^\beta z^\beta(\log z)^s(x_1^2+\cdots+x_r^2)^{-1}dx_1\cdots dx_r dz\\
&= \int_1^\Xi\int_1^u\int_1^{u/x_r}\cdots\int_1^{u/(x_r x_{r-1}\cdots x_2)} u^\beta(\log u - \log x_r - \cdots - \log x_1)^s\\
&\qquad\cdot\left(x_1^2+\cdots+x_{r-1}^2+x_r^2\right)^{-1}x_1^{-1}\cdots x_{r-1}^{-1}x_r^{-1}dx_1\cdots dx_{r-1}dx_r du\\
&\lesssim \int_1^\Xi\int_1^u\int_1^{u/x_r}\cdots\int_1^{u/(x_r x_{r-1}\cdots x_2)} u^\beta(\log u - \log x_r - \cdots - \log x_1)^s\\
&\qquad\cdot x_1^{-1-2/r}\cdots x_{r-1}^{-1-2/r}x_r^{-1-2/r}dx_1\cdots dx_{r-1}dx_r du\\
&\lesssim \int_1^\Xi u^\beta(\log u)^s du \asymp \Xi^{\beta+1}(\log\Xi)^s.
\end{aligned}$$

The second step is by Lemma S14. This completes the proof. ■

References

Ankenman, B. E., Nelson, B. L., and Staum, J. (2010). Stochastic kriging for simulation metamodeling. *Operations Research*, 58(2):371–382.

Chen, X., Ankenman, B. E., and Nelson, B. L. (2013). Enhancing stochastic kriging metamodels with gradient estimators. *Operations Research*, 61(2):512–528.

Donoho, D. L., Liu, R. C., and MacGibbon, B. (1990). Minimax risk over hyperrectangles, and implications. *The Annals of Statistics*, 18(3):1416–1437.

Efron, B. and Tibshirani, R. J. (1993). *An Introduction to the Bootstrap.* New York: Chapman and Hall.

Frees, E. W. and Valdez, E. A. (1998). Understanding relationships using copulas. *North American Actuarial Journal*, 2(1):1–25.

Gelfand, I. M. and Silverman, R. A. (2000). *Calculus of Variations*. Courier Corporation.

Hall, P. (1992a). Effect of bias estimation on coverage accuracy of bootstrap confidence intervals for a probability density. *The Annals of Statistics*, pages 675–694.

Hall, P. (1992b). On bootstrap confidence intervals in nonparametric regression. *The Annals of Statistics*, pages 695–711.

Hall, P. and Yatchew, A. (2007). Nonparametric estimation when data on derivatives are available. *The Annals of Statistics*, 35(1):300–323.

Hall, P. and Yatchew, A. (2010). Nonparametric least squares estimation in derivative families. *Journal of Econometrics*, 157(2):362–374.

Härdle, W. and Bowman, A. W. (1988). Bootstrapping in nonparametric regression: local adaptive smoothing and confidence bands. *Journal of the American Statistical Association*, 83(401):102–110.

Jones, B. L. and Mereu, J. A. (2002). A critique of fractional age assumptions. *Insurance: Mathematics and Economics*, 30(3):363–370.

Lim, E. (2024). Estimating a function and its derivatives under a smoothness condition. *Mathematics of Operations Research*.

Lin, Y. (1998). Tensor product space anova models in multivariate function estimation. *Thesis (Ph.D.)–University of Pennsylvania*.

Lin, Y. (2000). Tensor product space anova models. *The Annals of Statistics*, 28(3):734–755.

Oden, J. T. and Reddy, J. N. (2012). *An Introduction to the Mathematical Theory of Finite Elements*. New York: John Wiley & Sons.

Rudi, A. and Rosasco, L. (2017). Generalization properties of learning with random features. *Advances in Neural Information Processing Systems (NeurIPS)*, 30.

Ruppert, D., Wand, M. P., and Carroll, R. J. (2003). *Semiparametric Regression*. New York: Cambridge University Press.

Schoenberg, I. J. (1964). Spline functions and the problem of graduation. *Proceedings of the National Academy of Sciences*, 52(4):947–950.

Suri, R. and Leung, Y. T. (1987). Single run optimization of a siman model for closed loop flexible assembly systems. *Proceedings of the 19th Conference on Winter Simulation*, pages 738–748.

Tsybakov, A. B. (2009). *Introduction to Nonparametric Estimation.* New York: Springer.

van der Vaart, A. and Wellner, J. (1996). *Weak Convergence and Empirical Processes.* Springer, New York.

Wahba, G. (1990). *Spline Models for Observational Data.* Philadelphia, PA: SIAM.

Wahba, G., Wang, Y., Gu, C., Klein, R., and Klein, B. (1995). Smoothing spline anova for exponential families, with application to the wisconsin epidemiological study of diabetic retinopathy. *The Annals of Statistics*, 23(6):1865–1895.

Yuan, M. and Cai, T. T. (2010). A reproducing kernel hilbert space approach to functional linear regression. *Annals of Statistics*, 38(6):3412–3444.
